# An MRI radiomics model for predicting a prostate-specific antigen response following abiraterone treatment in patients with metastatic castration-resistant prostate cancer

**DOI:** 10.3389/fonc.2025.1491848

**Published:** 2025-01-27

**Authors:** Yi Wu, Xiang Liu, Shaoxian Chen, Fen Fang, Feng Shi, Yuwei Xia, Zehong Yang, Daiying Lin

**Affiliations:** ^1^ Department of Radiology, Shantou Central Hospital, Shantou, Guangdong, China; ^2^ Department of Medical Imaging, Sun Yat-Sen Memorial Hospital, State Sun Yat-Sen University, Guangzhou, China; ^3^ Shanghai United Imaging Intelligence, Shanghai United Imaging Intelligence, Co. Ltd., Shanghai, China

**Keywords:** neoplasms (prostate), biparametric MRI, radiomics model, abiraterone, metastatic castration-resistant prostate cancer

## Abstract

**Objective:**

To establish a combined radiomics-clinical model for the early prediction of a prostate-specific antigen(PSA) response in patients with metastatic castration-resistant prostate cancer(mCRPC) after treatment with abiraterone acetate(AA).

**Methods:**

The data of a total of 60 mCRPC patients from two hospitals were retrospectively analyzed and randomized into a training group(n=48) or a validation group(n=12). By extracting features from biparametric MRI, including T2-weighted imaging(T2WI), diffusion-weighted imaging(DWI), and apparent diffusion coefficient(ADC) maps, radiomics features from the training dataset were selected using least absolute shrinkage and selection operator(LASSO) regression. Four predictive models were developed to assess the efficacy of abiraterone in treating patients with mCRPC. The primary outcome variable was the PSA response following AA treatment. The performance of each model was evaluated using the area under the receiver operating characteristic curve(AUC). Univariate and multivariate analyses were performed using Cox regression to identify significant predictors of the efficacy of abiraterone treatment in patients with mCRPC.

**Results:**

The integrated model was constructed from seven radiomics features extracted from the T2WI, DWI, and ADC sequence images of the training data. This model demonstrated the highest AUC in both the training and validation cohorts, with values of 0.889 (95% CI, 0.764-0.961) and 0.875 (95% CI, 0.564-0.991). The Rad-score served as an independent predictor of the response to abiraterone treatment in patients with mCRPC (HR: 2.21, 95% CI: 1.01-4.44).

**Conclusion:**

The biparametric MRI-based radiomics model has the potential to predict the PSA response in patients with mCRPC following abiraterone treatment.

**Clinical relevance statement:**

The MRI-based radiomics model could be used to noninvasively identify the AA response in mCRPC patients, which is helpful for early clinical decision-making.

## Introduction

Prostate cancer (PCa) ranks second in incidence among cancers in men worldwide. It is a dynamic disease characterized by changes in tumor biology and the response to specific treatments over time (1). Currently, primary treatments for PCa include radical prostatectomy or androgen deprivation therapy (ADT); however, after 18-36 months of ADT, more than 90% of patients with PCa may eventually experience recurrence and progression to metastatic castration-resistant prostate cancer (mCRPC), which imparts greater risks. Managing mCRPC represents a clinical challenge in the late stages of PCa treatment (2). Novel hormonal therapy drugs, including androgen receptor (AR) antagonists such as abiraterone acetate (AA) and enzalutamide, have been proven effective in treating mCRPC, significantly improving the overall survival (OS) of the patients. However, some patients may develop resistance to these drugs during treatment (3) via mechanisms including androgen receptor splice variants (AR-Vs), amplification or overexpression of AR, AR mutations, ERG gene fusion, and activation of bypass signaling pathways (4–6), all of which require genetic sequencing for assessment. The clinical concern lies in how to detect resistance to AR antagonists earlier and provide effective treatment options for advanced prostate cancer.

Clinically, the effectiveness of mCRPC treatments is monitored through prostate-specific antigen (PSA) levels. The prognostic and/or predictive value of PSA levels and PSA-related parameters in the context of AA therapy period has been investigated in previous studies (7).However, some mCRPC patients experience a “PSA-flare” phenomenon after treatment with AA, underscoring the limitations of solely using the PSA level to evaluate therapeutic effectiveness (8). Moreover, some molecular characterization studies have researched the efficacy and resistance to AA treatment using RNA-based and protein-based methods, like detecting circulating AR-V7 RNA from enrich circulating tumor cells (9). One study has evaluated the whole blood-circulating androgen receptor (AR) transcripts of full length (AR-FL) and one of AR-Vs (AR-V1) can serve as blood-based biomarkers for identification of the primary resistance to AA in castration-resistant prostate cancer patients (10). However, the prediction of response to AA treatment using molecular markers often necessitates the utilization of costly molecular detection techniques.

Radiomics is a computer-based technology that extracts many quantitative imaging features, which are then analyzed in the context of specific clinical issues to assist in decision-making. Radiomics analysis based on MR images has emerged as a promising approach for evaluating heterogeneity in diverse malignancies, including but not limited to breast cancer, lung cancer and prostate cancer (11). Previous studies have successfully utilized radiomics analysis to classify prostate cancer tissues into benign and malignant categories (12). Additionally, some studies have employed radiomics in conjunction with clinical parameters to predict clinically significant prostate cancer (13).

To our knowledge, there is rare research on radiomics to predict the PSA response in patients with mCRPC following AA treatment.

Recent studies have explored the potential correlations between key PCa pathways (apoptosis genes, hypoxia driven genetic changes and especially androgen receptor related genes) and radiomics textural features using pre-biopsy MR images, which enable large-scale characterization and high-throughput data extraction (14). Therefore, we hypothesized that radiomics based on biparametric MRI may hold value in assessing both the efficacy and resistance to AA treatment in patients with mCRPC.

Therefore, the aim of this study was to establish a combined radiomics-clinical model to enable early prediction of the PSA response in mCRPC patients following AA treatment.

## Materials and methods

### Ethics

This retrospective investigation received approval from the Ethics Committees of the respective institutions, and informed consent was waived due to the study’s retrospective design.

### Study population

**Figure 1:**
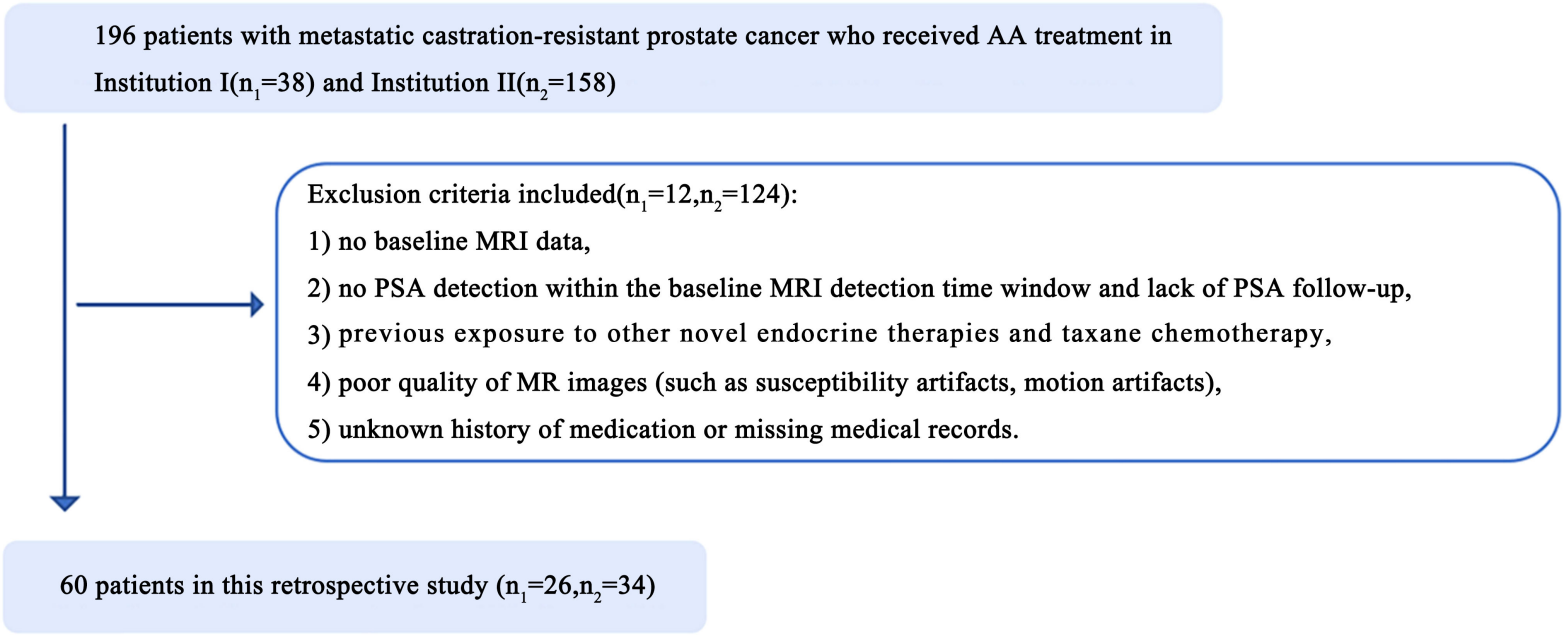
Illustrates the patient enrollment pathway in two institutions. n1, number of patients in institution I; n2, number of patients in institution II.

All 196 patients with metastatic castration-resistant prostate cancer who received AA treatment at Shantou Center Hospital (hereafter, Institution I, 38 patients) and Sun Yat-Sen Memorial Hospital, Sun Yat-Sen University (hereafter, Institution II, 158 patients) from January 2014 to November 2023 were retrospectively enrolled. The patient enrolment process is presented in **Figure 1**. These prostate cancer patients were treated with ADT (Goserelin and Leuprolide) and then further treated with AA.The exclusion criteria included the following: 1) no baseline MRI data, 2) no PSA detection within the baseline MRI detection time window and lack of PSA assessment on follow-up, 3) Previous exposure to other novel endocrine therapies (Enzalutamide, Apalutamide) and taxane chemotherapy (Docetaxel), 4) poor-quality MR images (such as the presence of susceptibility or motion artefacts), and 5) an unknown history of medication or missing medical records. Finally, the data of 60 patients (26 from Institution I, and 34 from Institution II; average age, 69 years) were included in the study and were randomized at an 8:2 ratio into a training group (n=48) or a validation group (n=12). Abiraterone (ZHUORONG;QILU PHARM CO LTD) was given at a dose of 1000 mg daily, with prednisone at a dose of 5 mg twice daily.

### Clinicopathologic patient characteristics

Baseline clinicopathologic data, including patient age, T stage, N stage and PSA level before treatment, were obtained from the electronic medical records systems of the institutions. The PSA density (PSAD) was calculated as the ratio of the PSA level to the prostate volume. Pathologic findings were graded according to the grading system (GS) for PCa of the International Society of Urological Pathology (ISUP) (2014). The patient should undergo continuous follow-up for at least three months after abiraterone treatment review to monitor PSA levels, while ensuring that the results are not affected by the PSA flare phenomenon. The PSA level before abiraterone treatment was recorded as the baseline of the study, and PSA response was defined as a maximum reduction of ≥50% in PSA level from baseline within 3 months after abiraterone treatment. PSA nadir (PSAN) was defined as the first decrease in PSA to the lowest level after androgen deprivation therapy. The PSAN time was defined as the interval from the initiation of ADT to the reaching of the PSAN. The metastatic tumor burden was classified according to the CHAARTED criteria (15). A high tumor burden was defined as ≥4 bone metastases with at least one metastatic lesion located outside the spine or pelvis or concurrent solid organ metastases; otherwise, the patient was considered to have a low tumor burden.

### MRI acquisition and data recording

MRI was conducted using a 3.0-T or 1.5-T MRI scanner (Siemens Magnetom Verio, Siemens Medical Solutions, Erlangen, Germany) with an 8-channel pelvic phased-array coil for receiving signals. The patients were placed supine with the head elevated, and the central positioning line was located 2 cm above the pubic symphysis. The scanning sequences included axial and sagittal T2-weighted imaging (T2WI), axial T1-weighted imaging (T1WI), and axial diffusion weighted imaging (DWI) sequences. Apparent diffusion coefficient (ADC) values were obtained at the postprocessing station (16). The prostate imaging reporting and data system version 2.1 (PI-RADS) scores were calculated and analyzed by 2 experienced imaging physicians (reader 1: F.F., 15 years of prostate lesion expertise; reader 2: Y.W., 9 years of prostate lesion experience) in a double-blind manner.

### Lesion segmentation

**Figure 2:**
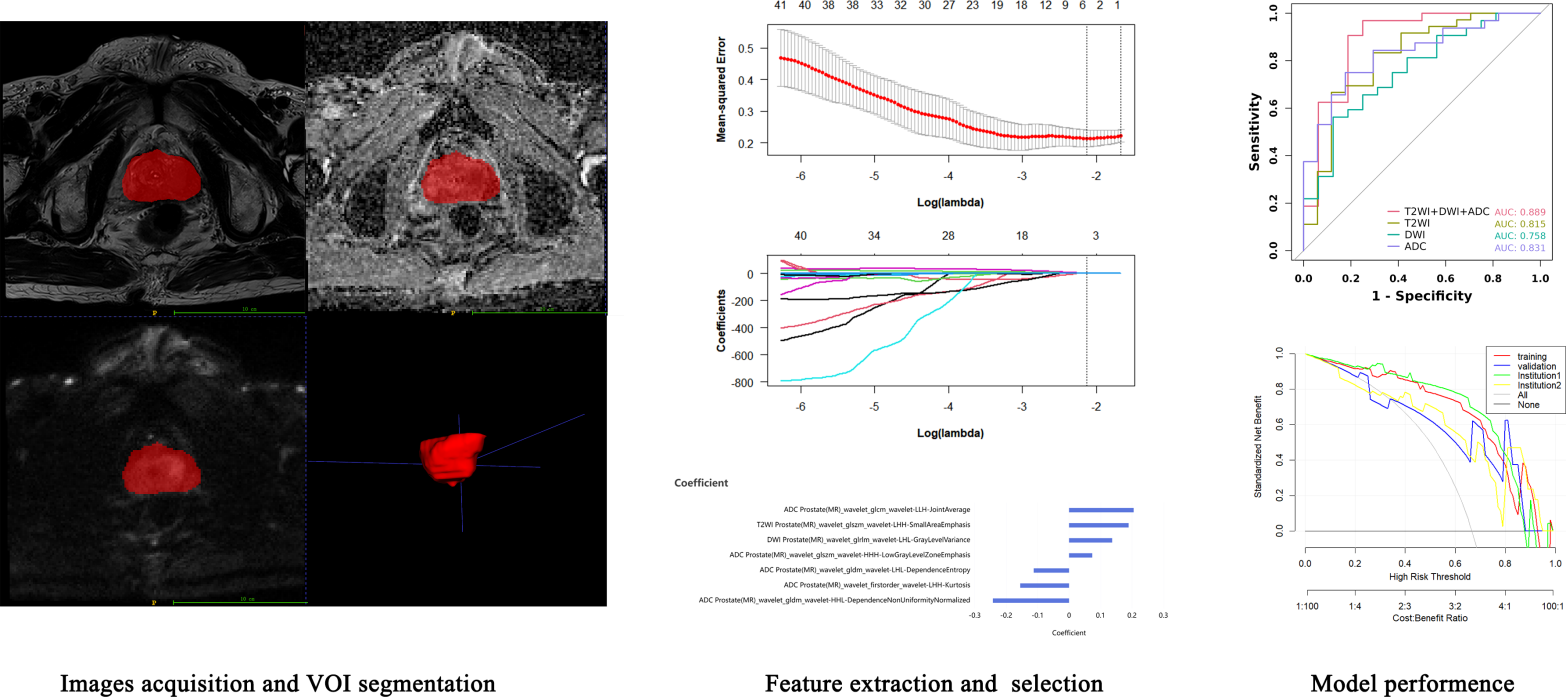
Workflow of radiomics feature acquisition and model analysis.

The radiomics analysis workflow is shown in **Figure 2**. For automatic recognition and segmentation of the whole tumor volume of interest (VOI) on the T2W, DW, and ADC images, we utilized the VB-net prostate cancer segmentation network within the United Imaging Intelligence’s research platform (uAI Research Portal, Version: 20240130, https://urp.united-imaging.com/) (17) and uploaded Dicom images form two institutions. The VB-net network integrates multiple optimization strategies to enhance prostate cancer segmentation performance and expand application scenarios (18). These include an adaptive input module that introduces convolution layers for managing large sized images, ensuring that the network can adapt to diverse input images, and a cascade coarse-to-fine strategy, where a coarse segmentation neural network swiftly locates the prostate and a fine segmentation network is employed for detailed prostate cancer segmentation. The platform homogenizes images from different institutions and automatically identifies prostate images to form a preliminary tumor VOI. Finally, the automatically acquired VOI images were reviewed and finally determined by a senior diagnostic radiologist.

### Feature extraction

Before radiomics feature extraction, all segmented VOIs underwent mean normalization to standardize the distribution of image voxels. Radiomics features were extracted with a version of PyRadiomics (version 3.0.1, https://pyradiomics.readthedocs.io/en/) embedded in the uAI Research Portal. A total of 2160 radiomics features were automatically extracted from the VOI on the T2WI, DWI, and ADC maps of each patient. These features included 18 first-order features, such as the 10th percentile, 90th percentile, energy, entropy, kurtosis, and skewness; 14 shape-based features, such as sphericity, surface area, voxel volume, and maximum 3D diameter; and texture features, including 21 grey level co-occurrence matrix (GLCM) features, 16 grey level run length matrix (GLRLM) features, 16 grey level size zone matrix (GLSZM) features, 5 neighboring grey tone difference matrix (NGTDM) features, and 14 grey level dependent matrix (GLDM) features, which can be used to quantify regional heterogeneity differences. Wavelet filters (LLL, LLH, LHL, LHH, HLL, HLH, HHL, HHH) were also applied to the original images to obtain derived images, from which a total of 2160 derived first-order and texture features were subsequently extracted.

### Feature selection

First, the extracted radiomics features were subjected to z score normalization to eliminate differences in the index dimensions. Second, features with intraclass correlation coefficients (ICCs) ≥ 0.75 were considered reproducible radiomics features and were chosen for further analysis. Finally, the optimal predictive features were obtained with least absolute shrinkage and selection operator (LASSO) (alpha value=0.05), in which fivefold cross-validation was performed to determine the best lambda value. The radiomics signature was quantified as a Rad- score, which was computed using a formula that integrated the chosen radiomics features weighted by their corresponding coefficients obtained from LASSO regression, as follows:


$$Rad-score=\sum_{i}Coefficientt(feature_{i})\times Value(feature_{i})$$

The ICC values of the features (ICC > 0.75) and detailed information regarding the feature selection process, including the number of features retained at each step and their corresponding correlation coefficients, were presented in Appendix excel and **Supplementary Table S2**.

### Model construction

Following LASSO regression analysis for identifying the radiomics features most related to the efficacy of AA treatment in mCRPC, a logistic regression (LR) machine learning framework was used to construct predictive models for the PSA response.

### Model evaluation and verification

The receiver operating characteristic (ROC) curve was constructed, and the area under the curve (AUC), sensitivity, specificity, Youden index and decision curve analysis (DCA) were calculated to assess the model’s performance.

### Construction of the nomogram

To construct the corresponding nomogram, univariate and multivariate Cox regression were applied to filter the clinical data, and a combined radiomics-clinical nomogram model was constructed that included both the clinical risk factors and the Rad-score.

### Statistical analysis

All statistical analyses were performed using R software (version 4.2.2, R Foundation for Statistical Computing, Vienna, Austria, https://www.r-project.org). Continuous variables are presented as the means ± standard deviations, while categorical variables are presented as absolute numbers (n) and their corresponding proportions (%). Univariate and multivariate analyses were performed using Cox regression analysis to identify significant predictors of the efficacy of abiraterone in treating mCRPC. p < 0.05 was considered to indicate statistical significance.

## Results

### Clinical data

In the current study, a cohort of 60 patients was recruited. Based on the PSA response following treatment with abiraterone, 39 patients (65%) were categorized into the response group, while 21 patients (35%) were classified as nonresponders. The clinical characteristics of the patients are shown in **Table 1**. In the group with a PSA response, pathological classification revealed that 19 patients (48.72%) had an ISUP grade of 4 or lower, whereas 20 patients (51.28%) exhibited an ISUP grade above 4. Conversely, in the nonresponse group, 18 patients (85.71%) were classified as having an ISUP grade of 4 or lower, and 3 patients (14.29%) had an ISUP grade above 4; these distributions were significantly different between the groups (P < 0.05). Comparative analysis of additional independent variables—age, PI-RADS score, ADC, T stage, N stage, tumor burden, PSA, PSAD, PSAN, and PSAN time—revealed no statistically significant differences between the two groups.

**Table 1:** Patient demographics and baseline characteristics.

Characteristic	Label			p-value^2^
	Overall, N = 60^1^	Responded, N = 39^1^	Non-responded, N = 21^1^	
Age(year)	69 ± 10	69 ± 10	68 ± 10	0.566
ISUP grade				0.005
≤4	37 (61.67%)	19 (48.72%)	18 (85.71%)	
>4	23 (38.33%)	20 (51.28%)	3 (14.29%)	
PI-RADS				0.298
≤ 4	10 (16.67%)	5 (12.82%)	5 (23.81%)	
> 4	50 (83.33%)	34 (87.18%)	16 (76.19%)	
ADC ((mm^2^/s)*10^-3^)	0.60 ± 0.13	0.58 ± 0.12	0.64 ± 0.14	0.095
T_stage				0.142
T2-3a	16 (26.67%)	8 (20.51%)	8 (38.10%)	
T3b-T4	44 (73.33%)	31 (79.49%)	13 (61.90%)	
N_stage				0.787
N0	30 (50.00%)	19 (48.72%)	11 (52.38%)	
N1	30 (50.00%)	20 (51.28%)	10 (47.62%)	
Tumor_burden				0.533
Low	29 (48.33%)	20 (51.28%)	9 (42.86%)	
High	31 (51.67%)	19 (48.72%)	12 (57.14%)	
PSA(ng/ml)				0.532
<100	14 (23.33%)	8 (20.51%)	6 (28.57%)	
≥100	46 (76.67%)	31 (79.49%)	15 (71.43%)	
PSAD	0.56 (0.22, 1.11)	0.58 (0.24, 1.15)	0.43 (0.17, 1.03)	0.716
PSAN(ng/ml)				0.391
<0.1	10 (16.67%)	8 (20.51%)	2 (9.52%)	
0.1-4	28 (46.67%)	16 (41.03%)	12 (57.14%)	
>4	22 (36.67%)	15 (38.46%)	7 (33.33%)	
PSAN_time(month)	6.4 (4.0, 10.3)	6.9 (4.0, 10.0)	5.0 (3.0, 13.0)	0.565

^1^Mean ± SD; n (%); Median (IQR).

^2^Welch Two Sample t-test; Pearson’s Chi-squared test; Fisher’s exact test; Wilcoxon rank sum test.

### Radiomics signature construction

**Figure 3:**
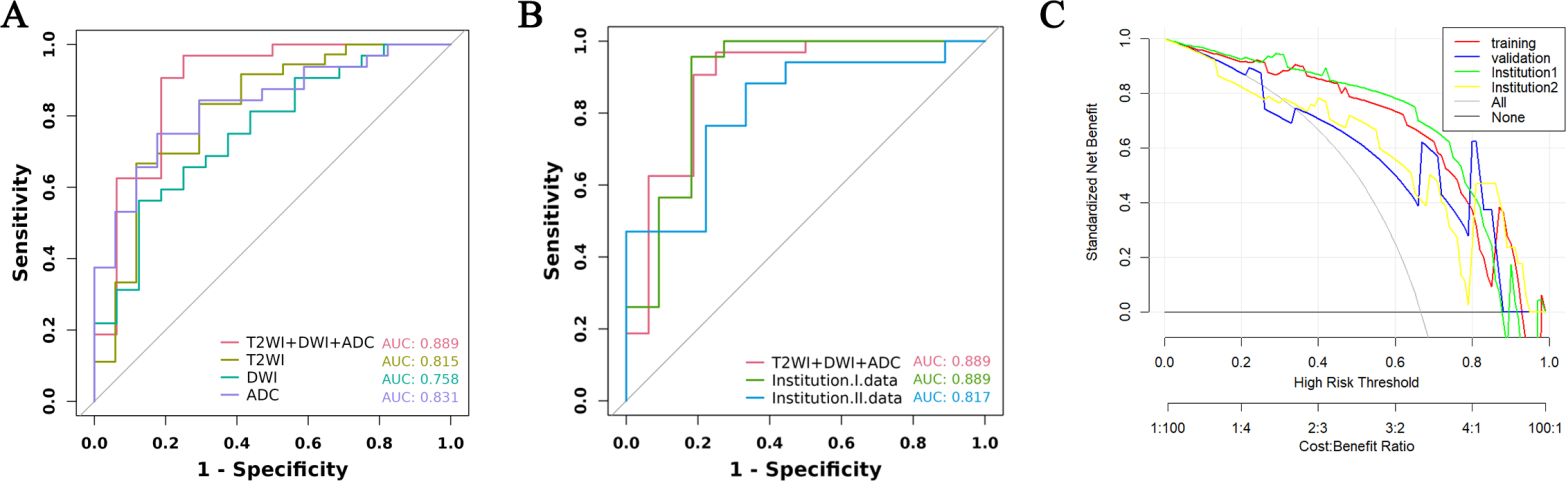
ROC curve and decision curve analysis of different models in training group and validation group. **(A)** ROC of the four models in the training group. **(B)** ROC between the total model of the training group and the two institutions. **(C)** DCA curves of training group, validation group, and two institutions.

From a total of 2160 radiomics features, normalization by the z score and selection through the ICC and LASSO methods led to the selection of 7 radiomics features for the T2WI+DWI+ADC model. Additionally, 5 features were selected for the T2WI model, 4 for the DWI model, and 4 for the ADC model. The coefficients and respective terms of the radiomics features in the LASSO regression model are delineated in **Table 2**. The AUCs of the T2WI+DWI+ADC, T2WI, DWI, and ADC models in the training group were 0.889 (0.764, 0.961), 0.815 (0.685, 0.909), 0.758 (0.613, 0.870), and 0.831 (0.697,0.923), respectively. And those in the validation group were 0.875 (0.564, 0.991), 0.778 (0.471, 0.954), 0.688 (0.368, 0.913), and 0.719 (0.398, 0.930), respectively (**Table 3**). As a result, it was indicated that the T2WI+DWI+ADC and T2WI models had better stability and predictive accuracy, especially the combined model of T2WI+DWI+ADC.Independent validations were also performed on the T2WI+DWI+ADC model using data from two separate institutions, resulting in AUC values of 0.889 (95% CI, 0.734-0.971) and 0.817 (95% CI, 0.617-0.940). Additionally, DCA curves confirmed the clinical decision-making benefits of the model. Plots of the ROC curves and the results of DCA of the models are illustrated in **Figure 3**.

**Table 2:** Performances of predictive models.

Models	Radiomics Feature	Coefficient
T2WI+DWI+ADC	Intercept ADC_wavelet_glcm_wavelet-LLH-JointAverage T2WI_wavelet_glszm_wavelet-LHH-SmallAreaEmphasis DWI_wavelet_glrlm_wavelet-LHL-GrayLevelVariance ADC_wavelet_glszm_wavelet-HHH-LowGrayLevelZoneEmphasis ADC_wavelet_gldm_wavelet-LHL-Dependence Entropy ADC_wavelet_firstorder_wavelet-LHH-Kurtosis ADC_wavelet_gldm_wavelet-HHL-EpendenceNonUniformityNormalized	0.6666667 0.205258653 0.189266831 0.137090683 0.07359337 -0.112405591 -0.155398086 -0.242166355
T2WI	Intercept T2WI_wavelet_glcm_wavelet-HLH-Idmn T2WI_wavelet_firstorder_wavelet-HLH-Median T2WI_wavelet_glcm_wavelet-HLH-JointAverage T2WI_wavelet_ngtdm_wavelet-LLL-Contrast T2WI_wavelet_glszm_wavelet-HHL-SizeZoneNonUniformityNormalized	0.6792453 0.108159378 0.040134195 0.039579168 -0.12266472 -0.126424938
DWI	Intercept	0.6666667
	DWI_wavelet_glszm_wavelet-HHL-GrayLevelVariance	0.118927874
	DWI_wavelet_firstorder_wavelet-HHH-Entropy	0.09541226
	DWI_wavelet_glcm_wavelet-HLH-Idmn	0.058810804
	DWI_wavelet_firstorder_wavelet-LHL-Skewness	-0.101904765
ADC	Intercept	0.6530612
	ADC_wavelet_firstorder_wavelet-LLH-Median	0.0395863
	ADC_wavelet_glszm_wavelet-HHL-SmallAreaEmphasis	-0.0384447239
	ADC_wavelet_firstorder_wavelet-HLH-Mean	-0.0794614255
	ADC_wavelet_glszm_wavelet-LHH-GrayLevelNonUniformityNormalized	-0.109448433

T2WI, T2- weighted imagine; DWI, diffusion weighted imaging; ADC, apparent diffusion coeffificient.

**Table 3:** Performances of predictive models.

Models and datasets	AUC (95% CI)	Sensitivity (95% CI)	Specificity (95% CI)	Youden
T2WI+DWI+ADC				
Training dataset	0.889(0.764 - 0.961)	96.87%(83.8 - 99.9%)	75.00%(47.6 - 92.7%)	0.7188
Validation dataset	0.875(0.564 - 0.991)	75.00%(34.9 - 96.8%)	100.00%(39.8 - 100.0%)	0.7500
Institution I data	0.889(0.734 - 0.971)	95.65%(78.1 - 99.9%)	81.82%(48.2 - 97.7%)	0.7747
Institution II data	0.817(0.617 - 0.940)	88.24%(63.6 - 98.5%)	66.67%(29.9 - 92.5%)	0.5490
T2WI				
Training dataset	0.815(0.685 - 0.909)	66.67%(49.0 - 81.4%)	88.24%(63.6 - 98.5%)	0.5490
Validation dataset	0.778(0.471 - 0.954)	88.89%(51.8 - 99.7%)	75.00%(19.4 - 99.4%)	0.6389
DWI				
Training dataset	0.758(0.613 - 0.870)	56.25%(37.7 - 73.6%)	87.50%(61.7 - 98.4%)	0.4375
Validation dataset	0.688(0.368 - 0.913)	100.00%(63.1 - 100.0%)	50.00%(6.8 - 93.2%)	0.5000
ADC				
Training dataset	0.831(0.697 - 0.923)	75.00%(56.6 - 88.5%)	82.35%(56.6 - 96.2%)	0.5735
Validation dataset	0.719(0.398 - 0.930)	87.50%(47.3 - 99.7%)	75.00%(19.4 - 99.4%)	0.6250

AUC, area under curve.

### Survival analysis

**Figure 4:**
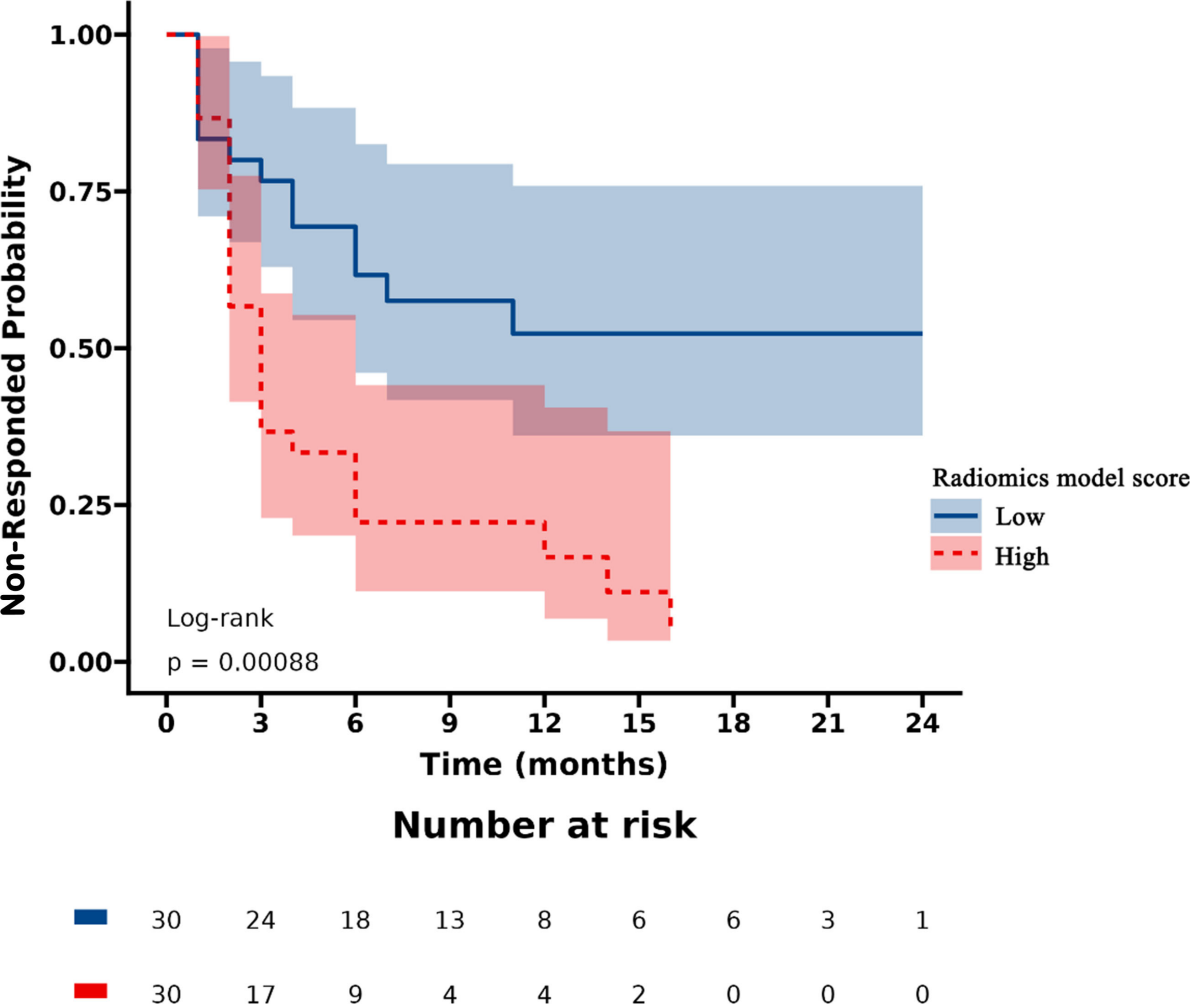
Kaplan-Meier curves of radiomics score in high-risk and low-risk groups.

Patients were stratified into high-risk and low-risk categories according to their radiomics scores. PSA nonresponse curves after abiraterone treatment were generated using the Kaplan‒Meier estimator. The low-risk radiomics score group exhibited a greater likelihood of a PSA nonresponse following AA treatment. Statistically significant differences in nonresponse rates between the different risk groups were identified using the log-rank chi-square test (p < 0.001) (**Figure 4**).

### Univariate and multivariate Cox regression analysis

**Figure 5:**
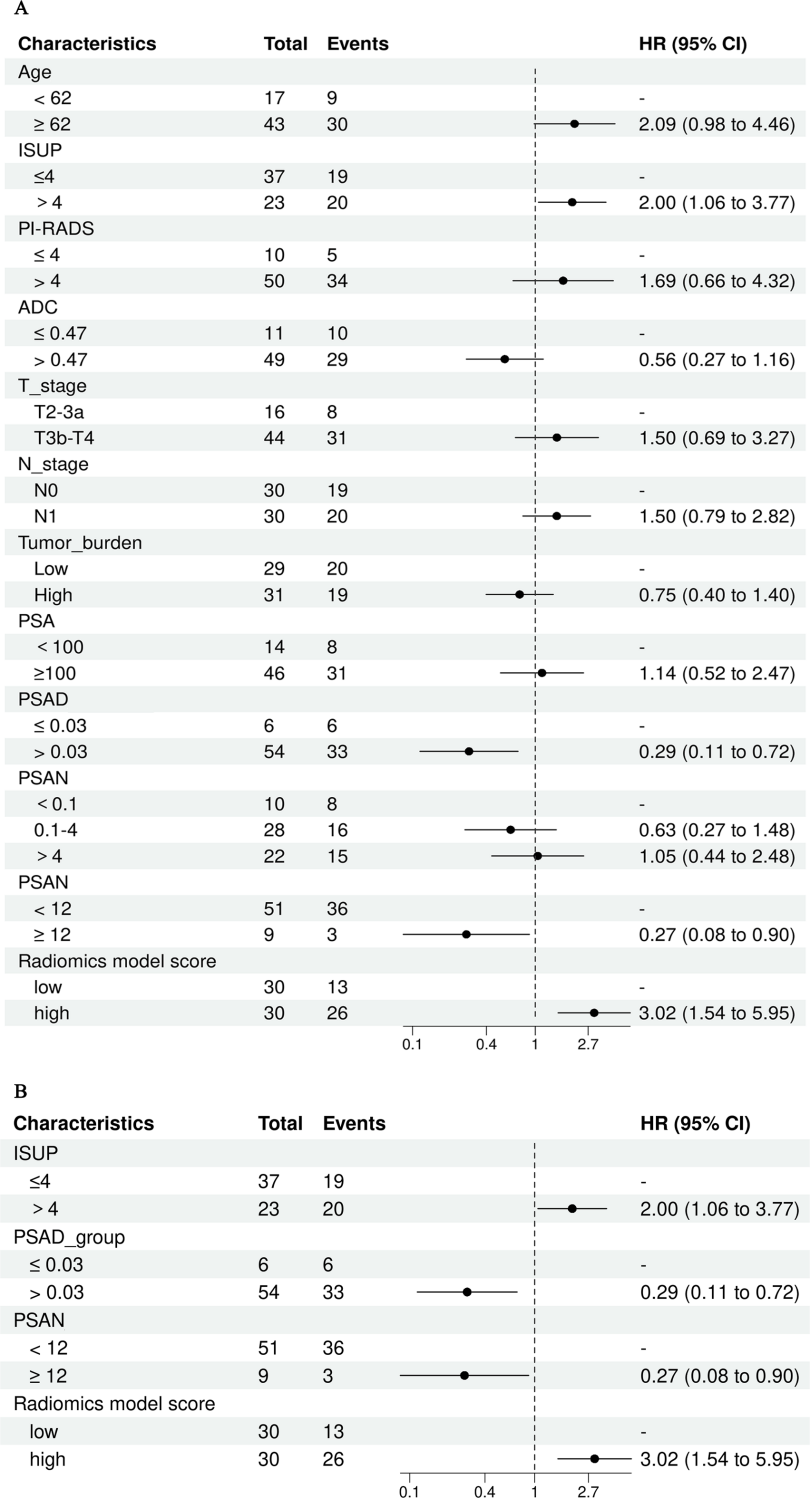
Univariate and multivariate cox regression analysis of abiraterone efficacy, **(A)** displays the univariate Cox regression analysis, while **(B)** shows the multivariate Cox regression analysis. ISUP, International Society of Urological Pathology grade; PI-RADS, The prostate imaging reporting and data system version 2.1 score; PSA, Prostate-Specific Antigen; PSAD, Prostate-Specific Antigen Density; PSAN, Prostate-Specific Antigen nadir.

**Figure 6:**
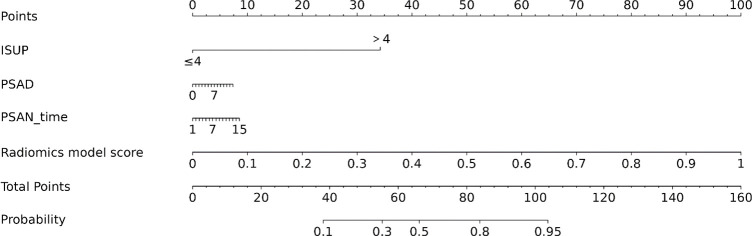
Nomogram of PSA response after abiraterone treatment in mCRPC.

According to univariate Cox regression analysis, the ISUP grade and Rad-score were positively associated with a PSA response (HR: 2.00, 95% CI: 1.06-3.77; HR: 3.02, 95% CI: 1.54-5.95), whereas the PSAD and PSAN time were negatively associated (HR: 0.29, 95% CI: 0.11-0.72; HR: 0.27, 95% CI: 0.08-0.90). Multivariate Cox regression analysis revealed that the Rad-score was an independent predictor of the response to abiraterone treatment in patients with mCRPC (HR: 2.21, 95% CI: 1.01-4.44) (**Figures 5**, **6**; **Table 4**).

**Table 4:** Univariate and multivariate Cox analysis.

Variable	Univariable			Multivariable		
	HR^1^	95% CI^1^	p-value	HR^1^	95% CI^1^	p-value
Age(< 62y vs ≥ 62)	2.09	0.98, 4.46	0.056	—	—	—
ISUP grade (≤4 vs >4)	2.00	1.06, 3.77	0.032	1.38	0.66, 2.87	0.386
PI-RADS(≤4 vs >4)	1.69	0.66, 4.32	0.275	—	—	—
ADC(≤ 0.47 vs > 0.47)	0.56	0.27, 1.16	0.120	—	—	—
T_stage(T2-3a vs T3b-T4)	1.50	0.69, 3.27	0.307	—	—	—
N_stage(N0 vs N1)	1.50	0.79, 2.82	0.213	—	—	—
Tumor_burden(Low vs High)	0.75	0.40, 1.40	0.362	—	—	—
PSA(<100 vs ≥100)	1.14	0.52, 2.47	0.750	—	—	—
PSAD(≤ 0.03 vs > 0.03)	0.29	0.11, 0.72	0.008	0.48	0.17, 1.32	0.155
PSAN(<0.1 vs 0.1-4)	0.63	0.27, 1.48	0.290	—	—	—
(<0.1 vs >4)	1.05	0.44, 2.48	0.917	—	—	—
PSAN_time(< 12 vs ≥ 12)	0.27	0.08, 0.90	0.032	0.41	0.12, 1.42	0.157
Radiomics model score(low vs high)	3.02	1.54, 5.95	0.001	2.12	1.01, 4.44	0.047

^1^HR, Hazard Ratio; CI, Confidence Interval.

## Discussion

Our study found that the biparametric MRI-based radiomics model could predict the PSA response, with AUC values of 0.889 (95% CI, 0.764-0.961) and 0.875 (95% CI, 0.564-0.991), in the training and validation cohorts, respectively. Moreover, the Rad-score was evidenced as an independent predictor of the response to abiraterone treatment in patients with mCRPC (HR: 2.21, 95% CI: 1.01-4.44). The utilization of radiomics models derived from biparametric MRI holds the potential to accurately classify the treatment status of abiraterone and thereby contribute to informed clinical decisions in advanced prostate cancer.

Abiraterone has been demonstrated to enhance radiographic progression-free survival (rPFS) and OS and to significantly postpone clinical deterioration and the initiation of chemotherapy in patients with mCRPC (19). Nevertheless, approximately 20% to 40% of patients exhibit primary resistance to this treatment, as evidenced by unresponsive PSA levels (6). In this study, 21 patients (35%) were identified as having a primary resistance to abiraterone, and commonly used clinical indicators failed to differentiate between PSA responders and non-responders.

Some previous studies have investigated factors to predict primary resistance to AA, in patients with castration resistant prostate cancer (CRPC), However, to our knowledge, AA has been chosen for class 1 recommended drugs for mCRPC without received new endocrine therapy (Enzalutamide, Apalutamide) or chemotherapy rather than non-mCRPC (20). One study found that the value of PSA levels after 1 month of treatment was useful to predict primary resistance to new-generation hormonal agents(abiraterone acetate and enzalutamide)in patients with CRPC (7). However, their analysis included not only patients treated with AA, but also enzalutamide, which might cross interfere the blood PSA level. Anna Katharina Seitz et al. found that testing of AR-V7 mRNA levels in whole blood was a promising approach to predict poor treatment outcome in mCRPC patients receiving abiraterone (n = 56) or enzalutamide(n=29) (21). Qu et al. utilized droplet digital PCR to quantify the mRNA levels of AR-V7 in whole blood samples from mCRPC patients treated with abiraterone (n = 81) or enzalutamide (n = 51) (9). They identified a significant association between AR-V7 mRNA levels and time to treatment failure. Nevertheless, the limitations of their studies were that the aforementioned studies require the use of expensive molecular detection techniques, and the optimal method for determining AR-V7 status has not been determined.

Imaging serves as a crucial clinical tool for tumor diagnosis, staging, and treatment decision-making, although it heavily depends on subjective visual interpretation by physicians, leading to inherent biases and limited data extraction. The use of AI technology reduces acquisition time, enhances image quality, improves prostate cancer detection and risk prediction based on image features, and alleviates radiologists’ workload (22). With advances in clinical information digitization and the burgeoning field of artificial intelligence, radiomics has emerged as a prominent area of research. The spatial and temporal heterogeneity of solid tumors can be noninvasively identified and quantified with radiomics through omics features such as pixel density and spatial distribution. These features may be correlated with tumor aggressiveness, pathological grade, treatment response, and prognostic outcome (23).

MRI-based radiomics has been extensively applied in the diagnosis of prostate cancer, Gleason scoring, and prediction of prostate cancer progression-free survival (PFS), yielding satisfactory outcomes (24–26). However, most of the studies on radiomics of prostate cancer have focused on PCa detection and Gleason score discrimination, while there are few studies on developing radiomics models for predicting primary resistance to abiraterone treatment in patients with mCRPC (27, 28). In this context, we utilized T2WI, DWI, and ADC sequences for feature extraction. T2WI is particularly effective in delineating the anatomical characteristics of prostate cancer tumors, including the involvement of perineural and seminal vesicle spaces, and provides valuable textural features. DWI and ADC values objectively reflect the diffusion of water molecules in biological tissues, which correlates with the degree of malignancy of the tumor. The combination of multiple imaging sequences in radiomics allows a more accurate and comprehensive assessment of tumor information. In our study, the performance of the integrated T2WI, DWI, and ADC sequence model was superior to that of models utilizing individual sequences in predicting the PSA response to abiraterone treatment in mCRPC patients. In the training and validation cohorts, the combined model achieved the highest area under the curve (AUC), with values of 0.889 (95% CI, 0.764–0.961) and 0.875 (95% CI, 0.564–0.991), respectively. To mitigate the impact of interinstitutional variability, independent validations were also conducted with the data from two separate institutions, yielding AUC values of 0.889 (95% CI, 0.734-0.971) and 0.817 (95% CI, 0.617-0.940). Furthermore, DCA validated the clinical decision-making benefits of the model.

Previous studies have confirmed that the ISUP grade is an independent predictor of biochemical recurrence over long-term follow-up after radical prostatectomy and radiotherapy (29, 30). PSA is a vital biomarker for the screening, diagnosis, and evaluation of treatment efficacy in PCa patients. PSA monitoring is integral throughout the entire process of diagnosing and treating PCa (31). Studies have demonstrated that the characteristics of the change in PSA level are significant indicators for assessing the prognosis of patients with mCRPC (32). Ji, G J et al. investigated the ability of PSA level and other risk factors to predict the progression of castration-resistant prostate cancer (CRPC). They observed that clinical T stage, N stage, pre-ADT metastatic status, rate of PSA decrease, and PSAN were significantly correlated with the time to progression of CRPC (33). Furthermore, Sasaki, T et al. confirmed that the time to reach the PSA nadir following initial ADT is a crucial early predictor of both OS and PFS in patients with advanced PCa (34). Consequently, in this study, we incorporated the aforementioned parameters into a Cox regression analysis to evaluate the efficacy of abiraterone treatment. Among them, continuous variables including age, ADC, PSAD and PSAN time were divided into binary variables according to the best cut-off value as defined by the Youden index, while PSA level and the PSAN were categorized according to customary clinical practices. The results of the statistical analysis showed that ISUP grade, PSAD, PSAN time, and Rad-score were significantly associated with the PSA response following abiraterone treatment in mCRPC patients, underscoring their clinical relevance.

Next, in the multivariate Cox regression analysis, the four independent variables that demonstrated significant differences in the univariate Cox regression were further assess. The Rad-score was identified as the only independent predictor for assessing abiraterone effectiveness in patients with mCRPC. This finding suggests that radiomics offers superior predictive power over traditional clinical factors in evaluating the efficacy of abiraterone acetate.

As a noninvasive and robust predictive tool, the radiomics model has the potential to become the useful clinically relevant biomarker for predicting response to Abiraterone hydrochloride(AA) treatment, which can provide valuable guidance for the treatment of advanced prostate cancer. For mCRPC patients exhibiting primary resistance to abiraterone, this approach could direct them towards other therapies (taxanes, immune therapies, radiation therapies, newer targeted agents) (20, 35). If confirmed by larger cohort studies or prospective trials, radiomics assessment could be used to evaluate and compare the efficacy of other therapies in patients with primary abiraterone-resistant, which may improve the treatment of mCRPC in the future.

This study has certain limitations. First, its retrospective design may have led to potential selection biases. Second, it employed a small sample size, which could be related to the limited number of patients with advanced prostate cancer who underwent standardized abiraterone treatment only. Additionally, the study model was only internally validated, and external validation with data from independent institutions is lacking. Our MRI-based radiomics model is still in the preliminary stage, and need to be confirmed by larger cohort studies or prospective trials.

## Conclusion

In summary, our study demonstrated that the proposed biparametric MRI-based radiomics model has potential as a noninvasive tool for predicting the PSA response in patients with mCRPC following abiraterone treatment. This provides an alternative strategy for predicting therapeutic efficacy in advanced prostate cancer patients.

## Data Availability

The original contributions presented in the study are included in the article/supplementary material. Further inquiries can be directed to the corresponding authors.
