# Supplementary material for: An MRI radiomics model for predicting a prostate-specific antigen response following abiraterone treatment in patients with metastatic castration-resistant prostate cancer
Source: Front Oncol. 2025 Jan 27;15:1491848. doi: 10.3389/fonc.2025.1491848 (PMC11807802; doi:10.3389/fonc.2025.1491848)
Supplement: Supplementary file 1 [file Table1.pdf]

Table S1:Patient demographics and baseline characteristics in two institutions

Characteristic	Institutions		p-value[2]
	1, N = 26[1]	2, N = 34[1]	
**Age**	72 ± 10	67 ± 9	0.048
**ISUP grade**			0.322
≤ 4	10 (38.46%)	9 (26.47%)	
> 4	16 (61.54%)	25 (73.53%)	
**PI-RADS**			0.305
≤ 4	6 (23.08%)	4 (11.76%)	
> 4	20 (76.92%)	30 (88.24%)	
**ADC(mm2/s)*10^{-3}**	0.54 ± 0.13	0.64 ± 0.12	0.002
**T stage**			0.003
T2-3a	12 (46.15%)	4 (11.76%)	
T3b-T4	14 (53.85%)	30 (88.24%)	
**N stage**			>0.999
N0	13 (50.00%)	17 (50.00%)	
N1	13 (50.00%)	17 (50.00%)	
**Tumor burden**			0.063
Low	9 (34.62%)	20 (58.82%)	
High	17 (65.38%)	14 (41.18%)	
**PSA**			0.511
< 100	5 (19.23%)	9 (26.47%)	
≥100	21 (80.77%)	25 (73.53%)	
**PSAD**	0.65 (0.52, 0.88)	0.35 (0.06, 0.96)	0.121
**PSAN**			>0.999
< 0.1	5 (19.23%)	6 (17.65%)	
0.1-4	12 (46.15%)	16 (47.06%)	
> 4	9 (34.62%)	12 (35.29%)	
**PSAN time(month)**	10.0 (4.3, 12.0)	5.5 (4.0, 7.0)	0.007

[1]Mean ± SD; n (%); Median (IQR)

[2]Welch Two Sample t-test; Pearson's Chi-squared test; Fisher's exact test; Wilcoxon rank sum test

Table S2 The feature numbers of MR sequence during the procedure of feature selection

Sequences	Input features	Intra-Class Correlation Coefficient (≥0.75)	Correlation analysis (P=0.05)	LASSO selection
**T2WI+DWI+ADC**	2160	1412	122	7
**T2WI**	720	535	17	5
**DWI**	720	498	21	4
**ADC**	720	379	21	4
