# Supplementary material for: An MRI radiomics model for predicting a prostate-specific antigen response following abiraterone treatment in patients with metastatic castration-resistant prostate cancer
Source: Front Oncol. 2025 Jan 27;15:1491848. doi: 10.3389/fonc.2025.1491848 (PMC11807802; doi:10.3389/fonc.2025.1491848)
Supplement: Supplementary file 2 [file DataSheet1.pdf]

Feature_name	ICC	CI95%
T2WI_wavelet_firstorder_wavelet-LLH-10Percentile	0.963	[0.86 0.99]
T2WI_wavelet_firstorder_wavelet-LLH-90Percentile	0.94	[0.78 0.98]
T2WI_wavelet_firstorder_wavelet-LLH-Energy	0.971	[0.81 0.99]
T2WI_wavelet_firstorder_wavelet-LLH-Entropy	0.972	[0.89 0.99]
T2WI_wavelet_firstorder_wavelet-LLH-InterquartileRange	0.978	[0.92 0.99]
T2WI_wavelet_firstorder_wavelet-LLH-Kurtosis	0.752	[0.28 0.93]
T2WI_wavelet_firstorder_wavelet-LLH-Maximum	0.965	[0.87 0.99]
T2WI_wavelet_firstorder_wavelet-LLH-MeanAbsoluteDeviation	0.985	[0.95 1.]
T2WI_wavelet_firstorder_wavelet-LLH-Minimum	0.942	[0.79 0.99]
T2WI_wavelet_firstorder_wavelet-LLH-Range	0.961	[0.86 0.99]
T2WI_wavelet_firstorder_wavelet-LLH-RobustMeanAbsoluteDeviat	0.98	[0.92 0.99]
T2WI_wavelet_firstorder_wavelet-LLH-RootMeanSquared	0.982	[0.93 1.]
T2WI_wavelet_firstorder_wavelet-LLH-TotalEnergy	0.98	[0.89 1.]
T2WI_wavelet_firstorder_wavelet-LLH-Uniformity	0.959	[0.85 0.99]
T2WI_wavelet_firstorder_wavelet-LLH-Variance	0.994	[0.98 1.]
T2WI_wavelet_firstorder_wavelet-LHL-10Percentile	0.996	[0.98 1.]
T2WI_wavelet_firstorder_wavelet-LHL-90Percentile	0.992	[0.97 1.]
T2WI_wavelet_firstorder_wavelet-LHL-Energy	0.871	[0.52 0.97]
T2WI_wavelet_firstorder_wavelet-LHL-Entropy	0.996	[0.98 1.]
T2WI_wavelet_firstorder_wavelet-LHL-InterquartileRange	0.995	[0.98 1.]
T2WI_wavelet_firstorder_wavelet-LHL-MeanAbsoluteDeviation	0.995	[0.98 1.]
T2WI_wavelet_firstorder_wavelet-LHL-RobustMeanAbsoluteDeviat	0.996	[0.98 1.]
T2WI_wavelet_firstorder_wavelet-LHL-RootMeanSquared	0.929	[0.76 0.98]
T2WI_wavelet_firstorder_wavelet-LHL-TotalEnergy	0.887	[0.61 0.97]
T2WI_wavelet_firstorder_wavelet-LHL-Uniformity	0.997	[0.99 1.]
T2WI_wavelet_firstorder_wavelet-LHL-Variance	0.921	[0.73 0.98]
T2WI_wavelet_firstorder_wavelet-LHH-10Percentile	0.979	[0.92 0.99]
T2WI_wavelet_firstorder_wavelet-LHH-90Percentile	0.979	[0.92 0.99]
T2WI_wavelet_firstorder_wavelet-LHH-Energy	0.941	[0.74 0.99]
T2WI_wavelet_firstorder_wavelet-LHH-Entropy	0.988	[0.95 1.]
T2WI_wavelet_firstorder_wavelet-LHH-InterquartileRange	0.979	[0.92 0.99]
T2WI_wavelet_firstorder_wavelet-LHH-MeanAbsoluteDeviation	0.985	[0.94 1.]
T2WI_wavelet_firstorder_wavelet-LHH-Mean	0.852	[0.53 0.96]
T2WI_wavelet_firstorder_wavelet-LHH-RobustMeanAbsoluteDeviat	0.978	[0.92 0.99]
T2WI_wavelet_firstorder_wavelet-LHH-RootMeanSquared	0.98	[0.93 1.]
T2WI_wavelet_firstorder_wavelet-LHH-TotalEnergy	0.925	[0.73 0.98]
T2WI_wavelet_firstorder_wavelet-LHH-Uniformity	0.988	[0.95 1.]
T2WI_wavelet_firstorder_wavelet-LHH-Variance	0.978	[0.91 0.99]
T2WI_wavelet_firstorder_wavelet-HLL-10Percentile	0.997	[0.99 1.]
T2WI_wavelet_firstorder_wavelet-HLL-90Percentile	0.998	[0.99 1.]
T2WI_wavelet_firstorder_wavelet-HLL-Energy	0.949	[0.81 0.99]
T2WI_wavelet_firstorder_wavelet-HLL-Entropy	0.99	[0.96 1.]
T2WI_wavelet_firstorder_wavelet-HLL-InterquartileRange	0.999	[1. 1.]
T2WI_wavelet_firstorder_wavelet-HLL-MeanAbsoluteDeviation	0.993	[0.97 1.]
T2WI_wavelet_firstorder_wavelet-HLL-Median	0.855	[0.48 0.96]
T2WI_wavelet_firstorder_wavelet-HLL-RobustMeanAbsoluteDeviat	0.999	[1. 1.]
T2WI_wavelet_firstorder_wavelet-HLL-RootMeanSquared	0.961	[0.85 0.99]
T2WI_wavelet_firstorder_wavelet-HLL-TotalEnergy	0.962	[0.85 0.99]
T2WI_wavelet_firstorder_wavelet-HLL-Uniformity	0.997	[0.99 1.]
T2WI_wavelet_firstorder_wavelet-HLL-Variance	0.95	[0.82 0.99]
T2WI_wavelet_firstorder_wavelet-HLH-10Percentile	0.991	[0.97 1.]

T2WI_wavelet_firstorder_wavelet-HLH-90Percentile	0.993	[0.97 1.]
T2WI_wavelet_firstorder_wavelet-HLH-Energy	0.977	[0.92 0.99]
T2WI_wavelet_firstorder_wavelet-HLH-Entropy	0.993	[0.97 1.]
T2WI_wavelet_firstorder_wavelet-HLH-InterquartileRange	0.995	[0.98 1.]
T2WI_wavelet_firstorder_wavelet-HLH-Maximum	0.921	[0.72 0.98]
T2WI_wavelet_firstorder_wavelet-HLH-MeanAbsoluteDeviation	0.994	[0.97 1.]
T2WI_wavelet_firstorder_wavelet-HLH-Median	0.987	[0.93 0.99]
T2WI_wavelet_firstorder_wavelet-HLH-Minimum	0.869	[0.46 0.97]
T2WI_wavelet_firstorder_wavelet-HLH-Range	0.951	[0.81 0.99]
T2WI_wavelet_firstorder_wavelet-HLH-RobustMeanAbsoluteDeviat	0.994	[0.97 1.]
T2WI_wavelet_firstorder_wavelet-HLH-RootMeanSquared	0.988	[0.95 1.]
T2WI_wavelet_firstorder_wavelet-HLH-TotalEnergy	0.981	[0.93 1.]
T2WI_wavelet_firstorder_wavelet-HLH-Uniformity	0.99	[0.96 1.]
T2WI_wavelet_firstorder_wavelet-HLH-Variance	0.977	[0.92 0.99]
T2WI_wavelet_firstorder_wavelet-HHL-10Percentile	0.998	[0.99 1.]
T2WI_wavelet_firstorder_wavelet-HHL-90Percentile	0.999	[0.99 1.]
T2WI_wavelet_firstorder_wavelet-HHL-Energy	0.883	[0.6 0.97]
T2WI_wavelet_firstorder_wavelet-HHL-Entropy	0.976	[0.91 0.99]
T2WI_wavelet_firstorder_wavelet-HHL-InterquartileRange	0.999	[1. 1.]
T2WI_wavelet_firstorder_wavelet-HHL-Kurtosis	0.978	[0.92 0.99]
T2WI_wavelet_firstorder_wavelet-HHL-Maximum	0.969	[0.88 0.99]
T2WI_wavelet_firstorder_wavelet-HHL-MeanAbsoluteDeviation	0.998	[0.99 1.]
T2WI_wavelet_firstorder_wavelet-HHL-Mean	0.798	[0.4 0.94]
T2WI_wavelet_firstorder_wavelet-HHL-Median	0.894	[0.64 0.97]
T2WI_wavelet_firstorder_wavelet-HHL-Minimum	0.984	[0.94 1.]
T2WI_wavelet_firstorder_wavelet-HHL-Range	0.978	[0.92 0.99]
T2WI_wavelet_firstorder_wavelet-HHL-RobustMeanAbsoluteDeviat	0.998	[0.99 1.]
T2WI_wavelet_firstorder_wavelet-HHL-RootMeanSquared	0.989	[0.96 1.]
T2WI_wavelet_firstorder_wavelet-HHL-Skewness	0.753	[0.3 0.93]
T2WI_wavelet_firstorder_wavelet-HHL-TotalEnergy	0.908	[0.68 0.98]
T2WI_wavelet_firstorder_wavelet-HHL-Uniformity	0.981	[0.92 1.]
T2WI_wavelet_firstorder_wavelet-HHL-Variance	0.99	[0.96 1.]
T2WI_wavelet_firstorder_wavelet-HHH-10Percentile	0.995	[0.98 1.]
T2WI_wavelet_firstorder_wavelet-HHH-90Percentile	0.994	[0.98 1.]
T2WI_wavelet_firstorder_wavelet-HHH-Energy	0.883	[0.6 0.97]
T2WI_wavelet_firstorder_wavelet-HHH-Entropy	0.956	[0.82 0.99]
T2WI_wavelet_firstorder_wavelet-HHH-InterquartileRange	0.992	[0.97 1.]
T2WI_wavelet_firstorder_wavelet-HHH-Kurtosis	0.777	[0.35 0.94]
T2WI_wavelet_firstorder_wavelet-HHH-Maximum	0.973	[0.9 0.99]
T2WI_wavelet_firstorder_wavelet-HHH-MeanAbsoluteDeviation	0.993	[0.98 1.]
T2WI_wavelet_firstorder_wavelet-HHH-Minimum	0.984	[0.94 1.]
T2WI_wavelet_firstorder_wavelet-HHH-Range	0.984	[0.94 1.]
T2WI_wavelet_firstorder_wavelet-HHH-RobustMeanAbsoluteDeviat	0.993	[0.97 1.]
T2WI_wavelet_firstorder_wavelet-HHH-RootMeanSquared	0.986	[0.95 1.]
T2WI_wavelet_firstorder_wavelet-HHH-TotalEnergy	0.9	[0.65 0.97]
T2WI_wavelet_firstorder_wavelet-HHH-Uniformity	0.947	[0.79 0.99]
T2WI_wavelet_firstorder_wavelet-HHH-Variance	0.978	[0.91 0.99]
T2WI_wavelet_firstorder_wavelet-LLL-10Percentile	0.997	[0.99 1.]
T2WI_wavelet_firstorder_wavelet-LLL-90Percentile	0.997	[0.99 1.]
T2WI_wavelet_firstorder_wavelet-LLL-Energy	0.989	[0.9 1.]
T2WI_wavelet_firstorder_wavelet-LLL-Entropy	0.983	[0.93 1.]
T2WI_wavelet_firstorder_wavelet-LLL-InterquartileRange	0.989	[0.96 1.]

T2WI_wavelet_firstorder_wavelet-LLL-Kurtosis	0.759	[0.3 0.93]
T2WI_wavelet_firstorder_wavelet-LLL-Maximum	0.974	[0.9 0.99]
T2WI_wavelet_firstorder_wavelet-LLL-MeanAbsoluteDeviation	0.991	[0.96 1.]
T2WI_wavelet_firstorder_wavelet-LLL-Mean	0.998	[0.99 1.]
T2WI_wavelet_firstorder_wavelet-LLL-Median	0.999	[0.99 1.]
T2WI_wavelet_firstorder_wavelet-LLL-Range	0.934	[0.75 0.98]
T2WI_wavelet_firstorder_wavelet-LLL-RobustMeanAbsoluteDeviat	0.991	[0.97 1.]
T2WI_wavelet_firstorder_wavelet-LLL-RootMeanSquared	0.998	[0.99 1.]
T2WI_wavelet_firstorder_wavelet-LLL-Skewness	0.86	[0.53 0.96]
T2WI_wavelet_firstorder_wavelet-LLL-TotalEnergy	0.993	[0.94 1.]
T2WI_wavelet_firstorder_wavelet-LLL-Uniformity	0.971	[0.89 0.99]
T2WI_wavelet_firstorder_wavelet-LLL-Variance	0.982	[0.93 1.]
T2WI_wavelet_glcm_wavelet-LLH-Autocorrelation	0.978	[0.92 0.99]
T2WI_wavelet_glcm_wavelet-LLH-JointAverage	0.943	[0.79 0.99]
T2WI_wavelet_glcm_wavelet-LLH-ClusterProminence	0.865	[0.57 0.96]
T2WI_wavelet_glcm_wavelet-LLH-ClusterShade	0.924	[0.73 0.98]
T2WI_wavelet_glcm_wavelet-LLH-ClusterTendency	0.995	[0.98 1.]
T2WI_wavelet_glcm_wavelet-LLH-Contrast	0.98	[0.93 0.99]
T2WI_wavelet_glcm_wavelet-LLH-Correlation	0.843	[0.51 0.96]
T2WI_wavelet_glcm_wavelet-LLH-DifferenceAverage	0.996	[0.98 1.]
T2WI_wavelet_glcm_wavelet-LLH-DifferenceEntropy	0.988	[0.96 1.]
T2WI_wavelet_glcm_wavelet-LLH-DifferenceVariance	0.932	[0.77 0.98]
T2WI_wavelet_glcm_wavelet-LLH-JointEnergy	0.971	[0.89 0.99]
T2WI_wavelet_glcm_wavelet-LLH-JointEntropy	0.984	[0.94 1.]
T2WI_wavelet_glcm_wavelet-LLH-InverseVariance	0.995	[0.98 1.]
T2WI_wavelet_glcm_wavelet-LLH-MaximumProbability	0.981	[0.93 1.]
T2WI_wavelet_glcm_wavelet-LLH-SumEntropy	0.97	[0.89 0.99]
T2WI_wavelet_glcm_wavelet-LLH-Idn	0.858	[0.55 0.96]
T2WI_wavelet_glcm_wavelet-LLH-Id	0.993	[0.97 1.]
T2WI_wavelet_glcm_wavelet-LLH-Idmn	0.797	[0.41 0.94]
T2WI_wavelet_glcm_wavelet-LLH-Idm	0.992	[0.97 1.]
T2WI_wavelet_glcm_wavelet-LHL-ClusterTendency	0.913	[0.7 0.98]
T2WI_wavelet_glcm_wavelet-LHL-Contrast	0.975	[0.91 0.99]
T2WI_wavelet_glcm_wavelet-LHL-Correlation	0.981	[0.93 1.]
T2WI_wavelet_glcm_wavelet-LHL-DifferenceAverage	0.996	[0.98 1.]
T2WI_wavelet_glcm_wavelet-LHL-DifferenceEntropy	0.995	[0.98 1.]
T2WI_wavelet_glcm_wavelet-LHL-DifferenceVariance	0.909	[0.68 0.98]
T2WI_wavelet_glcm_wavelet-LHL-JointEnergy	0.998	[0.99 1.]
T2WI_wavelet_glcm_wavelet-LHL-JointEntropy	0.996	[0.99 1.]
T2WI_wavelet_glcm_wavelet-LHL-InverseVariance	0.993	[0.94 1.]
T2WI_wavelet_glcm_wavelet-LHL-MaximumProbability	0.999	[0.99 1.]
T2WI_wavelet_glcm_wavelet-LHL-SumEntropy	0.994	[0.98 1.]
T2WI_wavelet_glcm_wavelet-LHL-Id	0.998	[0.99 1.]
T2WI_wavelet_glcm_wavelet-LHL-Idm	0.998	[0.99 1.]
T2WI_wavelet_glcm_wavelet-LHL-Imc2	0.813	[0.41 0.95]
T2WI_wavelet_glcm_wavelet-LHL-Imc1	0.969	[0.87 0.99]
T2WI_wavelet_glcm_wavelet-LHH-ClusterTendency	0.971	[0.9 0.99]
T2WI_wavelet_glcm_wavelet-LHH-Contrast	0.945	[0.81 0.99]
T2WI_wavelet_glcm_wavelet-LHH-Correlation	0.913	[0.7 0.98]
T2WI_wavelet_glcm_wavelet-LHH-DifferenceAverage	0.985	[0.94 1.]
T2WI_wavelet_glcm_wavelet-LHH-DifferenceEntropy	0.992	[0.97 1.]
T2WI_wavelet_glcm_wavelet-LHH-DifferenceVariance	0.87	[0.58 0.97]

T2WI_wavelet_glcm_wavelet-LHH-JointEnergy	0.994	[0.98 1.]
T2WI_wavelet_glcm_wavelet-LHH-JointEntropy	0.992	[0.97 1.]
T2WI_wavelet_glcm_wavelet-LHH-InverseVariance	0.923	[0.74 0.98]
T2WI_wavelet_glcm_wavelet-LHH-MaximumProbability	0.991	[0.97 1.]
T2WI_wavelet_glcm_wavelet-LHH-SumEntropy	0.991	[0.96 1.]
T2WI_wavelet_glcm_wavelet-LHH-Id	0.988	[0.96 1.]
T2WI_wavelet_glcm_wavelet-LHH-Idm	0.988	[0.95 1.]
T2WI_wavelet_glcm_wavelet-LHH-Imc2	0.943	[0.79 0.99]
T2WI_wavelet_glcm_wavelet-LHH-Imc1	0.963	[0.86 0.99]
T2WI_wavelet_glcm_wavelet-HLL-ClusterShade	0.915	[0.7 0.98]
T2WI_wavelet_glcm_wavelet-HLL-ClusterTendency	0.872	[0.59 0.97]
T2WI_wavelet_glcm_wavelet-HLL-Contrast	0.955	[0.84 0.99]
T2WI_wavelet_glcm_wavelet-HLL-Correlation	0.968	[0.88 0.99]
T2WI_wavelet_glcm_wavelet-HLL-DifferenceAverage	0.991	[0.96 1.]
T2WI_wavelet_glcm_wavelet-HLL-DifferenceEntropy	0.985	[0.94 1.]
T2WI_wavelet_glcm_wavelet-HLL-DifferenceVariance	0.899	[0.66 0.97]
T2WI_wavelet_glcm_wavelet-HLL-JointEnergy	0.997	[0.99 1.]
T2WI_wavelet_glcm_wavelet-HLL-JointEntropy	0.988	[0.95 1.]
T2WI_wavelet_glcm_wavelet-HLL-InverseVariance	0.995	[0.98 1.]
T2WI_wavelet_glcm_wavelet-HLL-MaximumProbability	0.993	[0.97 1.]
T2WI_wavelet_glcm_wavelet-HLL-SumEntropy	0.98	[0.92 0.99]
T2WI_wavelet_glcm_wavelet-HLL-Id	0.997	[0.99 1.]
T2WI_wavelet_glcm_wavelet-HLL-Idm	0.997	[0.99 1.]
T2WI_wavelet_glcm_wavelet-HLL-Imc2	0.868	[0.55 0.97]
T2WI_wavelet_glcm_wavelet-HLL-Imc1	0.967	[0.88 0.99]
T2WI_wavelet_glcm_wavelet-HLH-Autocorrelation	0.922	[0.7 0.98]
T2WI_wavelet_glcm_wavelet-HLH-JointAverage	0.879	[0.54 0.97]
T2WI_wavelet_glcm_wavelet-HLH-ClusterProminence	0.798	[0.4 0.94]
T2WI_wavelet_glcm_wavelet-HLH-ClusterShade	0.861	[0.53 0.96]
T2WI_wavelet_glcm_wavelet-HLH-ClusterTendency	0.956	[0.84 0.99]
T2WI_wavelet_glcm_wavelet-HLH-Contrast	0.944	[0.8 0.99]
T2WI_wavelet_glcm_wavelet-HLH-Correlation	0.942	[0.8 0.98]
T2WI_wavelet_glcm_wavelet-HLH-DifferenceAverage	0.99	[0.96 1.]
T2WI_wavelet_glcm_wavelet-HLH-DifferenceEntropy	0.995	[0.98 1.]
T2WI_wavelet_glcm_wavelet-HLH-DifferenceVariance	0.896	[0.65 0.97]
T2WI_wavelet_glcm_wavelet-HLH-JointEnergy	0.994	[0.98 1.]
T2WI_wavelet_glcm_wavelet-HLH-JointEntropy	0.995	[0.98 1.]
T2WI_wavelet_glcm_wavelet-HLH-InverseVariance	0.976	[0.91 0.99]
T2WI_wavelet_glcm_wavelet-HLH-MaximumProbability	0.998	[0.99 1.]
T2WI_wavelet_glcm_wavelet-HLH-SumEntropy	0.995	[0.98 1.]
T2WI_wavelet_glcm_wavelet-HLH-Id	0.993	[0.98 1.]
T2WI_wavelet_glcm_wavelet-HLH-Idm	0.994	[0.98 1.]
T2WI_wavelet_glcm_wavelet-HLH-Idmn	0.993	[0.98 1.]
T2WI_wavelet_glcm_wavelet-HLH-Imc2	0.969	[0.88 0.99]
T2WI_wavelet_glcm_wavelet-HLH-Imc1	0.971	[0.89 0.99]
T2WI_wavelet_glcm_wavelet-HHL-Autocorrelation	0.99	[0.96 1.]
T2WI_wavelet_glcm_wavelet-HHL-JointAverage	0.977	[0.91 0.99]
T2WI_wavelet_glcm_wavelet-HHL-ClusterTendency	0.767	[0.33 0.94]
T2WI_wavelet_glcm_wavelet-HHL-Contrast	0.958	[0.84 0.99]
T2WI_wavelet_glcm_wavelet-HHL-Correlation	0.997	[0.99 1.]
T2WI_wavelet_glcm_wavelet-HHL-DifferenceAverage	0.99	[0.96 1.]
T2WI_wavelet_glcm_wavelet-HHL-DifferenceEntropy	0.957	[0.84 0.99]

T2WI_wavelet_glcm_wavelet-HHL-DifferenceVariance	0.867	[0.57 0.96]
T2WI_wavelet_glcm_wavelet-HHL-JointEnergy	0.977	[0.9 0.99]
T2WI_wavelet_glcm_wavelet-HHL-JointEntropy	0.964	[0.86 0.99]
T2WI_wavelet_glcm_wavelet-HHL-InverseVariance	1	[1. 1.]
T2WI_wavelet_glcm_wavelet-HHL-MaximumProbability	0.993	[0.97 1.]
T2WI_wavelet_glcm_wavelet-HHL-SumEntropy	0.91	[0.67 0.98]
T2WI_wavelet_glcm_wavelet-HHL-Idn	0.865	[0.57 0.96]
T2WI_wavelet_glcm_wavelet-HHL-Id	0.995	[0.98 1.]
T2WI_wavelet_glcm_wavelet-HHL-Idmn	0.751	[0.31 0.93]
T2WI_wavelet_glcm_wavelet-HHL-Idm	0.995	[0.98 1.]
T2WI_wavelet_glcm_wavelet-HHH-Autocorrelation	0.998	[0.99 1.]
T2WI_wavelet_glcm_wavelet-HHH-JointAverage	0.991	[0.96 1.]
T2WI_wavelet_glcm_wavelet-HHH-ClusterTendency	0.868	[0.54 0.97]
T2WI_wavelet_glcm_wavelet-HHH-Contrast	0.863	[0.53 0.96]
T2WI_wavelet_glcm_wavelet-HHH-Correlation	0.902	[0.66 0.97]
T2WI_wavelet_glcm_wavelet-HHH-DifferenceAverage	0.913	[0.68 0.98]
T2WI_wavelet_glcm_wavelet-HHH-DifferenceEntropy	0.938	[0.75 0.98]
T2WI_wavelet_glcm_wavelet-HHH-DifferenceVariance	0.811	[0.42 0.95]
T2WI_wavelet_glcm_wavelet-HHH-JointEnergy	0.938	[0.76 0.98]
T2WI_wavelet_glcm_wavelet-HHH-JointEntropy	0.944	[0.77 0.99]
T2WI_wavelet_glcm_wavelet-HHH-InverseVariance	0.892	[0.62 0.97]
T2WI_wavelet_glcm_wavelet-HHH-MaximumProbability	0.98	[0.91 1.]
T2WI_wavelet_glcm_wavelet-HHH-SumEntropy	0.941	[0.76 0.99]
T2WI_wavelet_glcm_wavelet-HHH-Idn	0.877	[0.6 0.97]
T2WI_wavelet_glcm_wavelet-HHH-Id	0.922	[0.69 0.98]
T2WI_wavelet_glcm_wavelet-HHH-Idmn	0.762	[0.33 0.93]
T2WI_wavelet_glcm_wavelet-HHH-Idm	0.923	[0.7 0.98]
T2WI_wavelet_glcm_wavelet-LLL-Autocorrelation	0.982	[0.93 1.]
T2WI_wavelet_glcm_wavelet-LLL-JointAverage	0.932	[0.75 0.98]
T2WI_wavelet_glcm_wavelet-LLL-ClusterProminence	0.859	[0.55 0.96]
T2WI_wavelet_glcm_wavelet-LLL-ClusterShade	0.94	[0.79 0.98]
T2WI_wavelet_glcm_wavelet-LLL-ClusterTendency	0.98	[0.93 1.]
T2WI_wavelet_glcm_wavelet-LLL-Contrast	0.969	[0.89 0.99]
T2WI_wavelet_glcm_wavelet-LLL-Correlation	0.962	[0.84 0.99]
T2WI_wavelet_glcm_wavelet-LLL-DifferenceAverage	0.991	[0.97 1.]
T2WI_wavelet_glcm_wavelet-LLL-DifferenceEntropy	0.988	[0.96 1.]
T2WI_wavelet_glcm_wavelet-LLL-DifferenceVariance	0.907	[0.69 0.98]
T2WI_wavelet_glcm_wavelet-LLL-JointEnergy	0.924	[0.74 0.98]
T2WI_wavelet_glcm_wavelet-LLL-JointEntropy	0.983	[0.94 1.]
T2WI_wavelet_glcm_wavelet-LLL-InverseVariance	0.981	[0.93 1.]
T2WI_wavelet_glcm_wavelet-LLL-MaximumProbability	0.9	[0.66 0.97]
T2WI_wavelet_glcm_wavelet-LLL-SumEntropy	0.983	[0.93 1.]
T2WI_wavelet_glcm_wavelet-LLL-Id	0.984	[0.94 1.]
T2WI_wavelet_glcm_wavelet-LLL-Idm	0.982	[0.93 1.]
T2WI_wavelet_glcm_wavelet-LLL-Imc2	0.96	[0.85 0.99]
T2WI_wavelet_glcm_wavelet-LLL-Imc1	0.971	[0.9 0.99]
T2WI_wavelet_glrlm_wavelet-LLH-GrayLevelNonUniformity	0.949	[0.74 0.99]
T2WI_wavelet_glrlm_wavelet-LLH-GrayLevelNonUniformityNormali	0.954	[0.83 0.99]
T2WI_wavelet_glrlm_wavelet-LLH-GrayLevelVariance	0.993	[0.97 1.]
T2WI_wavelet_glrlm_wavelet-LLH-HighGrayLevelRunEmphasis	0.978	[0.92 0.99]
T2WI_wavelet_glrlm_wavelet-LLH-LongRunEmphasis	0.964	[0.87 0.99]
T2WI_wavelet_glrlm_wavelet-LLH-LongRunHighGrayLevelEmphasis	0.943	[0.8 0.99]

T2WI_wavelet_glrlm_wavelet-LLH-RunEntropy	0.955 [0.84 0.99]
T2WI_wavelet_glrlm_wavelet-LLH-RunLengthNonUniformity	0.9 [0.4 0.98]
T2WI_wavelet_glrlm_wavelet-LLH-RunLengthNonUniformityNormali	0.958 [0.84 0.99]
T2WI_wavelet_glrlm_wavelet-LLH-RunPercentage	0.963 [0.86 0.99]
T2WI_wavelet_glrlm_wavelet-LLH-RunVariance	0.97 [0.89 0.99]
T2WI_wavelet_glrlm_wavelet-LLH-ShortRunEmphasis	0.956 [0.84 0.99]
T2WI_wavelet_glrlm_wavelet-LLH-ShortRunHighGrayLevelEmphasis	0.981 [0.93 1.]
T2WI_wavelet_glrlm_wavelet-LHL-GrayLevelNonUniformity	0.906 [0.47 0.98]
T2WI_wavelet_glrlm_wavelet-LHL-GrayLevelNonUniformityNormali	0.995 [0.98 1.]
T2WI_wavelet_glrlm_wavelet-LHL-GrayLevelVariance	0.83 [0.48 0.95]
T2WI_wavelet_glrlm_wavelet-LHL-LongRunEmphasis	0.995 [0.98 1.]
T2WI_wavelet_glrlm_wavelet-LHL-RunEntropy	0.908 [0.68 0.98]
T2WI_wavelet_glrlm_wavelet-LHL-RunLengthNonUniformity	0.78 [0.12 0.95]
T2WI_wavelet_glrlm_wavelet-LHL-RunLengthNonUniformityNormali	0.972 [0.89 0.99]
T2WI_wavelet_glrlm_wavelet-LHL-RunPercentage	0.983 [0.93 1.]
T2WI_wavelet_glrlm_wavelet-LHL-RunVariance	0.998 [0.99 1.]
T2WI_wavelet_glrlm_wavelet-LHL-ShortRunEmphasis	0.982 [0.93 1.]
T2WI_wavelet_glrlm_wavelet-LHH-GrayLevelNonUniformity	0.932 [0.58 0.98]
T2WI_wavelet_glrlm_wavelet-LHH-GrayLevelNonUniformityNormali	0.987 [0.95 1.]
T2WI_wavelet_glrlm_wavelet-LHH-GrayLevelVariance	0.969 [0.88 0.99]
T2WI_wavelet_glrlm_wavelet-LHH-LongRunEmphasis	0.992 [0.97 1.]
T2WI_wavelet_glrlm_wavelet-LHH-RunEntropy	0.879 [0.61 0.97]
T2WI_wavelet_glrlm_wavelet-LHH-RunLengthNonUniformity	0.846 [0.22 0.97]
T2WI_wavelet_glrlm_wavelet-LHH-RunLengthNonUniformityNormali	0.944 [0.79 0.99]
T2WI_wavelet_glrlm_wavelet-LHH-RunPercentage	0.959 [0.85 0.99]
T2WI_wavelet_glrlm_wavelet-LHH-RunVariance	0.995 [0.98 1.]
T2WI_wavelet_glrlm_wavelet-LHH-ShortRunEmphasis	0.971 [0.89 0.99]
T2WI_wavelet_glrlm_wavelet-HLL-GrayLevelNonUniformity	0.902 [0.44 0.98]
T2WI_wavelet_glrlm_wavelet-HLL-GrayLevelNonUniformityNormali	0.991 [0.97 1.]
T2WI_wavelet_glrlm_wavelet-HLL-GrayLevelVariance	0.916 [0.7 0.98]
T2WI_wavelet_glrlm_wavelet-HLL-LongRunEmphasis	0.994 [0.98 1.]
T2WI_wavelet_glrlm_wavelet-HLL-RunEntropy	0.787 [0.35 0.94]
T2WI_wavelet_glrlm_wavelet-HLL-RunLengthNonUniformity	0.843 [0.23 0.96]
T2WI_wavelet_glrlm_wavelet-HLL-RunLengthNonUniformityNormali	0.969 [0.88 0.99]
T2WI_wavelet_glrlm_wavelet-HLL-RunPercentage	0.975 [0.9 0.99]
T2WI_wavelet_glrlm_wavelet-HLL-RunVariance	0.996 [0.98 1.]
T2WI_wavelet_glrlm_wavelet-HLL-ShortRunEmphasis	0.981 [0.92 1.]
T2WI_wavelet_glrlm_wavelet-HLH-GrayLevelNonUniformity	0.928 [0.57 0.98]
T2WI_wavelet_glrlm_wavelet-HLH-GrayLevelNonUniformityNormali	0.993 [0.97 1.]
T2WI_wavelet_glrlm_wavelet-HLH-GrayLevelVariance	0.97 [0.89 0.99]
T2WI_wavelet_glrlm_wavelet-HLH-HighGrayLevelRunEmphasis	0.922 [0.7 0.98]
T2WI_wavelet_glrlm_wavelet-HLH-LongRunEmphasis	0.988 [0.95 1.]
T2WI_wavelet_glrlm_wavelet-HLH-LongRunHighGrayLevelEmphasis	0.801 [0.39 0.95]
T2WI_wavelet_glrlm_wavelet-HLH-RunEntropy	0.887 [0.63 0.97]
T2WI_wavelet_glrlm_wavelet-HLH-RunLengthNonUniformity	0.865 [0.28 0.97]
T2WI_wavelet_glrlm_wavelet-HLH-RunLengthNonUniformityNormali	0.907 [0.68 0.98]
T2WI_wavelet_glrlm_wavelet-HLH-RunPercentage	0.925 [0.73 0.98]
T2WI_wavelet_glrlm_wavelet-HLH-RunVariance	0.986 [0.95 1.]
T2WI_wavelet_glrlm_wavelet-HLH-ShortRunEmphasis	0.951 [0.82 0.99]
T2WI_wavelet_glrlm_wavelet-HLH-ShortRunHighGrayLevelEmphasis	0.946 [0.78 0.99]
T2WI_wavelet_glrlm_wavelet-HHL-GrayLevelNonUniformity	0.889 [0.38 0.98]
T2WI_wavelet_glrlm_wavelet-HHL-GrayLevelNonUniformityNormali	0.98 [0.92 0.99]

T2WI_wavelet_glrlm_wavelet-HHL-GrayLevelVariance	0.931	[0.76 0.98]
T2WI_wavelet_glrlm_wavelet-HHL-HighGrayLevelRunEmphasis	0.99	[0.96 1.]
T2WI_wavelet_glrlm_wavelet-HHL-LongRunEmphasis	0.974	[0.9 0.99]
T2WI_wavelet_glrlm_wavelet-HHL-LongRunHighGrayLevelEmphasis	0.994	[0.98 1.]
T2WI_wavelet_glrlm_wavelet-HHL-LongRunLowGrayLevelEmphasis	0.886	[0.63 0.97]
T2WI_wavelet_glrlm_wavelet-HHL-LowGrayLevelRunEmphasis	0.786	[0.38 0.94]
T2WI_wavelet_glrlm_wavelet-HHL-RunEntropy	0.771	[0.3 0.94]
T2WI_wavelet_glrlm_wavelet-HHL-RunLengthNonUniformity	0.817	[0.19 0.96]
T2WI_wavelet_glrlm_wavelet-HHL-RunLengthNonUniformityNormali	0.873	[0.58 0.97]
T2WI_wavelet_glrlm_wavelet-HHL-RunPercentage	0.899	[0.65 0.97]
T2WI_wavelet_glrlm_wavelet-HHL-RunVariance	0.979	[0.92 0.99]
T2WI_wavelet_glrlm_wavelet-HHL-ShortRunEmphasis	0.92	[0.71 0.98]
T2WI_wavelet_glrlm_wavelet-HHL-ShortRunHighGrayLevelEmphasis	0.987	[0.95 1.]
T2WI_wavelet_glrlm_wavelet-HHH-GrayLevelNonUniformity	0.902	[0.43 0.98]
T2WI_wavelet_glrlm_wavelet-HHH-GrayLevelNonUniformityNormali	0.97	[0.88 0.99]
T2WI_wavelet_glrlm_wavelet-HHH-GrayLevelVariance	0.921	[0.71 0.98]
T2WI_wavelet_glrlm_wavelet-HHH-HighGrayLevelRunEmphasis	0.998	[0.99 1.]
T2WI_wavelet_glrlm_wavelet-HHH-LongRunEmphasis	0.923	[0.73 0.98]
T2WI_wavelet_glrlm_wavelet-HHH-LongRunHighGrayLevelEmphasis	0.998	[0.99 1.]
T2WI_wavelet_glrlm_wavelet-HHH-LongRunLowGrayLevelEmphasis	0.989	[0.96 1.]
T2WI_wavelet_glrlm_wavelet-HHH-LowGrayLevelRunEmphasis	0.861	[0.56 0.96]
T2WI_wavelet_glrlm_wavelet-HHH-RunLengthNonUniformity	0.871	[0.32 0.97]
T2WI_wavelet_glrlm_wavelet-HHH-RunVariance	0.933	[0.77 0.98]
T2WI_wavelet_glrlm_wavelet-HHH-ShortRunEmphasis	0.782	[0.34 0.94]
T2WI_wavelet_glrlm_wavelet-HHH-ShortRunHighGrayLevelEmphasis	0.998	[0.99 1.]
T2WI_wavelet_glrlm_wavelet-LLL-GrayLevelNonUniformity	0.931	[0.55 0.98]
T2WI_wavelet_glrlm_wavelet-LLL-GrayLevelNonUniformityNormali	0.974	[0.9 0.99]
T2WI_wavelet_glrlm_wavelet-LLL-GrayLevelVariance	0.979	[0.92 0.99]
T2WI_wavelet_glrlm_wavelet-LLL-HighGrayLevelRunEmphasis	0.983	[0.93 1.]
T2WI_wavelet_glrlm_wavelet-LLL-LongRunEmphasis	0.973	[0.89 0.99]
T2WI_wavelet_glrlm_wavelet-LLL-LongRunHighGrayLevelEmphasis	0.972	[0.89 0.99]
T2WI_wavelet_glrlm_wavelet-LLL-RunEntropy	0.958	[0.84 0.99]
T2WI_wavelet_glrlm_wavelet-LLL-RunLengthNonUniformity	0.902	[0.44 0.98]
T2WI_wavelet_glrlm_wavelet-LLL-RunLengthNonUniformityNormali	0.949	[0.81 0.99]
T2WI_wavelet_glrlm_wavelet-LLL-RunPercentage	0.96	[0.85 0.99]
T2WI_wavelet_glrlm_wavelet-LLL-RunVariance	0.979	[0.92 0.99]
T2WI_wavelet_glrlm_wavelet-LLL-ShortRunEmphasis	0.957	[0.84 0.99]
T2WI_wavelet_glrlm_wavelet-LLL-ShortRunHighGrayLevelEmphasis	0.984	[0.94 1.]
T2WI_wavelet_glszm_wavelet-LLH-GrayLevelNonUniformity	0.881	[0.24 0.97]
T2WI_wavelet_glszm_wavelet-LLH-GrayLevelNonUniformityNormali	0.855	[0.53 0.96]
T2WI_wavelet_glszm_wavelet-LLH-GrayLevelVariance	0.966	[0.87 0.99]
T2WI_wavelet_glszm_wavelet-LLH-HighGrayLevelZoneEmphasis	0.977	[0.92 0.99]
T2WI_wavelet_glszm_wavelet-LLH-LargeAreaEmphasis	0.98	[0.93 0.99]
T2WI_wavelet_glszm_wavelet-LLH-LargeAreaHighGrayLevelEmphasi	0.886	[0.62 0.97]
T2WI_wavelet_glszm_wavelet-LLH-LargeAreaLowGrayLevelEmphasis	0.884	[0.62 0.97]
T2WI_wavelet_glszm_wavelet-LLH-SizeZoneNonUniformity	0.948	[0.52 0.99]
T2WI_wavelet_glszm_wavelet-LLH-SizeZoneNonUniformityNormaliz	0.931	[0.76 0.98]
T2WI_wavelet_glszm_wavelet-LLH-SmallAreaEmphasis	0.919	[0.72 0.98]
T2WI_wavelet_glszm_wavelet-LLH-SmallAreaHighGrayLevelEmphasi	0.983	[0.94 1.]
T2WI_wavelet_glszm_wavelet-LLH-ZonePercentage	0.949	[0.81 0.99]
T2WI_wavelet_glszm_wavelet-LLH-ZoneVariance	0.98	[0.93 0.99]
T2WI_wavelet_glszm_wavelet-LHL-GrayLevelNonUniformityNormali	0.939	[0.79 0.98]

T2WI_wavelet_glszm_wavelet-LHL-LargeAreaEmphasis	0.988	[0.96 1.]
T2WI_wavelet_glszm_wavelet-LHL-SizeZoneNonUniformityNormaliz	0.979	[0.92 0.99]
T2WI_wavelet_glszm_wavelet-LHL-SmallAreaEmphasis	0.975	[0.91 0.99]
T2WI_wavelet_glszm_wavelet-LHL-ZoneEntropy	0.933	[0.77 0.98]
T2WI_wavelet_glszm_wavelet-LHL-ZonePercentage	0.822	[0.45 0.95]
T2WI_wavelet_glszm_wavelet-LHL-ZoneVariance	0.989	[0.96 1.]
T2WI_wavelet_glszm_wavelet-LHH-GrayLevelNonUniformity	0.795	[0.1 0.95]
T2WI_wavelet_glszm_wavelet-LHH-GrayLevelNonUniformityNormali	0.91	[0.69 0.98]
T2WI_wavelet_glszm_wavelet-LHH-GrayLevelVariance	0.752	[0.27 0.93]
T2WI_wavelet_glszm_wavelet-LHH-LargeAreaEmphasis	0.972	[0.88 0.99]
T2WI_wavelet_glszm_wavelet-LHH-SizeZoneNonUniformity	0.861	[0.44 0.97]
T2WI_wavelet_glszm_wavelet-LHH-SizeZoneNonUniformityNormaliz	0.994	[0.98 1.]
T2WI_wavelet_glszm_wavelet-LHH-SmallAreaEmphasis	0.99	[0.96 1.]
T2WI_wavelet_glszm_wavelet-LHH-ZoneEntropy	0.927	[0.73 0.98]
T2WI_wavelet_glszm_wavelet-LHH-ZoneVariance	0.972	[0.88 0.99]
T2WI_wavelet_glszm_wavelet-HLL-GrayLevelNonUniformityNormali	0.943	[0.79 0.99]
T2WI_wavelet_glszm_wavelet-HLL-LargeAreaEmphasis	0.983	[0.93 1.]
T2WI_wavelet_glszm_wavelet-HLL-SizeZoneNonUniformity	0.95	[0.61 0.99]
T2WI_wavelet_glszm_wavelet-HLL-SizeZoneNonUniformityNormaliz	0.982	[0.93 1.]
T2WI_wavelet_glszm_wavelet-HLL-SmallAreaEmphasis	0.967	[0.87 0.99]
T2WI_wavelet_glszm_wavelet-HLL-ZoneEntropy	0.912	[0.69 0.98]
T2WI_wavelet_glszm_wavelet-HLL-ZonePercentage	0.962	[0.86 0.99]
T2WI_wavelet_glszm_wavelet-HLL-ZoneVariance	0.983	[0.94 1.]
T2WI_wavelet_glszm_wavelet-HLH-GrayLevelNonUniformityNormali	0.947	[0.81 0.99]
T2WI_wavelet_glszm_wavelet-HLH-GrayLevelVariance	0.903	[0.67 0.97]
T2WI_wavelet_glszm_wavelet-HLH-HighGrayLevelZoneEmphasis	0.925	[0.72 0.98]
T2WI_wavelet_glszm_wavelet-HLH-LargeAreaEmphasis	0.978	[0.92 0.99]
T2WI_wavelet_glszm_wavelet-HLH-LargeAreaHighGrayLevelEmphasi	0.795	[0.4 0.94]
T2WI_wavelet_glszm_wavelet-HLH-LargeAreaLowGrayLevelEmphasis	0.77	[0.3 0.94]
T2WI_wavelet_glszm_wavelet-HLH-SizeZoneNonUniformity	0.947	[0.66 0.99]
T2WI_wavelet_glszm_wavelet-HLH-SizeZoneNonUniformityNormaliz	0.927	[0.75 0.98]
T2WI_wavelet_glszm_wavelet-HLH-SmallAreaEmphasis	0.948	[0.81 0.99]
T2WI_wavelet_glszm_wavelet-HLH-SmallAreaHighGrayLevelEmphasi	0.952	[0.81 0.99]
T2WI_wavelet_glszm_wavelet-HLH-ZoneEntropy	0.954	[0.83 0.99]
T2WI_wavelet_glszm_wavelet-HLH-ZonePercentage	0.928	[0.75 0.98]
T2WI_wavelet_glszm_wavelet-HLH-ZoneVariance	0.978	[0.92 0.99]
T2WI_wavelet_glszm_wavelet-HHL-GrayLevelNonUniformity	0.801	[0.41 0.95]
T2WI_wavelet_glszm_wavelet-HHL-GrayLevelVariance	0.785	[0.38 0.94]
T2WI_wavelet_glszm_wavelet-HHL-HighGrayLevelZoneEmphasis	0.989	[0.96 1.]
T2WI_wavelet_glszm_wavelet-HHL-LargeAreaEmphasis	0.966	[0.88 0.99]
T2WI_wavelet_glszm_wavelet-HHL-LargeAreaHighGrayLevelEmphasi	0.975	[0.91 0.99]
T2WI_wavelet_glszm_wavelet-HHL-LargeAreaLowGrayLevelEmphasis	0.926	[0.74 0.98]
T2WI_wavelet_glszm_wavelet-HHL-SizeZoneNonUniformityNormaliz	0.763	[0.3 0.94]
T2WI_wavelet_glszm_wavelet-HHL-SmallAreaEmphasis	0.809	[0.41 0.95]
T2WI_wavelet_glszm_wavelet-HHL-SmallAreaHighGrayLevelEmphasi	0.983	[0.93 1.]
T2WI_wavelet_glszm_wavelet-HHL-ZonePercentage	0.946	[0.81 0.99]
T2WI_wavelet_glszm_wavelet-HHL-ZoneVariance	0.97	[0.89 0.99]
T2WI_wavelet_glszm_wavelet-HHH-GrayLevelNonUniformity	0.837	[0.5 0.96]
T2WI_wavelet_glszm_wavelet-HHH-GrayLevelVariance	0.756	[0.31 0.93]
T2WI_wavelet_glszm_wavelet-HHH-HighGrayLevelZoneEmphasis	0.989	[0.96 1.]
T2WI_wavelet_glszm_wavelet-HHH-LargeAreaEmphasis	0.909	[0.64 0.98]
T2WI_wavelet_glszm_wavelet-HHH-LargeAreaHighGrayLevelEmphasi	0.897	[0.63 0.97]

T2WI_wavelet_glszm_wavelet-HHH-LargeAreaLowGrayLevelEmphasis	0.913 [0.66 0.98]
T2WI_wavelet_glszm_wavelet-HHH-SizeZoneNonUniformity	0.86 [0.56 0.96]
T2WI_wavelet_glszm_wavelet-HHH-SizeZoneNonUniformityNormaliz	0.756 [0.31 0.93]
T2WI_wavelet_glszm_wavelet-HHH-SmallAreaEmphasis	0.807 [0.4 0.95]
T2WI_wavelet_glszm_wavelet-HHH-SmallAreaHighGrayLevelEmphasi	0.993 [0.97 1.]
T2WI_wavelet_glszm_wavelet-HHH-ZonePercentage	0.9 [0.67 0.97]
T2WI_wavelet_glszm_wavelet-HHH-ZoneVariance	0.927 [0.7 0.98]
T2WI_wavelet_glszm_wavelet-LLL-GrayLevelNonUniformity	0.853 [0.27 0.97]
T2WI_wavelet_glszm_wavelet-LLL-GrayLevelNonUniformityNormali	0.914 [0.7 0.98]
T2WI_wavelet_glszm_wavelet-LLL-GrayLevelVariance	0.9 [0.67 0.97]
T2WI_wavelet_glszm_wavelet-LLL-HighGrayLevelZoneEmphasis	0.979 [0.92 0.99]
T2WI_wavelet_glszm_wavelet-LLL-LargeAreaEmphasis	0.961 [0.86 0.99]
T2WI_wavelet_glszm_wavelet-LLL-LargeAreaHighGrayLevelEmphasi	0.972 [0.88 0.99]
T2WI_wavelet_glszm_wavelet-LLL-LargeAreaLowGrayLevelEmphasis	0.811 [0.43 0.95]
T2WI_wavelet_glszm_wavelet-LLL-SizeZoneNonUniformity	0.941 [0.58 0.99]
T2WI_wavelet_glszm_wavelet-LLL-SizeZoneNonUniformityNormaliz	0.962 [0.82 0.99]
T2WI_wavelet_glszm_wavelet-LLL-SmallAreaEmphasis	0.955 [0.76 0.99]
T2WI_wavelet_glszm_wavelet-LLL-SmallAreaHighGrayLevelEmphasi	0.984 [0.94 1.]
T2WI_wavelet_glszm_wavelet-LLL-ZonePercentage	0.943 [0.79 0.99]
T2WI_wavelet_glszm_wavelet-LLL-ZoneVariance	0.961 [0.86 0.99]
T2WI_wavelet_gldm_wavelet-LLH-DependenceEntropy	0.897 [0.66 0.97]
T2WI_wavelet_gldm_wavelet-LLH-DependenceNonUniformity	0.931 [0.43 0.99]
T2WI_wavelet_gldm_wavelet-LLH-DependenceNonUniformityNormali	0.97 [0.89 0.99]
T2WI_wavelet_gldm_wavelet-LLH-DependenceVariance	0.986 [0.95 1.]
T2WI_wavelet_gldm_wavelet-LLH-GrayLevelNonUniformity	0.958 [0.79 0.99]
T2WI_wavelet_gldm_wavelet-LLH-GrayLevelVariance	0.994 [0.98 1.]
T2WI_wavelet_gldm_wavelet-LLH-HighGrayLevelEmphasis	0.978 [0.92 0.99]
T2WI_wavelet_gldm_wavelet-LLH-LargeDependenceEmphasis	0.968 [0.88 0.99]
T2WI_wavelet_gldm_wavelet-LLH-LargeDependenceHighGrayLevelEm	0.789 [0.38 0.94]
T2WI_wavelet_gldm_wavelet-LLH-SmallDependenceEmphasis	0.957 [0.84 0.99]
T2WI_wavelet_gldm_wavelet-LLH-SmallDependenceHighGrayLevelEm	0.996 [0.98 1.]
T2WI_wavelet_gldm_wavelet-LHL-DependenceEntropy	0.897 [0.56 0.97]
T2WI_wavelet_gldm_wavelet-LHL-DependenceNonUniformity	0.928 [0.55 0.98]
T2WI_wavelet_gldm_wavelet-LHL-DependenceNonUniformityNormali	0.796 [0.4 0.94]
T2WI_wavelet_gldm_wavelet-LHL-DependenceVariance	0.935 [0.57 0.99]
T2WI_wavelet_gldm_wavelet-LHL-GrayLevelNonUniformity	0.934 [0.62 0.99]
T2WI_wavelet_gldm_wavelet-LHL-GrayLevelVariance	0.911 [0.7 0.98]
T2WI_wavelet_gldm_wavelet-LHL-LargeDependenceEmphasis	0.992 [0.97 1.]
T2WI_wavelet_gldm_wavelet-LHL-SmallDependenceEmphasis	0.861 [0.55 0.96]
T2WI_wavelet_gldm_wavelet-LHH-DependenceEntropy	0.929 [0.63 0.98]
T2WI_wavelet_gldm_wavelet-LHH-DependenceNonUniformity	0.947 [0.65 0.99]
T2WI_wavelet_gldm_wavelet-LHH-DependenceVariance	0.833 [0.36 0.96]
T2WI_wavelet_gldm_wavelet-LHH-GrayLevelNonUniformity	0.939 [0.65 0.99]
T2WI_wavelet_gldm_wavelet-LHH-GrayLevelVariance	0.976 [0.91 0.99]
T2WI_wavelet_gldm_wavelet-LHH-LargeDependenceEmphasis	0.977 [0.91 0.99]
T2WI_wavelet_gldm_wavelet-LHH-SmallDependenceEmphasis	0.819 [0.45 0.95]
T2WI_wavelet_gldm_wavelet-HLL-DependenceEntropy	0.923 [0.72 0.98]
T2WI_wavelet_gldm_wavelet-HLL-DependenceNonUniformity	0.928 [0.57 0.98]
T2WI_wavelet_gldm_wavelet-HLL-DependenceNonUniformityNormali	0.929 [0.61 0.98]
T2WI_wavelet_gldm_wavelet-HLL-DependenceVariance	0.94 [0.62 0.99]
T2WI_wavelet_gldm_wavelet-HLL-GrayLevelNonUniformity	0.935 [0.6 0.99]
T2WI_wavelet_gldm_wavelet-HLL-GrayLevelVariance	0.945 [0.8 0.99]

T2WI_wavelet_gldm_wavelet-HLL-LargeDependenceEmphasis	0.983	[0.94 1.]
T2WI_wavelet_gldm_wavelet-HLL-SmallDependenceEmphasis	0.962	[0.86 0.99]
T2WI_wavelet_gldm_wavelet-HLL-SmallDependenceHighGrayLevelEm	0.843	[0.5 0.96]
T2WI_wavelet_gldm_wavelet-HLH-DependenceEntropy	0.976	[0.89 0.99]
T2WI_wavelet_gldm_wavelet-HLH-DependenceNonUniformity	0.945	[0.66 0.99]
T2WI_wavelet_gldm_wavelet-HLH-DependenceNonUniformityNormali	0.889	[0.3 0.98]
T2WI_wavelet_gldm_wavelet-HLH-DependenceVariance	0.873	[0.37 0.97]
T2WI_wavelet_gldm_wavelet-HLH-GrayLevelNonUniformity	0.937	[0.63 0.99]
T2WI_wavelet_gldm_wavelet-HLH-GrayLevelVariance	0.975	[0.91 0.99]
T2WI_wavelet_gldm_wavelet-HLH-HighGrayLevelEmphasis	0.922	[0.7 0.98]
T2WI_wavelet_gldm_wavelet-HLH-LargeDependenceEmphasis	0.94	[0.79 0.98]
T2WI_wavelet_gldm_wavelet-HLH-LargeDependenceHighGrayLevelEm	0.824	[0.43 0.95]
T2WI_wavelet_gldm_wavelet-HLH-SmallDependenceEmphasis	0.938	[0.78 0.98]
T2WI_wavelet_gldm_wavelet-HLH-SmallDependenceHighGrayLevelEm	0.99	[0.96 1.]
T2WI_wavelet_gldm_wavelet-HHL-DependenceEntropy	0.915	[0.6 0.98]
T2WI_wavelet_gldm_wavelet-HHL-DependenceNonUniformity	0.931	[0.57 0.98]
T2WI_wavelet_gldm_wavelet-HHL-DependenceNonUniformityNormali	0.953	[0.7 0.99]
T2WI_wavelet_gldm_wavelet-HHL-DependenceVariance	0.83	[0.28 0.96]
T2WI_wavelet_gldm_wavelet-HHL-GrayLevelNonUniformity	0.914	[0.49 0.98]
T2WI_wavelet_gldm_wavelet-HHL-GrayLevelVariance	0.949	[0.81 0.99]
T2WI_wavelet_gldm_wavelet-HHL-HighGrayLevelEmphasis	0.99	[0.96 1.]
T2WI_wavelet_gldm_wavelet-HHL-LargeDependenceEmphasis	0.939	[0.78 0.98]
T2WI_wavelet_gldm_wavelet-HHL-LargeDependenceHighGrayLevelEm	0.994	[0.97 1.]
T2WI_wavelet_gldm_wavelet-HHL-LargeDependenceLowGrayLevelEmp	0.894	[0.65 0.97]
T2WI_wavelet_gldm_wavelet-HHL-LowGrayLevelEmphasis	0.793	[0.4 0.94]
T2WI_wavelet_gldm_wavelet-HHL-SmallDependenceEmphasis	0.877	[0.6 0.97]
T2WI_wavelet_gldm_wavelet-HHL-SmallDependenceHighGrayLevelEm	0.972	[0.9 0.99]
T2WI_wavelet_gldm_wavelet-HHH-DependenceEntropy	0.814	[0.44 0.95]
T2WI_wavelet_gldm_wavelet-HHH-DependenceNonUniformity	0.943	[0.64 0.99]
T2WI_wavelet_gldm_wavelet-HHH-DependenceNonUniformityNormali	0.877	[0.51 0.97]
T2WI_wavelet_gldm_wavelet-HHH-DependenceVariance	0.821	[0.32 0.96]
T2WI_wavelet_gldm_wavelet-HHH-GrayLevelNonUniformity	0.914	[0.49 0.98]
T2WI_wavelet_gldm_wavelet-HHH-GrayLevelVariance	0.909	[0.66 0.98]
T2WI_wavelet_gldm_wavelet-HHH-HighGrayLevelEmphasis	0.998	[0.99 1.]
T2WI_wavelet_gldm_wavelet-HHH-LargeDependenceEmphasis	0.787	[0.36 0.94]
T2WI_wavelet_gldm_wavelet-HHH-LargeDependenceHighGrayLevelEm	0.997	[0.99 1.]
T2WI_wavelet_gldm_wavelet-HHH-LargeDependenceLowGrayLevelEmp	0.997	[0.99 1.]
T2WI_wavelet_gldm_wavelet-HHH-LowGrayLevelEmphasis	0.874	[0.59 0.97]
T2WI_wavelet_gldm_wavelet-HHH-SmallDependenceEmphasis	0.821	[0.46 0.95]
T2WI_wavelet_gldm_wavelet-HHH-SmallDependenceHighGrayLevelEm	0.997	[0.99 1.]
T2WI_wavelet_gldm_wavelet-LLL-DependenceEntropy	0.888	[0.63 0.97]
T2WI_wavelet_gldm_wavelet-LLL-DependenceNonUniformity	0.927	[0.51 0.98]
T2WI_wavelet_gldm_wavelet-LLL-DependenceNonUniformityNormali	0.955	[0.84 0.99]
T2WI_wavelet_gldm_wavelet-LLL-DependenceVariance	0.979	[0.92 0.99]
T2WI_wavelet_gldm_wavelet-LLL-GrayLevelNonUniformity	0.947	[0.62 0.99]
T2WI_wavelet_gldm_wavelet-LLL-GrayLevelVariance	0.982	[0.93 1.]
T2WI_wavelet_gldm_wavelet-LLL-HighGrayLevelEmphasis	0.983	[0.93 1.]
T2WI_wavelet_gldm_wavelet-LLL-LargeDependenceEmphasis	0.966	[0.87 0.99]
T2WI_wavelet_gldm_wavelet-LLL-LargeDependenceHighGrayLevelEm	0.88	[0.57 0.97]
T2WI_wavelet_gldm_wavelet-LLL-SmallDependenceEmphasis	0.957	[0.84 0.99]
T2WI_wavelet_gldm_wavelet-LLL-SmallDependenceHighGrayLevelEm	0.994	[0.98 1.]
T2WI_wavelet_ngtdm_wavelet-LLH-Busyness	0.921	[0.72 0.98]

T2WI_wavelet_ngtdm_wavelet-LLH-Complexity	0.955	[0.84 0.99]
T2WI_wavelet_ngtdm_wavelet-LLH-Contrast	0.958	[0.84 0.99]
T2WI_wavelet_ngtdm_wavelet-HLL-Complexity	0.811	[0.43 0.95]
T2WI_wavelet_ngtdm_wavelet-HLH-Busyness	0.82	[0.42 0.95]
T2WI_wavelet_ngtdm_wavelet-HLH-Complexity	0.981	[0.93 1.]
T2WI_wavelet_ngtdm_wavelet-HLH-Strength	0.954	[0.84 0.99]
T2WI_wavelet_ngtdm_wavelet-HHL-Busyness	0.958	[0.84 0.99]
T2WI_wavelet_ngtdm_wavelet-HHL-Complexity	0.952	[0.83 0.99]
T2WI_wavelet_ngtdm_wavelet-HHL-Strength	0.893	[0.58 0.97]
T2WI_wavelet_ngtdm_wavelet-HHH-Busyness	0.988	[0.95 1.]
T2WI_wavelet_ngtdm_wavelet-HHH-Complexity	0.948	[0.81 0.99]
T2WI_wavelet_ngtdm_wavelet-HHH-Strength	0.89	[0.63 0.97]
T2WI_wavelet_ngtdm_wavelet-LLL-Busyness	0.956	[0.83 0.99]
T2WI_wavelet_ngtdm_wavelet-LLL-Complexity	0.987	[0.95 1.]
T2WI_wavelet_ngtdm_wavelet-LLL-Contrast	0.784	[0.37 0.94]
T2WI_wavelet_ngtdm_wavelet-LLL-Strength	0.751	[0.29 0.93]
ADC_wavelet_firstorder_wavelet-LLH-Energy	0.828	[0.47 0.95]
ADC_wavelet_firstorder_wavelet-LLH-Uniformity	0.767	[0.32 0.94]
ADC_wavelet_firstorder_wavelet-LHL-10Percentile	0.942	[0.78 0.99]
ADC_wavelet_firstorder_wavelet-LHL-90Percentile	0.954	[0.72 0.99]
ADC_wavelet_firstorder_wavelet-LHL-Energy	0.933	[0.74 0.98]
ADC_wavelet_firstorder_wavelet-LHL-Entropy	0.879	[0.61 0.97]
ADC_wavelet_firstorder_wavelet-LHL-InterquartileRange	0.966	[0.88 0.99]
ADC_wavelet_firstorder_wavelet-LHL-MeanAbsoluteDeviation	0.949	[0.82 0.99]
ADC_wavelet_firstorder_wavelet-LHL-Mean	0.806	[0.42 0.95]
ADC_wavelet_firstorder_wavelet-LHL-Range	0.811	[0.44 0.95]
ADC_wavelet_firstorder_wavelet-LHL-RobustMeanAbsoluteDeviati	0.976	[0.91 0.99]
ADC_wavelet_firstorder_wavelet-LHL-RootMeanSquared	0.906	[0.68 0.98]
ADC_wavelet_firstorder_wavelet-LHL-TotalEnergy	0.911	[0.66 0.98]
ADC_wavelet_firstorder_wavelet-LHL-Uniformity	0.904	[0.68 0.97]
ADC_wavelet_firstorder_wavelet-LHL-Variance	0.939	[0.79 0.98]
ADC_wavelet_firstorder_wavelet-LHH-10Percentile	0.954	[0.83 0.99]
ADC_wavelet_firstorder_wavelet-LHH-90Percentile	0.968	[0.81 0.99]
ADC_wavelet_firstorder_wavelet-LHH-Energy	0.93	[0.73 0.98]
ADC_wavelet_firstorder_wavelet-LHH-Entropy	0.963	[0.85 0.99]
ADC_wavelet_firstorder_wavelet-LHH-InterquartileRange	0.988	[0.9 1.]
ADC_wavelet_firstorder_wavelet-LHH-MeanAbsoluteDeviation	0.977	[0.91 0.99]
ADC_wavelet_firstorder_wavelet-LHH-RobustMeanAbsoluteDeviati	0.98	[0.92 1.]
ADC_wavelet_firstorder_wavelet-LHH-RootMeanSquared	0.954	[0.83 0.99]
ADC_wavelet_firstorder_wavelet-LHH-TotalEnergy	0.919	[0.67 0.98]
ADC_wavelet_firstorder_wavelet-LHH-Uniformity	0.96	[0.84 0.99]
ADC_wavelet_firstorder_wavelet-LHH-Variance	0.962	[0.86 0.99]
ADC_wavelet_firstorder_wavelet-HLL-10Percentile	0.946	[0.81 0.99]
ADC_wavelet_firstorder_wavelet-HLL-90Percentile	0.978	[0.91 0.99]
ADC_wavelet_firstorder_wavelet-HLL-Energy	0.887	[0.63 0.97]
ADC_wavelet_firstorder_wavelet-HLL-Entropy	0.967	[0.88 0.99]
ADC_wavelet_firstorder_wavelet-HLL-InterquartileRange	0.993	[0.97 1.]
ADC_wavelet_firstorder_wavelet-HLL-Kurtosis	0.836	[0.5 0.96]
ADC_wavelet_firstorder_wavelet-HLL-MeanAbsoluteDeviation	0.982	[0.87 1.]
ADC_wavelet_firstorder_wavelet-HLL-RobustMeanAbsoluteDeviati	0.991	[0.97 1.]
ADC_wavelet_firstorder_wavelet-HLL-RootMeanSquared	0.968	[0.77 0.99]
ADC_wavelet_firstorder_wavelet-HLL-TotalEnergy	0.854	[0.54 0.96]

ADC_wavelet_firstorder_wavelet-HLL-Uniformity	0.983	[0.94 1.]
ADC_wavelet_firstorder_wavelet-HLL-Variance	0.972	[0.82 0.99]
ADC_wavelet_firstorder_wavelet-HLH-10Percentile	0.989	[0.96 1.]
ADC_wavelet_firstorder_wavelet-HLH-90Percentile	0.972	[0.89 0.99]
ADC_wavelet_firstorder_wavelet-HLH-Energy	0.912	[0.68 0.98]
ADC_wavelet_firstorder_wavelet-HLH-Entropy	0.981	[0.93 1.]
ADC_wavelet_firstorder_wavelet-HLH-InterquartileRange	0.969	[0.89 0.99]
ADC_wavelet_firstorder_wavelet-HLH-Kurtosis	0.756	[0.32 0.93]
ADC_wavelet_firstorder_wavelet-HLH-Maximum	0.778	[0.24 0.94]
ADC_wavelet_firstorder_wavelet-HLH-MeanAbsoluteDeviation	0.979	[0.92 0.99]
ADC_wavelet_firstorder_wavelet-HLH-Minimum	0.771	[0.35 0.94]
ADC_wavelet_firstorder_wavelet-HLH-Range	0.806	[0.37 0.95]
ADC_wavelet_firstorder_wavelet-HLH-RobustMeanAbsoluteDeviati	0.979	[0.92 0.99]
ADC_wavelet_firstorder_wavelet-HLH-RootMeanSquared	0.978	[0.91 0.99]
ADC_wavelet_firstorder_wavelet-HLH-TotalEnergy	0.889	[0.59 0.97]
ADC_wavelet_firstorder_wavelet-HLH-Uniformity	0.978	[0.92 0.99]
ADC_wavelet_firstorder_wavelet-HLH-Variance	0.976	[0.91 0.99]
ADC_wavelet_firstorder_wavelet-HHL-10Percentile	0.995	[0.98 1.]
ADC_wavelet_firstorder_wavelet-HHL-90Percentile	0.995	[0.98 1.]
ADC_wavelet_firstorder_wavelet-HHL-Energy	0.94	[0.77 0.98]
ADC_wavelet_firstorder_wavelet-HHL-Entropy	0.985	[0.95 1.]
ADC_wavelet_firstorder_wavelet-HHL-InterquartileRange	0.997	[0.99 1.]
ADC_wavelet_firstorder_wavelet-HHL-Maximum	0.807	[0.41 0.95]
ADC_wavelet_firstorder_wavelet-HHL-MeanAbsoluteDeviation	0.994	[0.98 1.]
ADC_wavelet_firstorder_wavelet-HHL-Median	0.902	[0.67 0.97]
ADC_wavelet_firstorder_wavelet-HHL-RobustMeanAbsoluteDeviati	0.997	[0.99 1.]
ADC_wavelet_firstorder_wavelet-HHL-RootMeanSquared	0.985	[0.94 1.]
ADC_wavelet_firstorder_wavelet-HHL-Skewness	0.797	[0.4 0.94]
ADC_wavelet_firstorder_wavelet-HHL-TotalEnergy	0.922	[0.71 0.98]
ADC_wavelet_firstorder_wavelet-HHL-Uniformity	0.992	[0.97 1.]
ADC_wavelet_firstorder_wavelet-HHL-Variance	0.99	[0.96 1.]
ADC_wavelet_firstorder_wavelet-HHH-10Percentile	0.988	[0.95 1.]
ADC_wavelet_firstorder_wavelet-HHH-90Percentile	0.985	[0.89 1.]
ADC_wavelet_firstorder_wavelet-HHH-Energy	0.924	[0.72 0.98]
ADC_wavelet_firstorder_wavelet-HHH-Entropy	0.978	[0.89 0.99]
ADC_wavelet_firstorder_wavelet-HHH-InterquartileRange	0.994	[0.98 1.]
ADC_wavelet_firstorder_wavelet-HHH-MeanAbsoluteDeviation	0.99	[0.96 1.]
ADC_wavelet_firstorder_wavelet-HHH-Minimum	0.891	[0.64 0.97]
ADC_wavelet_firstorder_wavelet-HHH-Range	0.798	[0.4 0.95]
ADC_wavelet_firstorder_wavelet-HHH-RobustMeanAbsoluteDeviati	0.994	[0.98 1.]
ADC_wavelet_firstorder_wavelet-HHH-RootMeanSquared	0.982	[0.93 1.]
ADC_wavelet_firstorder_wavelet-HHH-TotalEnergy	0.913	[0.67 0.98]
ADC_wavelet_firstorder_wavelet-HHH-Uniformity	0.977	[0.89 0.99]
ADC_wavelet_firstorder_wavelet-HHH-Variance	0.979	[0.92 0.99]
ADC_wavelet_firstorder_wavelet-LHH-Kurtosis	0.933	[0.78 0.98]
ADC_wavelet_firstorder_wavelet-LLH-Median	0.862	[0.54 0.97]
ADC_wavelet_firstorder_wavelet-LLL-10Percentile	0.833	[0.49 0.96]
ADC_wavelet_firstorder_wavelet-LLL-90Percentile	0.962	[0.86 0.99]
ADC_wavelet_firstorder_wavelet-LLL-Energy	0.956	[0.81 0.99]
ADC_wavelet_firstorder_wavelet-LLL-Entropy	0.83	[0.48 0.95]
ADC_wavelet_firstorder_wavelet-LLL-InterquartileRange	0.787	[0.37 0.94]
ADC_wavelet_firstorder_wavelet-LLL-Maximum	0.89	[0.55 0.97]

ADC_wavelet_firstorder_wavelet-LLL-MeanAbsoluteDeviation	0.828	[0.46 0.95]
ADC_wavelet_firstorder_wavelet-LLL-Mean	0.991	[0.77 1.]
ADC_wavelet_firstorder_wavelet-LLL-Median	0.985	[0.86 1.]
ADC_wavelet_firstorder_wavelet-LLL-RobustMeanAbsoluteDeviati	0.767	[0.32 0.94]
ADC_wavelet_firstorder_wavelet-LLL-RootMeanSquared	0.993	[0.85 1.]
ADC_wavelet_firstorder_wavelet-LLL-Skewness	0.908	[0.68 0.98]
ADC_wavelet_firstorder_wavelet-LLL-TotalEnergy	0.949	[0.73 0.99]
ADC_wavelet_firstorder_wavelet-LLL-Variance	0.872	[0.58 0.97]
ADC_wavelet_glcm_wavelet-LLH-InverseVariance	0.875	[0.59 0.97]
ADC_wavelet_glcm_wavelet-LLH-Id	0.829	[0.46 0.95]
ADC_wavelet_glcm_wavelet-LLH-Idm	0.881	[0.6 0.97]
ADC_wavelet_glcm_wavelet-LLH-Imc2	0.834	[0.49 0.96]
ADC_wavelet_glcm_wavelet-LHL-ClusterProminence	0.929	[0.75 0.98]
ADC_wavelet_glcm_wavelet-LHL-ClusterShade	0.82	[0.43 0.95]
ADC_wavelet_glcm_wavelet-LHL-ClusterTendency	0.945	[0.8 0.99]
ADC_wavelet_glcm_wavelet-LHL-Contrast	0.966	[0.87 0.99]
ADC_wavelet_glcm_wavelet-LHL-Correlation	0.945	[0.8 0.99]
ADC_wavelet_glcm_wavelet-LHL-DifferenceAverage	0.97	[0.89 0.99]
ADC_wavelet_glcm_wavelet-LHL-DifferenceEntropy	0.923	[0.73 0.98]
ADC_wavelet_glcm_wavelet-LHL-DifferenceVariance	0.941	[0.79 0.99]
ADC_wavelet_glcm_wavelet-LHL-InverseVariance	0.98	[0.92 0.99]
ADC_wavelet_glcm_wavelet-LHL-SumEntropy	0.84	[0.5 0.96]
ADC_wavelet_glcm_wavelet-LHL-Id	0.979	[0.92 0.99]
ADC_wavelet_glcm_wavelet-LHL-Idm	0.979	[0.92 0.99]
ADC_wavelet_glcm_wavelet-LHH-ClusterProminence	0.882	[0.59 0.97]
ADC_wavelet_glcm_wavelet-LHH-ClusterTendency	0.978	[0.92 0.99]
ADC_wavelet_glcm_wavelet-LHH-Contrast	0.983	[0.94 1.]
ADC_wavelet_glcm_wavelet-LHH-DifferenceAverage	0.987	[0.95 1.]
ADC_wavelet_glcm_wavelet-LHH-DifferenceEntropy	0.972	[0.89 0.99]
ADC_wavelet_glcm_wavelet-LHH-DifferenceVariance	0.966	[0.87 0.99]
ADC_wavelet_glcm_wavelet-LHH-JointEntropy	0.762	[0.33 0.93]
ADC_wavelet_glcm_wavelet-LHH-InverseVariance	0.981	[0.93 1.]
ADC_wavelet_glcm_wavelet-LHH-SumEntropy	0.96	[0.84 0.99]
ADC_wavelet_glcm_wavelet-LHH-Id	0.985	[0.94 1.]
ADC_wavelet_glcm_wavelet-LHH-Idm	0.985	[0.94 1.]
ADC_wavelet_glcm_wavelet-HLL-ClusterProminence	0.983	[0.94 1.]
ADC_wavelet_glcm_wavelet-HLL-ClusterShade	0.957	[0.84 0.99]
ADC_wavelet_glcm_wavelet-HLL-ClusterTendency	0.975	[0.88 0.99]
ADC_wavelet_glcm_wavelet-HLL-Contrast	0.977	[0.87 0.99]
ADC_wavelet_glcm_wavelet-HLL-Correlation	0.96	[0.83 0.99]
ADC_wavelet_glcm_wavelet-HLL-DifferenceAverage	0.982	[0.9 1.]
ADC_wavelet_glcm_wavelet-HLL-DifferenceEntropy	0.944	[0.8 0.99]
ADC_wavelet_glcm_wavelet-HLL-DifferenceVariance	0.966	[0.85 0.99]
ADC_wavelet_glcm_wavelet-HLL-InverseVariance	0.975	[0.88 0.99]
ADC_wavelet_glcm_wavelet-HLL-SumEntropy	0.872	[0.56 0.97]
ADC_wavelet_glcm_wavelet-HLL-Id	0.989	[0.95 1.]
ADC_wavelet_glcm_wavelet-HLL-Idm	0.989	[0.95 1.]
ADC_wavelet_glcm_wavelet-HLH-Autocorrelation	0.835	[0.49 0.96]
ADC_wavelet_glcm_wavelet-HLH-JointAverage	0.774	[0.36 0.94]
ADC_wavelet_glcm_wavelet-HLH-ClusterProminence	0.981	[0.93 1.]
ADC_wavelet_glcm_wavelet-HLH-ClusterTendency	0.98	[0.93 0.99]
ADC_wavelet_glcm_wavelet-HLH-Contrast	0.968	[0.88 0.99]

ADC_wavelet_glcm_wavelet-HLH-DifferenceAverage	0.976	[0.91 0.99]
ADC_wavelet_glcm_wavelet-HLH-DifferenceEntropy	0.985	[0.94 1.]
ADC_wavelet_glcm_wavelet-HLH-DifferenceVariance	0.963	[0.86 0.99]
ADC_wavelet_glcm_wavelet-HLH-InverseVariance	0.987	[0.95 1.]
ADC_wavelet_glcm_wavelet-HLH-SumEntropy	0.959	[0.85 0.99]
ADC_wavelet_glcm_wavelet-HLH-Id	0.981	[0.93 1.]
ADC_wavelet_glcm_wavelet-HLH-Idm	0.981	[0.93 1.]
ADC_wavelet_glcm_wavelet-HHL-ClusterProminence	0.977	[0.91 0.99]
ADC_wavelet_glcm_wavelet-HHL-ClusterShade	0.806	[0.39 0.95]
ADC_wavelet_glcm_wavelet-HHL-ClusterTendency	0.99	[0.96 1.]
ADC_wavelet_glcm_wavelet-HHL-Contrast	0.99	[0.96 1.]
ADC_wavelet_glcm_wavelet-HHL-Correlation	0.929	[0.75 0.98]
ADC_wavelet_glcm_wavelet-HHL-DifferenceAverage	0.994	[0.97 1.]
ADC_wavelet_glcm_wavelet-HHL-DifferenceEntropy	0.983	[0.93 1.]
ADC_wavelet_glcm_wavelet-HHL-DifferenceVariance	0.977	[0.91 0.99]
ADC_wavelet_glcm_wavelet-HHL-JointEnergy	0.855	[0.54 0.96]
ADC_wavelet_glcm_wavelet-HHL-JointEntropy	0.813	[0.44 0.95]
ADC_wavelet_glcm_wavelet-HHL-InverseVariance	0.995	[0.98 1.]
ADC_wavelet_glcm_wavelet-HHL-MaximumProbability	0.839	[0.5 0.96]
ADC_wavelet_glcm_wavelet-HHL-SumEntropy	0.97	[0.89 0.99]
ADC_wavelet_glcm_wavelet-HHL-Id	0.994	[0.97 1.]
ADC_wavelet_glcm_wavelet-HHL-Idm	0.994	[0.97 1.]
ADC_wavelet_glcm_wavelet-HHH-Autocorrelation	0.943	[0.8 0.99]
ADC_wavelet_glcm_wavelet-HHH-JointAverage	0.885	[0.62 0.97]
ADC_wavelet_glcm_wavelet-HHH-ClusterProminence	0.898	[0.64 0.97]
ADC_wavelet_glcm_wavelet-HHH-ClusterTendency	0.982	[0.93 1.]
ADC_wavelet_glcm_wavelet-HHH-Contrast	0.982	[0.93 1.]
ADC_wavelet_glcm_wavelet-HHH-DifferenceAverage	0.992	[0.97 1.]
ADC_wavelet_glcm_wavelet-HHH-DifferenceEntropy	0.975	[0.9 0.99]
ADC_wavelet_glcm_wavelet-HHH-DifferenceVariance	0.958	[0.85 0.99]
ADC_wavelet_glcm_wavelet-HHH-JointEnergy	0.913	[0.71 0.98]
ADC_wavelet_glcm_wavelet-HHH-JointEntropy	0.829	[0.48 0.95]
ADC_wavelet_glcm_wavelet-HHH-InverseVariance	0.992	[0.97 1.]
ADC_wavelet_glcm_wavelet-HHH-MaximumProbability	0.861	[0.56 0.96]
ADC_wavelet_glcm_wavelet-HHH-SumEntropy	0.966	[0.87 0.99]
ADC_wavelet_glcm_wavelet-HHH-Id	0.993	[0.97 1.]
ADC_wavelet_glcm_wavelet-HHH-Idm	0.992	[0.97 1.]
ADC_wavelet_glcm_wavelet-LLL-ClusterProminence	0.893	[0.65 0.97]
ADC_wavelet_glcm_wavelet-LLL-ClusterShade	0.882	[0.6 0.97]
ADC_wavelet_glcm_wavelet-LLL-ClusterTendency	0.922	[0.73 0.98]
ADC_wavelet_glrlm_wavelet-LLH-GrayLevelNonUniformityNormaliz	0.755	[0.31 0.93]
ADC_wavelet_glcm_wavelet-LLH-JointAverage	0.812	[0.43 0.92]
ADC_wavelet_glrlm_wavelet-LLH-LongRunEmphasis	0.876	[0.58 0.97]
ADC_wavelet_glrlm_wavelet-LLH-RunLengthNonUniformity	0.821	[0.41 0.95]
ADC_wavelet_glrlm_wavelet-LLH-RunLengthNonUniformityNormaliz	0.839	[0.47 0.96]
ADC_wavelet_glrlm_wavelet-LLH-RunPercentage	0.863	[0.54 0.96]
ADC_wavelet_glrlm_wavelet-LLH-RunVariance	0.901	[0.65 0.97]
ADC_wavelet_glrlm_wavelet-LLH-ShortRunEmphasis	0.843	[0.48 0.96]
ADC_wavelet_glrlm_wavelet-LHL-GrayLevelNonUniformityNormaliz	0.884	[0.62 0.97]
ADC_wavelet_glrlm_wavelet-LHL-GrayLevelVariance	0.937	[0.78 0.98]
ADC_wavelet_glrlm_wavelet-LHL-LongRunEmphasis	0.95	[0.81 0.99]
ADC_wavelet_glrlm_wavelet-LHL-RunEntropy	0.813	[0.44 0.95]

ADC_wavelet_glrlm_wavelet-LHL-RunLengthNonUniformity	0.826	[0.42 0.95]
ADC_wavelet_glrlm_wavelet-LHL-RunLengthNonUniformityNormaliz	0.937	[0.77 0.98]
ADC_wavelet_glrlm_wavelet-LHL-RunPercentage	0.948	[0.8 0.99]
ADC_wavelet_glrlm_wavelet-LHL-RunVariance	0.953	[0.82 0.99]
ADC_wavelet_glrlm_wavelet-LHL-ShortRunEmphasis	0.94	[0.78 0.98]
ADC_wavelet_glrlm_wavelet-LHH-GrayLevelNonUniformityNormaliz	0.956	[0.83 0.99]
ADC_wavelet_glrlm_wavelet-LHH-GrayLevelVariance	0.96	[0.85 0.99]
ADC_wavelet_glrlm_wavelet-LHH-LongRunEmphasis	0.953	[0.82 0.99]
ADC_wavelet_glrlm_wavelet-LHH-RunEntropy	0.946	[0.77 0.99]
ADC_wavelet_glrlm_wavelet-LHH-RunLengthNonUniformity	0.829	[0.43 0.96]
ADC_wavelet_glrlm_wavelet-LHH-RunLengthNonUniformityNormaliz	0.944	[0.79 0.99]
ADC_wavelet_glrlm_wavelet-LHH-RunPercentage	0.947	[0.8 0.99]
ADC_wavelet_glrlm_wavelet-LHH-RunVariance	0.955	[0.83 0.99]
ADC_wavelet_glrlm_wavelet-LHH-ShortRunEmphasis	0.946	[0.8 0.99]
ADC_wavelet_glrlm_wavelet-HLL-GrayLevelNonUniformity	0.767	[0.25 0.94]
ADC_wavelet_glrlm_wavelet-HLL-GrayLevelNonUniformityNormaliz	0.976	[0.91 0.99]
ADC_wavelet_glrlm_wavelet-HLL-GrayLevelVariance	0.971	[0.81 0.99]
ADC_wavelet_glrlm_wavelet-HLL-LongRunEmphasis	0.947	[0.81 0.99]
ADC_wavelet_glrlm_wavelet-HLL-RunEntropy	0.921	[0.72 0.98]
ADC_wavelet_glrlm_wavelet-HLL-RunLengthNonUniformity	0.82	[0.42 0.95]
ADC_wavelet_glrlm_wavelet-HLL-RunLengthNonUniformityNormaliz	0.937	[0.78 0.98]
ADC_wavelet_glrlm_wavelet-HLL-RunPercentage	0.941	[0.79 0.99]
ADC_wavelet_glrlm_wavelet-HLL-RunVariance	0.951	[0.82 0.99]
ADC_wavelet_glrlm_wavelet-HLL-ShortRunEmphasis	0.938	[0.78 0.98]
ADC_wavelet_glrlm_wavelet-HLH-GrayLevelNonUniformity	0.778	[0.29 0.94]
ADC_wavelet_glrlm_wavelet-HLH-GrayLevelNonUniformityNormaliz	0.977	[0.92 0.99]
ADC_wavelet_glrlm_wavelet-HLH-GrayLevelVariance	0.978	[0.91 0.99]
ADC_wavelet_glrlm_wavelet-HLH-HighGrayLevelRunEmphasis	0.842	[0.51 0.96]
ADC_wavelet_glrlm_wavelet-HLH-LongRunEmphasis	0.943	[0.79 0.99]
ADC_wavelet_glrlm_wavelet-HLH-LongRunHighGrayLevelEmphasis	0.82	[0.46 0.95]
ADC_wavelet_glrlm_wavelet-HLH-RunEntropy	0.955	[0.84 0.99]
ADC_wavelet_glrlm_wavelet-HLH-RunLengthNonUniformity	0.822	[0.42 0.95]
ADC_wavelet_glrlm_wavelet-HLH-RunLengthNonUniformityNormaliz	0.925	[0.74 0.98]
ADC_wavelet_glrlm_wavelet-HLH-RunPercentage	0.934	[0.77 0.98]
ADC_wavelet_glrlm_wavelet-HLH-RunVariance	0.939	[0.78 0.98]
ADC_wavelet_glrlm_wavelet-HLH-ShortRunEmphasis	0.927	[0.75 0.98]
ADC_wavelet_glrlm_wavelet-HLH-ShortRunHighGrayLevelEmphasis	0.847	[0.52 0.96]
ADC_wavelet_glrlm_wavelet-HHL-GrayLevelNonUniformity	0.757	[0.27 0.93]
ADC_wavelet_glrlm_wavelet-HHL-GrayLevelNonUniformityNormaliz	0.991	[0.96 1.]
ADC_wavelet_glrlm_wavelet-HHL-GrayLevelVariance	0.989	[0.96 1.]
ADC_wavelet_glrlm_wavelet-HHL-LongRunEmphasis	0.969	[0.88 0.99]
ADC_wavelet_glrlm_wavelet-HHL-RunEntropy	0.962	[0.86 0.99]
ADC_wavelet_glrlm_wavelet-HHL-RunLengthNonUniformity	0.827	[0.43 0.95]
ADC_wavelet_glrlm_wavelet-HHL-RunLengthNonUniformityNormaliz	0.964	[0.86 0.99]
ADC_wavelet_glrlm_wavelet-HHL-RunPercentage	0.967	[0.87 0.99]
ADC_wavelet_glrlm_wavelet-HHL-RunVariance	0.969	[0.88 0.99]
ADC_wavelet_glrlm_wavelet-HHL-ShortRunEmphasis	0.965	[0.87 0.99]
ADC_wavelet_glrlm_wavelet-HHH-GrayLevelNonUniformity	0.79	[0.35 0.94]
ADC_wavelet_glrlm_wavelet-HHH-GrayLevelNonUniformityNormaliz	0.978	[0.91 0.99]
ADC_wavelet_glrlm_wavelet-HHH-GrayLevelVariance	0.979	[0.92 0.99]
ADC_wavelet_glrlm_wavelet-HHH-HighGrayLevelRunEmphasis	0.945	[0.8 0.99]
ADC_wavelet_glrlm_wavelet-HHH-LongRunEmphasis	0.969	[0.88 0.99]

ADC_wavelet_glrlm_wavelet-HHH-LongRunHighGrayLevelEmphasis	0.931	[0.76 0.98]
ADC_wavelet_glrlm_wavelet-HHH-RunEntropy	0.947	[0.79 0.99]
ADC_wavelet_glrlm_wavelet-HHH-RunLengthNonUniformity	0.826	[0.43 0.95]
ADC_wavelet_glrlm_wavelet-HHH-RunLengthNonUniformityNormaliz	0.961	[0.85 0.99]
ADC_wavelet_glrlm_wavelet-HHH-RunPercentage	0.966	[0.87 0.99]
ADC_wavelet_glrlm_wavelet-HHH-RunVariance	0.972	[0.89 0.99]
ADC_wavelet_glrlm_wavelet-HHH-ShortRunEmphasis	0.964	[0.86 0.99]
ADC_wavelet_glrlm_wavelet-HHH-ShortRunHighGrayLevelEmphasis	0.948	[0.81 0.99]
ADC_wavelet_glrlm_wavelet-LLL-GrayLevelVariance	0.87	[0.58 0.97]
ADC_wavelet_glrlm_wavelet-LLL-RunEntropy	0.808	[0.43 0.95]
ADC_wavelet_glrlm_wavelet-LLL-RunLengthNonUniformity	0.818	[0.41 0.95]
ADC_wavelet_glszm_wavelet-LLH-LargeAreaEmphasis	0.945	[0.8 0.99]
ADC_wavelet_glszm_wavelet-LLH-SizeZoneNonUniformity	0.865	[0.49 0.97]
ADC_wavelet_glszm_wavelet-LLH-ZonePercentage	0.777	[0.31 0.94]
ADC_wavelet_glszm_wavelet-LLH-ZoneVariance	0.966	[0.88 0.99]
ADC_wavelet_glszm_wavelet-LHL-GrayLevelNonUniformityNormaliz	0.81	[0.43 0.95]
ADC_wavelet_glszm_wavelet-LHL-GrayLevelVariance	0.888	[0.62 0.97]
ADC_wavelet_glszm_wavelet-LHL-LargeAreaEmphasis	0.97	[0.89 0.99]
ADC_wavelet_glszm_wavelet-LHL-LargeAreaHighGrayLevelEmphasis	0.873	[0.58 0.97]
ADC_wavelet_glszm_wavelet-LHL-SizeZoneNonUniformity	0.888	[0.57 0.97]
ADC_wavelet_glszm_wavelet-LHL-SizeZoneNonUniformityNormalize	0.818	[0.42 0.95]
ADC_wavelet_glszm_wavelet-LHL-SmallAreaEmphasis	0.831	[0.45 0.96]
ADC_wavelet_glszm_wavelet-LHL-ZonePercentage	0.89	[0.62 0.97]
ADC_wavelet_glszm_wavelet-LHL-ZoneVariance	0.972	[0.9 0.99]
ADC_wavelet_glszm_wavelet-LHH-GrayLevelNonUniformityNormaliz	0.915	[0.68 0.98]
ADC_wavelet_glszm_wavelet-LHH-GrayLevelVariance	0.925	[0.73 0.98]
ADC_wavelet_glszm_wavelet-LHH-LargeAreaEmphasis	0.975	[0.91 0.99]
ADC_wavelet_glszm_wavelet-LHH-SizeZoneNonUniformity	0.895	[0.58 0.97]
ADC_wavelet_glszm_wavelet-LHH-SmallAreaHighGrayLevelEmphasis	0.765	[0.32 0.94]
ADC_wavelet_glszm_wavelet-LHH-ZonePercentage	0.9	[0.65 0.97]
ADC_wavelet_glszm_wavelet-LHH-ZoneVariance	0.976	[0.91 0.99]
ADC_wavelet_glszm_wavelet-HLL-GrayLevelNonUniformity	0.789	[0.32 0.94]
ADC_wavelet_glszm_wavelet-HLL-GrayLevelNonUniformityNormaliz	0.954	[0.83 0.99]
ADC_wavelet_glszm_wavelet-HLL-GrayLevelVariance	0.962	[0.81 0.99]
ADC_wavelet_glszm_wavelet-HLL-LargeAreaEmphasis	0.992	[0.97 1.]
ADC_wavelet_glszm_wavelet-HLL-LargeAreaHighGrayLevelEmphasis	0.881	[0.61 0.97]
ADC_wavelet_glszm_wavelet-HLL-SizeZoneNonUniformity	0.854	[0.51 0.96]
ADC_wavelet_glszm_wavelet-HLL-SizeZoneNonUniformityNormalize	0.808	[0.41 0.95]
ADC_wavelet_glszm_wavelet-HLL-SmallAreaEmphasis	0.839	[0.48 0.96]
ADC_wavelet_glszm_wavelet-HLL-ZonePercentage	0.916	[0.71 0.98]
ADC_wavelet_glszm_wavelet-HLL-ZoneVariance	0.993	[0.98 1.]
ADC_wavelet_glszm_wavelet-HLH-GrayLevelNonUniformity	0.783	[0.33 0.94]
ADC_wavelet_glszm_wavelet-HLH-GrayLevelNonUniformityNormaliz	0.943	[0.79 0.99]
ADC_wavelet_glszm_wavelet-HLH-GrayLevelVariance	0.982	[0.93 1.]
ADC_wavelet_glszm_wavelet-HLH-HighGrayLevelZoneEmphasis	0.841	[0.51 0.96]
ADC_wavelet_glszm_wavelet-HLH-LargeAreaEmphasis	0.981	[0.93 1.]
ADC_wavelet_glszm_wavelet-HLH-SizeZoneNonUniformity	0.867	[0.54 0.97]
ADC_wavelet_glszm_wavelet-HLH-SmallAreaEmphasis	0.757	[0.27 0.93]
ADC_wavelet_glszm_wavelet-HLH-SmallAreaHighGrayLevelEmphasis	0.868	[0.58 0.96]
ADC_wavelet_glszm_wavelet-HLH-ZonePercentage	0.896	[0.65 0.97]
ADC_wavelet_glszm_wavelet-HLH-ZoneVariance	0.982	[0.93 1.]
ADC_wavelet_glszm_wavelet-HHL-GrayLevelNonUniformity	0.763	[0.28 0.94]

ADC_wavelet_glszm_wavelet-HHL-GrayLevelNonUniformityNormaliz	0.942	[0.79 0.99]
ADC_wavelet_glszm_wavelet-HHL-GrayLevelVariance	0.958	[0.84 0.99]
ADC_wavelet_glszm_wavelet-HHL-LargeAreaEmphasis	0.999	[1. 1.]
ADC_wavelet_glszm_wavelet-HHL-SizeZoneNonUniformity	0.872	[0.54 0.97]
ADC_wavelet_glszm_wavelet-HHL-SizeZoneNonUniformityNormalize	0.916	[0.72 0.98]
ADC_wavelet_glszm_wavelet-HHL-SmallAreaEmphasis	0.922	[0.73 0.98]
ADC_wavelet_glszm_wavelet-HHL-ZonePercentage	0.941	[0.78 0.99]
ADC_wavelet_glszm_wavelet-HHL-ZoneVariance	0.999	[1. 1.]
ADC_wavelet_glszm_wavelet-HHH-GrayLevelNonUniformityNormaliz	0.93	[0.74 0.98]
ADC_wavelet_glszm_wavelet-HHH-GrayLevelVariance	0.951	[0.82 0.99]
ADC_wavelet_glszm_wavelet-HHH-HighGrayLevelZoneEmphasis	0.946	[0.8 0.99]
ADC_wavelet_glszm_wavelet-HHH-LargeAreaEmphasis	0.996	[0.98 1.]
ADC_wavelet_glszm_wavelet-HHH-LargeAreaLowGrayLevelEmphasis	0.789	[0.39 0.94]
ADC_wavelet_glszm_wavelet-HHH-LowGrayLevelZoneEmphasis	0.765	[0.32 0.96]
ADC_wavelet_glszm_wavelet-HHH-SizeZoneNonUniformity	0.877	[0.54 0.97]
ADC_wavelet_glszm_wavelet-HHH-SmallAreaHighGrayLevelEmphasis	0.952	[0.82 0.99]
ADC_wavelet_glszm_wavelet-HHH-ZonePercentage	0.936	[0.76 0.98]
ADC_wavelet_glszm_wavelet-HHH-ZoneVariance	0.996	[0.98 1.]
ADC_wavelet_glszm_wavelet-LLL-GrayLevelVariance	0.848	[0.52 0.96]
ADC_wavelet_glszm_wavelet-LLL-LargeAreaEmphasis	0.859	[0.52 0.96]
ADC_wavelet_glszm_wavelet-LLL-SizeZoneNonUniformity	0.832	[0.42 0.96]
ADC_wavelet_glszm_wavelet-LLL-ZoneVariance	0.913	[0.69 0.98]
ADC_wavelet_gldm_wavelet-LLH-DependenceNonUniformity	0.863	[0.49 0.97]
ADC_wavelet_gldm_wavelet-LLH-DependenceVariance	0.967	[0.87 0.99]
ADC_wavelet_gldm_wavelet-LLH-LargeDependenceEmphasis	0.922	[0.72 0.98]
ADC_wavelet_gldm_wavelet-LHL-DependenceNonUniformity	0.874	[0.54 0.97]
ADC_wavelet_gldm_wavelet-LHL-DependenceNonUniformityNormaliz	0.88	[0.59 0.97]
ADC_wavelet_gldm_wavelet-LHL-DependenceVariance	0.984	[0.94 1.]
ADC_wavelet_gldm_wavelet-LHL-GrayLevelVariance	0.939	[0.79 0.98]
ADC_wavelet_gldm_wavelet-LHL-LargeDependenceEmphasis	0.963	[0.86 0.99]
ADC_wavelet_gldm_wavelet-LHL-SmallDependenceEmphasis	0.879	[0.58 0.97]
ADC_wavelet_gldm_wavelet-LHH-DependenceNonUniformity	0.888	[0.57 0.97]
ADC_wavelet_gldm_wavelet-LHH-DependenceNonUniformityNormaliz	0.887	[0.61 0.97]
ADC_wavelet_gldm_wavelet-LHH-DependenceVariance	0.982	[0.93 1.]
ADC_wavelet_gldm_wavelet-LHH-GrayLevelVariance	0.961	[0.85 0.99]
ADC_wavelet_gldm_wavelet-LHH-LargeDependenceEmphasis	0.966	[0.87 0.99]
ADC_wavelet_gldm_wavelet-LHH-SmallDependenceEmphasis	0.886	[0.6 0.97]
ADC_wavelet_gldm_wavelet-LHH-SmallDependenceHighGrayLevelEmp	0.829	[0.47 0.95]
ADC_wavelet_gldm_wavelet-HLL-DependenceNonUniformity	0.847	[0.49 0.96]
ADC_wavelet_gldm_wavelet-HLL-DependenceNonUniformityNormaliz	0.829	[0.48 0.95]
ADC_wavelet_gldm_wavelet-HLL-DependenceVariance	0.958	[0.84 0.99]
ADC_wavelet_gldm_wavelet-HLL-GrayLevelNonUniformity	0.768	[0.25 0.94]
ADC_wavelet_gldm_wavelet-HLL-GrayLevelVariance	0.971	[0.81 0.99]
ADC_wavelet_gldm_wavelet-HLL-LargeDependenceEmphasis	0.96	[0.85 0.99]
ADC_wavelet_gldm_wavelet-HLL-SmallDependenceEmphasis	0.884	[0.62 0.97]
ADC_wavelet_gldm_wavelet-HLH-DependenceNonUniformity	0.856	[0.52 0.96]
ADC_wavelet_gldm_wavelet-HLH-DependenceNonUniformityNormaliz	0.786	[0.37 0.94]
ADC_wavelet_gldm_wavelet-HLH-DependenceVariance	0.96	[0.85 0.99]
ADC_wavelet_gldm_wavelet-HLH-GrayLevelNonUniformity	0.785	[0.3 0.94]
ADC_wavelet_gldm_wavelet-HLH-GrayLevelVariance	0.977	[0.91 0.99]
ADC_wavelet_gldm_wavelet-HLH-HighGrayLevelEmphasis	0.842	[0.51 0.96]
ADC_wavelet_gldm_wavelet-HLH-LargeDependenceEmphasis	0.956	[0.84 0.99]

ADC_wavelet_gldm_wavelet-HLH-SmallDependenceEmphasis	0.86 [0.54 0.96]
ADC_wavelet_gldm_wavelet-HLH-SmallDependenceHighGrayLevelEmp	0.918 [0.72 0.98]
ADC_wavelet_gldm_wavelet-HHL-DependenceNonUniformity	0.875 [0.55 0.97]
ADC_wavelet_gldm_wavelet-HHL-DependenceNonUniformityNormaliz	0.914 [0.7 0.98]
ADC_wavelet_gldm_wavelet-HHL-DependenceVariance	0.967 [0.88 0.99]
ADC_wavelet_gldm_wavelet-HHL-EpendenceNonUniformityNormalize	0.855 [0.62 0.98]
ADC_wavelet_gldm_wavelet-HHL-GrayLevelNonUniformity	0.775 [0.3 0.94]
ADC_wavelet_gldm_wavelet-HHL-GrayLevelVariance	0.99 [0.96 1.]
ADC_wavelet_gldm_wavelet-HHL-LargeDependenceEmphasis	0.974 [0.9 0.99]
ADC_wavelet_gldm_wavelet-HHL-SmallDependenceEmphasis	0.941 [0.78 0.98]
ADC_wavelet_gldm_wavelet-HHH-DependenceNonUniformity	0.869 [0.54 0.97]
ADC_wavelet_gldm_wavelet-HHH-DependenceNonUniformityNormaliz	0.891 [0.63 0.97]
ADC_wavelet_gldm_wavelet-HHH-DependenceVariance	0.969 [0.88 0.99]
ADC_wavelet_gldm_wavelet-HHH-GrayLevelNonUniformity	0.809 [0.39 0.95]
ADC_wavelet_gldm_wavelet-HHH-GrayLevelVariance	0.98 [0.92 0.99]
ADC_wavelet_gldm_wavelet-HHH-HighGrayLevelEmphasis	0.945 [0.8 0.99]
ADC_wavelet_gldm_wavelet-HHH-LargeDependenceEmphasis	0.974 [0.9 0.99]
ADC_wavelet_gldm_wavelet-HHH-SmallDependenceEmphasis	0.923 [0.72 0.98]
ADC_wavelet_gldm_wavelet-HHH-SmallDependenceHighGrayLevelEmp	0.976 [0.9 0.99]
ADC_wavelet_gldm_wavelet-LLL-DependenceNonUniformity	0.833 [0.42 0.96]
ADC_wavelet_gldm_wavelet-LLL-DependenceVariance	0.801 [0.37 0.95]
ADC_wavelet_gldm_wavelet-LLL-GrayLevelVariance	0.872 [0.58 0.97]
ADC_wavelet_gldm_wavelet-LHL-Dependence Entropy	0.864 [0.54 0.95]
ADC_wavelet_ngtdm_wavelet-LLH-Busyness	0.813 [0.4 0.95]
ADC_wavelet_ngtdm_wavelet-LHL-Complexity	0.904 [0.67 0.97]
ADC_wavelet_ngtdm_wavelet-LHH-Complexity	0.844 [0.52 0.96]
ADC_wavelet_ngtdm_wavelet-HLL-Complexity	0.913 [0.69 0.98]
ADC_wavelet_ngtdm_wavelet-HLH-Complexity	0.96 [0.84 0.99]
ADC_wavelet_ngtdm_wavelet-HHL-Complexity	0.959 [0.85 0.99]
ADC_wavelet_ngtdm_wavelet-HHH-Complexity	0.91 [0.68 0.98]
ADC_wavelet_ngtdm_wavelet-LLL-Busyness	0.812 [0.44 0.95]
DWI_wavelet_firstorder_wavelet-LLH-10Percentile	0.942 [0.8 0.98]
DWI_wavelet_firstorder_wavelet-LLH-90Percentile	0.923 [0.69 0.98]
DWI_wavelet_firstorder_wavelet-LLH-Energy	0.933 [0.7 0.98]
DWI_wavelet_firstorder_wavelet-LLH-Entropy	0.974 [0.9 0.99]
DWI_wavelet_firstorder_wavelet-LLH-InterquartileRange	0.971 [0.88 0.99]
DWI_wavelet_firstorder_wavelet-LLH-Kurtosis	0.758 [0.28 0.93]
DWI_wavelet_firstorder_wavelet-LLH-Maximum	0.951 [0.82 0.99]
DWI_wavelet_firstorder_wavelet-LLH-MeanAbsoluteDeviation	0.962 [0.85 0.99]
DWI_wavelet_firstorder_wavelet-LLH-Mean	0.777 [0.32 0.94]
DWI_wavelet_firstorder_wavelet-LLH-Minimum	0.939 [0.79 0.98]
DWI_wavelet_firstorder_wavelet-LLH-Range	0.976 [0.87 0.99]
DWI_wavelet_firstorder_wavelet-LLH-RobustMeanAbsoluteDeviati	0.965 [0.86 0.99]
DWI_wavelet_firstorder_wavelet-LLH-RootMeanSquared	0.953 [0.82 0.99]
DWI_wavelet_firstorder_wavelet-LLH-TotalEnergy	0.939 [0.61 0.99]
DWI_wavelet_firstorder_wavelet-LLH-Uniformity	0.976 [0.91 0.99]
DWI_wavelet_firstorder_wavelet-LLH-Variance	0.931 [0.69 0.98]
DWI_wavelet_firstorder_wavelet-LHL-10Percentile	0.949 [0.49 0.99]
DWI_wavelet_firstorder_wavelet-LHL-90Percentile	0.986 [0.88 1.]
DWI_wavelet_firstorder_wavelet-LHL-Energy	0.912 [0.64 0.98]
DWI_wavelet_firstorder_wavelet-LHL-Entropy	0.976 [0.75 1.]
DWI_wavelet_firstorder_wavelet-LHL-InterquartileRange	0.981 [0.77 1.]

DWI_wavelet_firstorder_wavelet-LHL-MeanAbsoluteDeviation	0.981	[0.76 1.]
DWI_wavelet_firstorder_wavelet-LHL-Mean	0.882	[0.22 0.98]
DWI_wavelet_firstorder_wavelet-LHL-Median	0.816	[0.18 0.96]
DWI_wavelet_firstorder_wavelet-LHL-RobustMeanAbsoluteDeviati	0.977	[0.67 1.]
DWI_wavelet_firstorder_wavelet-LHL-RootMeanSquared	0.959	[0.85 0.99]
DWI_wavelet_firstorder_wavelet-LHL-TotalEnergy	0.91	[0.55 0.98]
DWI_wavelet_firstorder_wavelet-LHL-Uniformity	0.973	[0.65 0.99]
DWI_wavelet_firstorder_wavelet-LHL-Variance	0.93	[0.76 0.98]
DWI_wavelet_firstorder_wavelet-LHH-10Percentile	0.976	[0.72 1.]
DWI_wavelet_firstorder_wavelet-LHH-90Percentile	0.961	[0.82 0.99]
DWI_wavelet_firstorder_wavelet-LHH-Energy	0.944	[0.66 0.99]
DWI_wavelet_firstorder_wavelet-LHH-Entropy	0.943	[0.64 0.99]
DWI_wavelet_firstorder_wavelet-LHH-InterquartileRange	0.978	[0.91 0.99]
DWI_wavelet_firstorder_wavelet-LHH-Maximum	0.821	[0.46 0.95]
DWI_wavelet_firstorder_wavelet-LHH-MeanAbsoluteDeviation	0.965	[0.81 0.99]
DWI_wavelet_firstorder_wavelet-LHH-RobustMeanAbsoluteDeviati	0.967	[0.86 0.99]
DWI_wavelet_firstorder_wavelet-LHH-RootMeanSquared	0.959	[0.77 0.99]
DWI_wavelet_firstorder_wavelet-LHH-Skewness	0.768	[0.29 0.94]
DWI_wavelet_firstorder_wavelet-LHH-TotalEnergy	0.936	[0.45 0.99]
DWI_wavelet_firstorder_wavelet-LHH-Uniformity	0.943	[0.65 0.99]
DWI_wavelet_firstorder_wavelet-LHH-Variance	0.944	[0.68 0.99]
DWI_wavelet_firstorder_wavelet-HLL-10Percentile	0.94	[0.78 0.98]
DWI_wavelet_firstorder_wavelet-HLL-90Percentile	0.991	[0.96 1.]
DWI_wavelet_firstorder_wavelet-HLL-Energy	0.974	[0.86 0.99]
DWI_wavelet_firstorder_wavelet-HLL-Entropy	0.984	[0.94 1.]
DWI_wavelet_firstorder_wavelet-HLL-InterquartileRange	0.985	[0.94 1.]
DWI_wavelet_firstorder_wavelet-HLL-Maximum	0.903	[0.68 0.97]
DWI_wavelet_firstorder_wavelet-HLL-MeanAbsoluteDeviation	0.983	[0.93 1.]
DWI_wavelet_firstorder_wavelet-HLL-Mean	0.824	[0.27 0.96]
DWI_wavelet_firstorder_wavelet-HLL-Median	0.756	[0.19 0.94]
DWI_wavelet_firstorder_wavelet-HLL-Minimum	0.969	[0.88 0.99]
DWI_wavelet_firstorder_wavelet-HLL-Range	0.947	[0.81 0.99]
DWI_wavelet_firstorder_wavelet-HLL-RobustMeanAbsoluteDeviati	0.985	[0.94 1.]
DWI_wavelet_firstorder_wavelet-HLL-RootMeanSquared	0.976	[0.91 0.99]
DWI_wavelet_firstorder_wavelet-HLL-TotalEnergy	0.974	[0.88 0.99]
DWI_wavelet_firstorder_wavelet-HLL-Uniformity	0.985	[0.94 1.]
DWI_wavelet_firstorder_wavelet-HLL-Variance	0.967	[0.87 0.99]
DWI_wavelet_firstorder_wavelet-HLH-10Percentile	0.951	[0.83 0.99]
DWI_wavelet_firstorder_wavelet-HLH-90Percentile	0.941	[0.79 0.98]
DWI_wavelet_firstorder_wavelet-HLH-Energy	0.973	[0.77 0.99]
DWI_wavelet_firstorder_wavelet-HLH-Entropy	0.983	[0.93 1.]
DWI_wavelet_firstorder_wavelet-HLH-InterquartileRange	0.966	[0.87 0.99]
DWI_wavelet_firstorder_wavelet-HLH-Maximum	0.851	[0.51 0.96]
DWI_wavelet_firstorder_wavelet-HLH-MeanAbsoluteDeviation	0.974	[0.9 0.99]
DWI_wavelet_firstorder_wavelet-HLH-Minimum	0.99	[0.96 1.]
DWI_wavelet_firstorder_wavelet-HLH-Range	0.972	[0.89 0.99]
DWI_wavelet_firstorder_wavelet-HLH-RobustMeanAbsoluteDeviati	0.965	[0.87 0.99]
DWI_wavelet_firstorder_wavelet-HLH-RootMeanSquared	0.987	[0.95 1.]
DWI_wavelet_firstorder_wavelet-HLH-TotalEnergy	0.969	[0.59 0.99]
DWI_wavelet_firstorder_wavelet-HLH-Uniformity	0.975	[0.89 0.99]
DWI_wavelet_firstorder_wavelet-HLH-Variance	0.985	[0.94 1.]
DWI_wavelet_firstorder_wavelet-HHL-10Percentile	0.99	[0.83 1.]

DWI_wavelet_firstorder_wavelet-HHL-90Percentile	0.988	[0.74 1.]
DWI_wavelet_firstorder_wavelet-HHL-Energy	0.959	[0.75 0.99]
DWI_wavelet_firstorder_wavelet-HHL-Entropy	0.981	[0.83 1.]
DWI_wavelet_firstorder_wavelet-HHL-InterquartileRange	0.991	[0.88 1.]
DWI_wavelet_firstorder_wavelet-HHL-Kurtosis	0.926	[0.74 0.98]
DWI_wavelet_firstorder_wavelet-HHL-Maximum	0.924	[0.61 0.98]
DWI_wavelet_firstorder_wavelet-HHL-MeanAbsoluteDeviation	0.988	[0.79 1.]
DWI_wavelet_firstorder_wavelet-HHL-Median	0.765	[0.29 0.94]
DWI_wavelet_firstorder_wavelet-HHL-Minimum	0.933	[0.73 0.98]
DWI_wavelet_firstorder_wavelet-HHL-Range	0.932	[0.67 0.98]
DWI_wavelet_firstorder_wavelet-HHL-RobustMeanAbsoluteDeviati	0.991	[0.8 1.]
DWI_wavelet_firstorder_wavelet-HHL-RootMeanSquared	0.986	[0.78 1.]
DWI_wavelet_firstorder_wavelet-HHL-TotalEnergy	0.953	[0.53 0.99]
DWI_wavelet_firstorder_wavelet-HHL-Uniformity	0.984	[0.85 1.]
DWI_wavelet_firstorder_wavelet-HHL-Variance	0.984	[0.71 1.]
DWI_wavelet_firstorder_wavelet-HHH-10Percentile	0.989	[0.96 1.]
DWI_wavelet_firstorder_wavelet-HHH-90Percentile	0.98	[0.93 1.]
DWI_wavelet_firstorder_wavelet-HHH-Energy	0.968	[0.83 0.99]
DWI_wavelet_firstorder_wavelet-HHH-Entropy	0.991	[0.96 1.]
DWI_wavelet_firstorder_wavelet-HHH-InterquartileRange	0.99	[0.96 1.]
DWI_wavelet_firstorder_wavelet-HHH-Maximum	0.968	[0.88 0.99]
DWI_wavelet_firstorder_wavelet-HHH-MeanAbsoluteDeviation	0.991	[0.96 1.]
DWI_wavelet_firstorder_wavelet-HHH-Minimum	0.917	[0.7 0.98]
DWI_wavelet_firstorder_wavelet-HHH-Range	0.956	[0.84 0.99]
DWI_wavelet_firstorder_wavelet-HHH-RobustMeanAbsoluteDeviati	0.988	[0.95 1.]
DWI_wavelet_firstorder_wavelet-HHH-RootMeanSquared	0.994	[0.97 1.]
DWI_wavelet_firstorder_wavelet-HHH-Skewness	0.767	[0.29 0.94]
DWI_wavelet_firstorder_wavelet-HHH-TotalEnergy	0.963	[0.7 0.99]
DWI_wavelet_firstorder_wavelet-HHH-Uniformity	0.989	[0.95 1.]
DWI_wavelet_firstorder_wavelet-HHH-Variance	0.993	[0.97 1.]
DWI_wavelet_firstorder_wavelet-LHL-Skewness	0.937	[0.68 0.98]
DWI_wavelet_firstorder_wavelet-LLL-10Percentile	0.98	[0.92 0.99]
DWI_wavelet_firstorder_wavelet-LLL-90Percentile	0.995	[0.98 1.]
DWI_wavelet_firstorder_wavelet-LLL-Energy	0.99	[0.93 1.]
DWI_wavelet_firstorder_wavelet-LLL-Entropy	0.981	[0.93 1.]
DWI_wavelet_firstorder_wavelet-LLL-InterquartileRange	0.935	[0.78 0.98]
DWI_wavelet_firstorder_wavelet-LLL-Maximum	0.988	[0.95 1.]
DWI_wavelet_firstorder_wavelet-LLL-MeanAbsoluteDeviation	0.984	[0.94 1.]
DWI_wavelet_firstorder_wavelet-LLL-Mean	0.993	[0.97 1.]
DWI_wavelet_firstorder_wavelet-LLL-Median	0.994	[0.98 1.]
DWI_wavelet_firstorder_wavelet-LLL-Range	0.986	[0.95 1.]
DWI_wavelet_firstorder_wavelet-LLL-RobustMeanAbsoluteDeviati	0.957	[0.85 0.99]
DWI_wavelet_firstorder_wavelet-LLL-RootMeanSquared	0.994	[0.98 1.]
DWI_wavelet_firstorder_wavelet-LLL-Skewness	0.896	[0.63 0.97]
DWI_wavelet_firstorder_wavelet-LLL-TotalEnergy	0.982	[0.76 1.]
DWI_wavelet_firstorder_wavelet-LLL-Uniformity	0.938	[0.78 0.98]
DWI_wavelet_firstorder_wavelet-LLL-Variance	0.994	[0.98 1.]
DWI_wavelet_glcm_wavelet-LLH-Autocorrelation	0.915	[0.71 0.98]
DWI_wavelet_glcm_wavelet-LLH-JointAverage	0.926	[0.74 0.98]
DWI_wavelet_glcm_wavelet-LLH-ClusterProminence	0.871	[0.49 0.97]
DWI_wavelet_glcm_wavelet-LLH-ClusterShade	0.805	[0.41 0.95]
DWI_wavelet_glcm_wavelet-LLH-ClusterTendency	0.963	[0.77 0.99]

DWI_wavelet_glcm_wavelet-LLH-Contrast	0.837 [0.47 0.96]
DWI_wavelet_glcm_wavelet-LLH-DifferenceAverage	0.91 [0.68 0.98]
DWI_wavelet_glcm_wavelet-LLH-DifferenceEntropy	0.947 [0.81 0.99]
DWI_wavelet_glcm_wavelet-LLH-DifferenceVariance	0.83 [0.46 0.95]
DWI_wavelet_glcm_wavelet-LLH-JointEnergy	0.986 [0.95 1.]
DWI_wavelet_glcm_wavelet-LLH-JointEntropy	0.977 [0.9 0.99]
DWI_wavelet_glcm_wavelet-LLH-InverseVariance	0.784 [0.37 0.94]
DWI_wavelet_glcm_wavelet-LLH-MaximumProbability	0.992 [0.94 1.]
DWI_wavelet_glcm_wavelet-LLH-SumEntropy	0.991 [0.95 1.]
DWI_wavelet_glcm_wavelet-LLH-Id	0.966 [0.87 0.99]
DWI_wavelet_glcm_wavelet-LLH-Idm	0.966 [0.87 0.99]
DWI_wavelet_glcm_wavelet-LLH-Imc2	0.857 [0.52 0.96]
DWI_wavelet_glcm_wavelet-LHL-ClusterTendency	0.944 [0.8 0.99]
DWI_wavelet_glcm_wavelet-LHL-Contrast	0.912 [0.7 0.98]
DWI_wavelet_glcm_wavelet-LHL-Correlation	0.936 [0.76 0.98]
DWI_wavelet_glcm_wavelet-LHL-DifferenceAverage	0.963 [0.78 0.99]
DWI_wavelet_glcm_wavelet-LHL-DifferenceEntropy	0.963 [0.77 0.99]
DWI_wavelet_glcm_wavelet-LHL-DifferenceVariance	0.841 [0.51 0.96]
DWI_wavelet_glcm_wavelet-LHL-JointEnergy	0.971 [0.78 0.99]
DWI_wavelet_glcm_wavelet-LHL-JointEntropy	0.969 [0.75 0.99]
DWI_wavelet_glcm_wavelet-LHL-InverseVariance	0.96 [0.85 0.99]
DWI_wavelet_glcm_wavelet-LHL-MaximumProbability	0.967 [0.88 0.99]
DWI_wavelet_glcm_wavelet-LHL-SumEntropy	0.971 [0.78 0.99]
DWI_wavelet_glcm_wavelet-LHL-Id	0.971 [0.84 0.99]
DWI_wavelet_glcm_wavelet-LHL-Idm	0.971 [0.81 0.99]
DWI_wavelet_glcm_wavelet-LHL-Imc2	0.877 [0.58 0.97]
DWI_wavelet_glcm_wavelet-LHL-Imc1	0.808 [0.42 0.95]
DWI_wavelet_glcm_wavelet-LHH-ClusterTendency	0.904 [0.53 0.98]
DWI_wavelet_glcm_wavelet-LHH-Contrast	0.885 [0.48 0.97]
DWI_wavelet_glcm_wavelet-LHH-DifferenceAverage	0.917 [0.58 0.98]
DWI_wavelet_glcm_wavelet-LHH-DifferenceEntropy	0.92 [0.59 0.98]
DWI_wavelet_glcm_wavelet-LHH-DifferenceVariance	0.841 [0.39 0.96]
DWI_wavelet_glcm_wavelet-LHH-JointEnergy	0.928 [0.65 0.98]
DWI_wavelet_glcm_wavelet-LHH-JointEntropy	0.929 [0.62 0.98]
DWI_wavelet_glcm_wavelet-LHH-MaximumProbability	0.939 [0.6 0.99]
DWI_wavelet_glcm_wavelet-LHH-SumEntropy	0.932 [0.63 0.98]
DWI_wavelet_glcm_wavelet-LHH-Idn	0.836 [0.5 0.96]
DWI_wavelet_glcm_wavelet-LHH-Id	0.932 [0.64 0.98]
DWI_wavelet_glcm_wavelet-LHH-Idmn	0.938 [0.78 0.98]
DWI_wavelet_glcm_wavelet-LHH-Idm	0.93 [0.63 0.98]
DWI_wavelet_glcm_wavelet-HLL-Autocorrelation	0.955 [0.84 0.99]
DWI_wavelet_glcm_wavelet-HLL-JointAverage	0.954 [0.83 0.99]
DWI_wavelet_glcm_wavelet-HLL-ClusterProminence	0.899 [0.67 0.97]
DWI_wavelet_glcm_wavelet-HLL-ClusterTendency	0.979 [0.92 0.99]
DWI_wavelet_glcm_wavelet-HLL-Contrast	0.959 [0.84 0.99]
DWI_wavelet_glcm_wavelet-HLL-Correlation	0.952 [0.81 0.99]
DWI_wavelet_glcm_wavelet-HLL-DifferenceAverage	0.968 [0.88 0.99]
DWI_wavelet_glcm_wavelet-HLL-DifferenceEntropy	0.973 [0.9 0.99]
DWI_wavelet_glcm_wavelet-HLL-DifferenceVariance	0.936 [0.77 0.98]
DWI_wavelet_glcm_wavelet-HLL-JointEnergy	0.974 [0.9 0.99]
DWI_wavelet_glcm_wavelet-HLL-JointEntropy	0.978 [0.92 0.99]
DWI_wavelet_glcm_wavelet-HLL-InverseVariance	0.825 [0.45 0.95]

DWI_wavelet_glcm_wavelet-HLL-MaximumProbability	0.972	[0.9 0.99]
DWI_wavelet_glcm_wavelet-HLL-SumEntropy	0.982	[0.93 1.]
DWI_wavelet_glcm_wavelet-HLL-Idn	0.793	[0.35 0.94]
DWI_wavelet_glcm_wavelet-HLL-Id	0.965	[0.87 0.99]
DWI_wavelet_glcm_wavelet-HLL-Idmn	0.844	[0.49 0.96]
DWI_wavelet_glcm_wavelet-HLL-Idm	0.965	[0.87 0.99]
DWI_wavelet_glcm_wavelet-HLL-Imc2	0.807	[0.17 0.95]
DWI_wavelet_glcm_wavelet-HLH-Autocorrelation	0.872	[0.57 0.97]
DWI_wavelet_glcm_wavelet-HLH-JointAverage	0.899	[0.65 0.97]
DWI_wavelet_glcm_wavelet-HLH-ClusterProminence	0.99	[0.96 1.]
DWI_wavelet_glcm_wavelet-HLH-ClusterShade	0.906	[0.66 0.98]
DWI_wavelet_glcm_wavelet-HLH-ClusterTendency	0.97	[0.88 0.99]
DWI_wavelet_glcm_wavelet-HLH-Contrast	0.969	[0.87 0.99]
DWI_wavelet_glcm_wavelet-HLH-Correlation	0.876	[0.59 0.97]
DWI_wavelet_glcm_wavelet-HLH-DifferenceAverage	0.968	[0.86 0.99]
DWI_wavelet_glcm_wavelet-HLH-DifferenceEntropy	0.975	[0.89 0.99]
DWI_wavelet_glcm_wavelet-HLH-DifferenceVariance	0.973	[0.89 0.99]
DWI_wavelet_glcm_wavelet-HLH-JointEnergy	0.968	[0.87 0.99]
DWI_wavelet_glcm_wavelet-HLH-JointEntropy	0.971	[0.88 0.99]
DWI_wavelet_glcm_wavelet-HLH-InverseVariance	0.923	[0.72 0.98]
DWI_wavelet_glcm_wavelet-HLH-MaximumProbability	0.981	[0.9 1.]
DWI_wavelet_glcm_wavelet-HLH-SumEntropy	0.97	[0.88 0.99]
DWI_wavelet_glcm_wavelet-HLH-Idn	0.898	[0.66 0.97]
DWI_wavelet_glcm_wavelet-HLH-Id	0.97	[0.86 0.99]
DWI_wavelet_glcm_wavelet-HLH-Idmn	0.952	[0.82 0.99]
DWI_wavelet_glcm_wavelet-HLH-Idm	0.968	[0.86 0.99]
DWI_wavelet_glcm_wavelet-HLH-Imc2	0.99	[0.91 1.]
DWI_wavelet_glcm_wavelet-HLH-Imc1	0.942	[0.79 0.99]
DWI_wavelet_glcm_wavelet-HHL-Autocorrelation	0.94	[0.79 0.98]
DWI_wavelet_glcm_wavelet-HHL-JointAverage	0.896	[0.66 0.97]
DWI_wavelet_glcm_wavelet-HHL-ClusterProminence	0.97	[0.78 0.99]
DWI_wavelet_glcm_wavelet-HHL-ClusterTendency	0.978	[0.72 1.]
DWI_wavelet_glcm_wavelet-HHL-Contrast	0.984	[0.9 1.]
DWI_wavelet_glcm_wavelet-HHL-Correlation	0.881	[0.6 0.97]
DWI_wavelet_glcm_wavelet-HHL-DifferenceAverage	0.986	[0.94 1.]
DWI_wavelet_glcm_wavelet-HHL-DifferenceEntropy	0.981	[0.79 1.]
DWI_wavelet_glcm_wavelet-HHL-DifferenceVariance	0.98	[0.82 1.]
DWI_wavelet_glcm_wavelet-HHL-JointEnergy	0.983	[0.84 1.]
DWI_wavelet_glcm_wavelet-HHL-JointEntropy	0.98	[0.79 1.]
DWI_wavelet_glcm_wavelet-HHL-InverseVariance	0.908	[0.67 0.98]
DWI_wavelet_glcm_wavelet-HHL-MaximumProbability	0.973	[0.87 0.99]
DWI_wavelet_glcm_wavelet-HHL-SumEntropy	0.976	[0.73 1.]
DWI_wavelet_glcm_wavelet-HHL-Id	0.984	[0.94 1.]
DWI_wavelet_glcm_wavelet-HHL-Idm	0.986	[0.94 1.]
DWI_wavelet_glcm_wavelet-HHL-Imc2	0.956	[0.84 0.99]
DWI_wavelet_glcm_wavelet-HHL-Imc1	0.97	[0.89 0.99]
DWI_wavelet_glcm_wavelet-HHH-Autocorrelation	0.921	[0.73 0.98]
DWI_wavelet_glcm_wavelet-HHH-JointAverage	0.868	[0.58 0.96]
DWI_wavelet_glcm_wavelet-HHH-ClusterProminence	0.989	[0.95 1.]
DWI_wavelet_glcm_wavelet-HHH-ClusterShade	0.885	[0.59 0.97]
DWI_wavelet_glcm_wavelet-HHH-ClusterTendency	0.988	[0.93 1.]
DWI_wavelet_glcm_wavelet-HHH-Contrast	0.988	[0.95 1.]

DWI_wavelet_glcm_wavelet-HHH-DifferenceAverage	0.988	[0.95 1.]
DWI_wavelet_glcm_wavelet-HHH-DifferenceEntropy	0.987	[0.92 1.]
DWI_wavelet_glcm_wavelet-HHH-DifferenceVariance	0.985	[0.93 1.]
DWI_wavelet_glcm_wavelet-HHH-JointEnergy	0.984	[0.9 1.]
DWI_wavelet_glcm_wavelet-HHH-JointEntropy	0.988	[0.92 1.]
DWI_wavelet_glcm_wavelet-HHH-InverseVariance	0.895	[0.65 0.97]
DWI_wavelet_glcm_wavelet-HHH-MaximumProbability	0.931	[0.68 0.98]
DWI_wavelet_glcm_wavelet-HHH-SumEntropy	0.987	[0.91 1.]
DWI_wavelet_glcm_wavelet-HHH-Id	0.986	[0.95 1.]
DWI_wavelet_glcm_wavelet-HHH-Idm	0.989	[0.96 1.]
DWI_wavelet_glcm_wavelet-HHH-Imc2	0.976	[0.83 0.99]
DWI_wavelet_glcm_wavelet-HHH-Imc1	0.948	[0.63 0.99]
DWI_wavelet_glcm_wavelet-LLL-Autocorrelation	0.988	[0.95 1.]
DWI_wavelet_glcm_wavelet-LLL-JointAverage	0.98	[0.92 0.99]
DWI_wavelet_glcm_wavelet-LLL-ClusterProminence	0.989	[0.96 1.]
DWI_wavelet_glcm_wavelet-LLL-ClusterShade	0.974	[0.9 0.99]
DWI_wavelet_glcm_wavelet-LLL-ClusterTendency	0.981	[0.93 1.]
DWI_wavelet_glcm_wavelet-LLL-Contrast	0.978	[0.92 0.99]
DWI_wavelet_glcm_wavelet-LLL-DifferenceAverage	0.981	[0.93 1.]
DWI_wavelet_glcm_wavelet-LLL-DifferenceEntropy	0.987	[0.95 1.]
DWI_wavelet_glcm_wavelet-LLL-DifferenceVariance	0.981	[0.93 1.]
DWI_wavelet_glcm_wavelet-LLL-JointEnergy	0.934	[0.77 0.98]
DWI_wavelet_glcm_wavelet-LLL-JointEntropy	0.983	[0.93 1.]
DWI_wavelet_glcm_wavelet-LLL-InverseVariance	0.969	[0.89 0.99]
DWI_wavelet_glcm_wavelet-LLL-MaximumProbability	0.944	[0.8 0.99]
DWI_wavelet_glcm_wavelet-LLL-SumEntropy	0.972	[0.9 0.99]
DWI_wavelet_glcm_wavelet-LLL-Id	0.988	[0.95 1.]
DWI_wavelet_glcm_wavelet-LLL-Idm	0.989	[0.95 1.]
DWI_wavelet_glrlm_wavelet-LLH-GrayLevelNonUniformityNormaliz	0.978	[0.92 0.99]
DWI_wavelet_glrlm_wavelet-LLH-GrayLevelVariance	0.933	[0.7 0.98]
DWI_wavelet_glrlm_wavelet-LLH-HighGrayLevelRunEmphasis	0.914	[0.71 0.98]
DWI_wavelet_glrlm_wavelet-LLH-LongRunEmphasis	0.934	[0.76 0.98]
DWI_wavelet_glrlm_wavelet-LLH-LongRunHighGrayLevelEmphasis	0.863	[0.55 0.96]
DWI_wavelet_glrlm_wavelet-LLH-LongRunLowGrayLevelEmphasis	0.751	[0.3 0.93]
DWI_wavelet_glrlm_wavelet-LLH-RunEntropy	0.933	[0.75 0.98]
DWI_wavelet_glrlm_wavelet-LLH-RunLengthNonUniformity	0.928	[0.55 0.98]
DWI_wavelet_glrlm_wavelet-LLH-RunLengthNonUniformityNormaliz	0.877	[0.58 0.97]
DWI_wavelet_glrlm_wavelet-LLH-RunPercentage	0.883	[0.59 0.97]
DWI_wavelet_glrlm_wavelet-LLH-RunVariance	0.921	[0.71 0.98]
DWI_wavelet_glrlm_wavelet-LLH-ShortRunEmphasis	0.903	[0.65 0.98]
DWI_wavelet_glrlm_wavelet-LLH-ShortRunHighGrayLevelEmphasis	0.92	[0.73 0.98]
DWI_wavelet_glrlm_wavelet-LHL-GrayLevelNonUniformityNormaliz	0.978	[0.67 1.]
DWI_wavelet_glrlm_wavelet-LHL-GrayLevelVariance	0.881	[0.59 0.97]
DWI_wavelet_glrlm_wavelet-LHL-LongRunEmphasis	0.812	[0.4 0.95]
DWI_wavelet_glrlm_wavelet-LHL-RunLengthNonUniformity	0.89	[0.39 0.98]
DWI_wavelet_glrlm_wavelet-LHL-RunVariance	0.793	[0.35 0.94]
DWI_wavelet_glrlm_wavelet-LHH-GrayLevelNonUniformityNormaliz	0.95	[0.68 0.99]
DWI_wavelet_glrlm_wavelet-LHH-GrayLevelVariance	0.92	[0.6 0.98]
DWI_wavelet_glrlm_wavelet-LHH-LongRunLowGrayLevelEmphasis	0.969	[0.89 0.99]
DWI_wavelet_glrlm_wavelet-LHH-LowGrayLevelRunEmphasis	0.945	[0.81 0.99]
DWI_wavelet_glrlm_wavelet-LHH-RunLengthNonUniformity	0.882	[0.45 0.97]
DWI_wavelet_glrlm_wavelet-LHH-RunVariance	0.767	[0.29 0.94]

DWI_wavelet_glrlm_wavelet-LHH-ShortRunLowGrayLevelEmphasis	0.923	[0.74 0.98]
DWI_wavelet_glrlm_wavelet-HLL-GrayLevelNonUniformityNormaliz	0.989	[0.96 1.]
DWI_wavelet_glrlm_wavelet-HLL-GrayLevelVariance	0.958	[0.84 0.99]
DWI_wavelet_glrlm_wavelet-HLL-HighGrayLevelRunEmphasis	0.956	[0.84 0.99]
DWI_wavelet_glrlm_wavelet-HLL-LongRunEmphasis	0.794	[0.36 0.94]
DWI_wavelet_glrlm_wavelet-HLL-LongRunHighGrayLevelEmphasis	0.949	[0.82 0.99]
DWI_wavelet_glrlm_wavelet-HLL-LongRunLowGrayLevelEmphasis	0.917	[0.7 0.98]
DWI_wavelet_glrlm_wavelet-HLL-LowGrayLevelRunEmphasis	0.963	[0.86 0.99]
DWI_wavelet_glrlm_wavelet-HLL-RunEntropy	0.782	[0.34 0.94]
DWI_wavelet_glrlm_wavelet-HLL-RunLengthNonUniformity	0.899	[0.48 0.98]
DWI_wavelet_glrlm_wavelet-HLL-RunVariance	0.751	[0.27 0.93]
DWI_wavelet_glrlm_wavelet-HLL-ShortRunHighGrayLevelEmphasis	0.953	[0.83 0.99]
DWI_wavelet_glrlm_wavelet-HLL-ShortRunLowGrayLevelEmphasis	0.942	[0.78 0.99]
DWI_wavelet_glrlm_wavelet-HLH-GrayLevelNonUniformityNormaliz	0.981	[0.92 1.]
DWI_wavelet_glrlm_wavelet-HLH-GrayLevelVariance	0.991	[0.96 1.]
DWI_wavelet_glrlm_wavelet-HLH-HighGrayLevelRunEmphasis	0.876	[0.59 0.97]
DWI_wavelet_glrlm_wavelet-HLH-LongRunHighGrayLevelEmphasis	0.872	[0.57 0.97]
DWI_wavelet_glrlm_wavelet-HLH-LongRunLowGrayLevelEmphasis	0.99	[0.96 1.]
DWI_wavelet_glrlm_wavelet-HLH-LowGrayLevelRunEmphasis	0.99	[0.96 1.]
DWI_wavelet_glrlm_wavelet-HLH-RunEntropy	0.765	[0.32 0.94]
DWI_wavelet_glrlm_wavelet-HLH-RunLengthNonUniformity	0.892	[0.49 0.97]
DWI_wavelet_glrlm_wavelet-HLH-ShortRunHighGrayLevelEmphasis	0.874	[0.58 0.97]
DWI_wavelet_glrlm_wavelet-HLH-ShortRunLowGrayLevelEmphasis	0.982	[0.93 1.]
DWI_wavelet_glrlm_wavelet-HHL-GrayLevelNonUniformity	0.751	[0.27 0.93]
DWI_wavelet_glrlm_wavelet-HHL-GrayLevelNonUniformityNormaliz	0.987	[0.89 1.]
DWI_wavelet_glrlm_wavelet-HHL-GrayLevelVariance	0.985	[0.87 1.]
DWI_wavelet_glrlm_wavelet-HHL-HighGrayLevelRunEmphasis	0.94	[0.79 0.98]
DWI_wavelet_glrlm_wavelet-HHL-LongRunHighGrayLevelEmphasis	0.886	[0.62 0.97]
DWI_wavelet_glrlm_wavelet-HHL-LongRunLowGrayLevelEmphasis	0.954	[0.83 0.99]
DWI_wavelet_glrlm_wavelet-HHL-RunLengthNonUniformity	0.846	[0.4 0.96]
DWI_wavelet_glrlm_wavelet-HHL-ShortRunHighGrayLevelEmphasis	0.958	[0.84 0.99]
DWI_wavelet_glrlm_wavelet-HHH-GrayLevelNonUniformityNormaliz	0.992	[0.97 1.]
DWI_wavelet_glrlm_wavelet-HHH-GrayLevelVariance	0.991	[0.97 1.]
DWI_wavelet_glrlm_wavelet-HHH-HighGrayLevelRunEmphasis	0.923	[0.74 0.98]
DWI_wavelet_glrlm_wavelet-HHH-LongRunHighGrayLevelEmphasis	0.832	[0.47 0.96]
DWI_wavelet_glrlm_wavelet-HHH-LongRunLowGrayLevelEmphasis	0.925	[0.74 0.98]
DWI_wavelet_glrlm_wavelet-HHH-RunLengthNonUniformity	0.843	[0.4 0.96]
DWI_wavelet_glrlm_wavelet-HHH-ShortRunHighGrayLevelEmphasis	0.945	[0.81 0.99]
DWI_wavelet_glrlm_wavelet-LLL-GrayLevelNonUniformityNormaliz	0.939	[0.79 0.98]
DWI_wavelet_glrlm_wavelet-LLL-GrayLevelVariance	0.995	[0.98 1.]
DWI_wavelet_glrlm_wavelet-LLL-HighGrayLevelRunEmphasis	0.991	[0.96 1.]
DWI_wavelet_glrlm_wavelet-LLL-LongRunEmphasis	0.827	[0.44 0.95]
DWI_wavelet_glrlm_wavelet-LLL-LongRunHighGrayLevelEmphasis	0.987	[0.95 1.]
DWI_wavelet_glrlm_wavelet-LLL-LongRunLowGrayLevelEmphasis	0.953	[0.83 0.99]
DWI_wavelet_glrlm_wavelet-LLL-RunEntropy	0.87	[0.57 0.97]
DWI_wavelet_glrlm_wavelet-LLL-RunLengthNonUniformity	0.933	[0.54 0.99]
DWI_wavelet_glrlm_wavelet-LLL-RunLengthNonUniformityNormaliz	0.885	[0.6 0.97]
DWI_wavelet_glrlm_wavelet-LLL-RunPercentage	0.853	[0.51 0.96]
DWI_wavelet_glrlm_wavelet-LLL-RunVariance	0.802	[0.37 0.95]
DWI_wavelet_glrlm_wavelet-LLL-ShortRunEmphasis	0.885	[0.6 0.97]
DWI_wavelet_glrlm_wavelet-LLL-ShortRunHighGrayLevelEmphasis	0.991	[0.96 1.]
DWI_wavelet_glszm_wavelet-LLH-GrayLevelNonUniformity	0.778	[0.23 0.94]

DWI_wavelet_glszm_wavelet-LLH-GrayLevelNonUniformityNormaliz	0.911	[0.7 0.98]
DWI_wavelet_glszm_wavelet-LLH-GrayLevelVariance	0.976	[0.9 0.99]
DWI_wavelet_glszm_wavelet-LLH-HighGrayLevelZoneEmphasis	0.909	[0.69 0.98]
DWI_wavelet_glszm_wavelet-LLH-LargeAreaEmphasis	0.835	[0.47 0.96]
DWI_wavelet_glszm_wavelet-LLH-LargeAreaLowGrayLevelEmphasis	0.828	[0.47 0.95]
DWI_wavelet_glszm_wavelet-LLH-SizeZoneNonUniformity	0.931	[0.58 0.98]
DWI_wavelet_glszm_wavelet-LLH-SizeZoneNonUniformityNormalize	0.835	[0.48 0.96]
DWI_wavelet_glszm_wavelet-LLH-SmallAreaEmphasis	0.846	[0.49 0.96]
DWI_wavelet_glszm_wavelet-LLH-SmallAreaHighGrayLevelEmphasis	0.892	[0.65 0.97]
DWI_wavelet_glszm_wavelet-LLH-ZoneEntropy	0.976	[0.91 0.99]
DWI_wavelet_glszm_wavelet-LLH-ZonePercentage	0.819	[0.45 0.95]
DWI_wavelet_glszm_wavelet-LLH-ZoneVariance	0.83	[0.46 0.95]
DWI_wavelet_glszm_wavelet-LHL-GrayLevelNonUniformity	0.975	[0.44 1.]
DWI_wavelet_glszm_wavelet-LHL-LargeAreaEmphasis	0.911	[0.68 0.98]
DWI_wavelet_glszm_wavelet-LHL-SizeZoneNonUniformity	0.915	[0.2 0.98]
DWI_wavelet_glszm_wavelet-LHL-ZoneEntropy	0.891	[0.64 0.97]
DWI_wavelet_glszm_wavelet-LHL-ZonePercentage	0.843	[0.51 0.96]
DWI_wavelet_glszm_wavelet-LHL-ZoneVariance	0.902	[0.65 0.97]
DWI_wavelet_glszm_wavelet-LHH-GrayLevelNonUniformity	0.99	[0.92 1.]
DWI_wavelet_glszm_wavelet-LHH-GrayLevelVariance	0.789	[0.38 0.94]
DWI_wavelet_glszm_wavelet-LHH-LargeAreaEmphasis	0.819	[0.43 0.95]
DWI_wavelet_glszm_wavelet-LHH-LargeAreaLowGrayLevelEmphasis	0.844	[0.51 0.96]
DWI_wavelet_glszm_wavelet-LHH-SizeZoneNonUniformity	0.928	[0.64 0.98]
DWI_wavelet_glszm_wavelet-LHH-SmallAreaEmphasis	0.874	[0.59 0.97]
DWI_wavelet_glszm_wavelet-LHH-ZoneEntropy	0.865	[0.56 0.96]
DWI_wavelet_glszm_wavelet-LHH-ZonePercentage	0.797	[0.38 0.95]
DWI_wavelet_glszm_wavelet-HLL-GrayLevelNonUniformity	0.911	[0.51 0.98]
DWI_wavelet_glszm_wavelet-HLL-GrayLevelVariance	0.927	[0.75 0.98]
DWI_wavelet_glszm_wavelet-HLL-HighGrayLevelZoneEmphasis	0.963	[0.87 0.99]
DWI_wavelet_glszm_wavelet-HLL-LargeAreaEmphasis	0.954	[0.83 0.99]
DWI_wavelet_glszm_wavelet-HLL-LargeAreaHighGrayLevelEmphasis	0.915	[0.7 0.98]
DWI_wavelet_glszm_wavelet-HLL-LargeAreaLowGrayLevelEmphasis	0.964	[0.86 0.99]
DWI_wavelet_glszm_wavelet-HLL-LowGrayLevelZoneEmphasis	0.92	[0.71 0.98]
DWI_wavelet_glszm_wavelet-HLL-SizeZoneNonUniformity	0.879	[0.22 0.97]
DWI_wavelet_glszm_wavelet-HLL-SmallAreaHighGrayLevelEmphasis	0.863	[0.56 0.96]
DWI_wavelet_glszm_wavelet-HLL-SmallAreaLowGrayLevelEmphasis	0.827	[0.47 0.95]
DWI_wavelet_glszm_wavelet-HLL-ZoneEntropy	0.987	[0.95 1.]
DWI_wavelet_glszm_wavelet-HLL-ZoneVariance	0.947	[0.8 0.99]
DWI_wavelet_glszm_wavelet-HLH-GrayLevelNonUniformity	0.96	[0.59 0.99]
DWI_wavelet_glszm_wavelet-HLH-GrayLevelNonUniformityNormaliz	0.783	[0.33 0.94]
DWI_wavelet_glszm_wavelet-HLH-GrayLevelVariance	0.904	[0.66 0.98]
DWI_wavelet_glszm_wavelet-HLH-HighGrayLevelZoneEmphasis	0.882	[0.6 0.97]
DWI_wavelet_glszm_wavelet-HLH-LargeAreaEmphasis	0.992	[0.97 1.]
DWI_wavelet_glszm_wavelet-HLH-LargeAreaHighGrayLevelEmphasis	0.974	[0.9 0.99]
DWI_wavelet_glszm_wavelet-HLH-LargeAreaLowGrayLevelEmphasis	0.994	[0.98 1.]
DWI_wavelet_glszm_wavelet-HLH-LowGrayLevelZoneEmphasis	0.867	[0.56 0.97]
DWI_wavelet_glszm_wavelet-HLH-SizeZoneNonUniformity	0.923	[0.57 0.98]
DWI_wavelet_glszm_wavelet-HLH-SizeZoneNonUniformityNormalize	0.833	[0.46 0.96]
DWI_wavelet_glszm_wavelet-HLH-SmallAreaHighGrayLevelEmphasis	0.913	[0.69 0.98]
DWI_wavelet_glszm_wavelet-HLH-SmallAreaLowGrayLevelEmphasis	0.831	[0.45 0.96]
DWI_wavelet_glszm_wavelet-HLH-ZoneEntropy	0.985	[0.95 1.]
DWI_wavelet_glszm_wavelet-HLH-ZonePercentage	0.797	[0.36 0.95]

DWI_wavelet_glszm_wavelet-HLH-ZoneVariance	0.94	[0.78 0.98]
DWI_wavelet_glszm_wavelet-HHL-GrayLevelNonUniformity	0.98	[0.92 0.99]
DWI_wavelet_glszm_wavelet-HHL-GrayLevelVariance	0.856	[0.54 0.96]
DWI_wavelet_glszm_wavelet-HHL-HighGrayLevelZoneEmphasis	0.788	[0.38 0.94]
DWI_wavelet_glszm_wavelet-HHL-LargeAreaEmphasis	0.845	[0.5 0.96]
DWI_wavelet_glszm_wavelet-HHL-LargeAreaLowGrayLevelEmphasis	0.97	[0.88 0.99]
DWI_wavelet_glszm_wavelet-HHL-SizeZoneNonUniformity	0.974	[0.88 0.99]
DWI_wavelet_glszm_wavelet-HHL-ZonePercentage	0.831	[0.45 0.96]
DWI_wavelet_glszm_wavelet-HHH-GrayLevelNonUniformity	0.975	[0.9 0.99]
DWI_wavelet_glszm_wavelet-HHH-GrayLevelVariance	0.893	[0.63 0.97]
DWI_wavelet_glszm_wavelet-HHH-HighGrayLevelZoneEmphasis	0.839	[0.5 0.96]
DWI_wavelet_glszm_wavelet-HHH-LargeAreaEmphasis	0.766	[0.29 0.94]
DWI_wavelet_glszm_wavelet-HHH-LargeAreaLowGrayLevelEmphasis	0.833	[0.48 0.96]
DWI_wavelet_glszm_wavelet-HHH-SizeZoneNonUniformity	0.96	[0.84 0.99]
DWI_wavelet_glszm_wavelet-HHH-SmallAreaLowGrayLevelEmphasis	0.833	[0.49 0.96]
DWI_wavelet_glszm_wavelet-HHH-ZonePercentage	0.944	[0.79 0.99]
DWI_wavelet_glszm_wavelet-LLL-GrayLevelNonUniformity	0.856	[-0.02 0.97]
DWI_wavelet_glszm_wavelet-LLL-GrayLevelVariance	0.978	[0.92 0.99]
DWI_wavelet_glszm_wavelet-LLL-HighGrayLevelZoneEmphasis	0.987	[0.95 1.]
DWI_wavelet_glszm_wavelet-LLL-LargeAreaLowGrayLevelEmphasis	0.816	[0.41 0.95]
DWI_wavelet_glszm_wavelet-LLL-SizeZoneNonUniformity	0.979	[0.32 1.]
DWI_wavelet_glszm_wavelet-LLL-SizeZoneNonUniformityNormalize	0.966	[0.86 0.99]
DWI_wavelet_glszm_wavelet-LLL-SmallAreaEmphasis	0.83	[0.47 0.95]
DWI_wavelet_glszm_wavelet-LLL-SmallAreaHighGrayLevelEmphasis	0.985	[0.94 1.]
DWI_wavelet_glszm_wavelet-LLL-ZoneEntropy	0.831	[0.48 0.95]
DWI_wavelet_glszm_wavelet-LLL-ZonePercentage	0.953	[0.83 0.99]
DWI_wavelet_gldm_wavelet-LLH-DependenceEntropy	0.975	[0.81 0.99]
DWI_wavelet_gldm_wavelet-LLH-DependenceNonUniformity	0.894	[0.5 0.97]
DWI_wavelet_gldm_wavelet-LLH-DependenceNonUniformityNormaliz	0.865	[0.57 0.96]
DWI_wavelet_gldm_wavelet-LLH-DependenceVariance	0.979	[0.92 0.99]
DWI_wavelet_gldm_wavelet-LLH-GrayLevelVariance	0.931	[0.69 0.98]
DWI_wavelet_gldm_wavelet-LLH-HighGrayLevelEmphasis	0.914	[0.71 0.98]
DWI_wavelet_gldm_wavelet-LLH-LargeDependenceEmphasis	0.905	[0.66 0.98]
DWI_wavelet_gldm_wavelet-LLH-LargeDependenceHighGrayLevelEmp	0.787	[0.35 0.94]
DWI_wavelet_gldm_wavelet-LLH-LargeDependenceLowGrayLevelEmph	0.756	[0.32 0.93]
DWI_wavelet_gldm_wavelet-LLH-SmallDependenceEmphasis	0.874	[0.59 0.97]
DWI_wavelet_gldm_wavelet-LLH-SmallDependenceHighGrayLevelEmp	0.885	[0.61 0.97]
DWI_wavelet_gldm_wavelet-LHL-DependenceEntropy	0.862	[0.51 0.96]
DWI_wavelet_gldm_wavelet-LHL-DependenceNonUniformity	0.812	[0.41 0.95]
DWI_wavelet_gldm_wavelet-LHL-GrayLevelVariance	0.904	[0.67 0.98]
DWI_wavelet_gldm_wavelet-LHH-DependenceEntropy	0.887	[0.05 0.98]
DWI_wavelet_gldm_wavelet-LHH-DependenceNonUniformity	0.751	[0.3 0.93]
DWI_wavelet_gldm_wavelet-LHH-DependenceNonUniformityNormaliz	0.91	[0.55 0.98]
DWI_wavelet_gldm_wavelet-LHH-DependenceVariance	0.908	[0.53 0.98]
DWI_wavelet_gldm_wavelet-LHH-GrayLevelVariance	0.919	[0.56 0.98]
DWI_wavelet_gldm_wavelet-LHH-LargeDependenceLowGrayLevelEmph	0.963	[0.86 0.99]
DWI_wavelet_gldm_wavelet-LHH-LowGrayLevelEmphasis	0.951	[0.83 0.99]
DWI_wavelet_gldm_wavelet-HLL-DependenceEntropy	0.916	[0.72 0.98]
DWI_wavelet_gldm_wavelet-HLL-DependenceNonUniformity	0.805	[0.39 0.95]
DWI_wavelet_gldm_wavelet-HLL-DependenceVariance	0.753	[0.28 0.93]
DWI_wavelet_gldm_wavelet-HLL-GrayLevelVariance	0.961	[0.85 0.99]
DWI_wavelet_gldm_wavelet-HLL-HighGrayLevelEmphasis	0.955	[0.84 0.99]

DWI_wavelet_gldm_wavelet-HLL-LargeDependenceHighGrayLevelEmp	0.95 [0.82 0.99]
DWI_wavelet_gldm_wavelet-HLL-LargeDependenceLowGrayLevelEmph	0.879 [0.59 0.97]
DWI_wavelet_gldm_wavelet-HLL-LowGrayLevelEmphasis	0.968 [0.88 0.99]
DWI_wavelet_gldm_wavelet-HLL-SmallDependenceHighGrayLevelEmp	0.858 [0.55 0.96]
DWI_wavelet_gldm_wavelet-HLL-SmallDependenceLowGrayLevelEmph	0.791 [0.38 0.94]
DWI_wavelet_gldm_wavelet-HLH-DependenceEntropy	0.981 [0.62 1.]
DWI_wavelet_gldm_wavelet-HLH-DependenceNonUniformityNormaliz	0.925 [0.73 0.98]
DWI_wavelet_gldm_wavelet-HLH-DependenceVariance	0.952 [0.83 0.99]
DWI_wavelet_gldm_wavelet-HLH-GrayLevelVariance	0.985 [0.93 1.]
DWI_wavelet_gldm_wavelet-HLH-HighGrayLevelEmphasis	0.875 [0.58 0.97]
DWI_wavelet_gldm_wavelet-HLH-LargeDependenceHighGrayLevelEmp	0.879 [0.59 0.97]
DWI_wavelet_gldm_wavelet-HLH-LargeDependenceLowGrayLevelEmph	0.986 [0.95 1.]
DWI_wavelet_gldm_wavelet-HLH-LowGrayLevelEmphasis	0.991 [0.96 1.]
DWI_wavelet_gldm_wavelet-HLH-SmallDependenceHighGrayLevelEmp	0.886 [0.61 0.97]
DWI_wavelet_gldm_wavelet-HHL-DependenceEntropy	0.853 [-0.04 0.97]
DWI_wavelet_gldm_wavelet-HHL-DependenceNonUniformity	0.802 [0.4 0.95]
DWI_wavelet_gldm_wavelet-HHL-DependenceNonUniformityNormaliz	0.881 [0.36 0.97]
DWI_wavelet_gldm_wavelet-HHL-DependenceVariance	0.895 [0.04 0.98]
DWI_wavelet_gldm_wavelet-HHL-GrayLevelNonUniformity	0.765 [0.31 0.94]
DWI_wavelet_gldm_wavelet-HHL-GrayLevelVariance	0.982 [0.86 1.]
DWI_wavelet_gldm_wavelet-HHL-HighGrayLevelEmphasis	0.94 [0.79 0.98]
DWI_wavelet_gldm_wavelet-HHL-LargeDependenceHighGrayLevelEmp	0.882 [0.6 0.97]
DWI_wavelet_gldm_wavelet-HHL-LargeDependenceLowGrayLevelEmph	0.979 [0.92 0.99]
DWI_wavelet_gldm_wavelet-HHL-SmallDependenceHighGrayLevelEmp	0.976 [0.87 0.99]
DWI_wavelet_gldm_wavelet-HHH-DependenceEntropy	0.885 [0.11 0.98]
DWI_wavelet_gldm_wavelet-HHH-DependenceNonUniformity	0.805 [0.41 0.95]
DWI_wavelet_gldm_wavelet-HHH-DependenceNonUniformityNormaliz	0.87 [0.58 0.97]
DWI_wavelet_gldm_wavelet-HHH-DependenceVariance	0.929 [0.5 0.98]
DWI_wavelet_gldm_wavelet-HHH-GrayLevelNonUniformity	0.766 [0.31 0.94]
DWI_wavelet_gldm_wavelet-HHH-GrayLevelVariance	0.988 [0.95 1.]
DWI_wavelet_gldm_wavelet-HHH-HighGrayLevelEmphasis	0.922 [0.74 0.98]
DWI_wavelet_gldm_wavelet-HHH-LargeDependenceHighGrayLevelEmp	0.831 [0.47 0.95]
DWI_wavelet_gldm_wavelet-HHH-LargeDependenceLowGrayLevelEmph	0.96 [0.85 0.99]
DWI_wavelet_gldm_wavelet-HHH-SmallDependenceHighGrayLevelEmp	0.98 [0.91 1.]
DWI_wavelet_gldm_wavelet-LLL-DependenceEntropy	0.795 [0.38 0.94]
DWI_wavelet_gldm_wavelet-LLL-DependenceNonUniformity	0.925 [0.54 0.98]
DWI_wavelet_gldm_wavelet-LLL-DependenceNonUniformityNormaliz	0.79 [0.34 0.94]
DWI_wavelet_gldm_wavelet-LLL-GrayLevelVariance	0.994 [0.98 1.]
DWI_wavelet_gldm_wavelet-LLL-HighGrayLevelEmphasis	0.991 [0.96 1.]
DWI_wavelet_gldm_wavelet-LLL-LargeDependenceEmphasis	0.783 [0.33 0.94]
DWI_wavelet_gldm_wavelet-LLL-LargeDependenceHighGrayLevelEmp	0.969 [0.88 0.99]
DWI_wavelet_gldm_wavelet-LLL-LargeDependenceLowGrayLevelEmph	0.962 [0.86 0.99]
DWI_wavelet_gldm_wavelet-LLL-SmallDependenceEmphasis	0.966 [0.85 0.99]
DWI_wavelet_gldm_wavelet-LLL-SmallDependenceHighGrayLevelEmp	0.993 [0.96 1.]
DWI_wavelet_ngtdm_wavelet-LLH-Complexity	0.943 [0.71 0.99]
DWI_wavelet_ngtdm_wavelet-LLH-Strength	0.951 [0.82 0.99]
DWI_wavelet_ngtdm_wavelet-LHH-Busyness	0.991 [0.97 1.]
DWI_wavelet_ngtdm_wavelet-LHH-Contrast	0.972 [0.9 0.99]
DWI_wavelet_ngtdm_wavelet-HLL-Complexity	0.888 [0.62 0.97]
DWI_wavelet_ngtdm_wavelet-HLH-Busyness	0.915 [0.71 0.98]
DWI_wavelet_ngtdm_wavelet-HLH-Complexity	0.967 [0.87 0.99]
DWI_wavelet_ngtdm_wavelet-HLH-Contrast	0.953 [0.83 0.99]

DWI_wavelet_ngtdm_wavelet-HHL-Busyness	0.992	[0.97 1.]
DWI_wavelet_ngtdm_wavelet-HHL-Complexity	0.857	[0.5 0.96]
DWI_wavelet_ngtdm_wavelet-HHL-Strength	0.914	[0.69 0.98]
DWI_wavelet_ngtdm_wavelet-HHH-Busyness	0.992	[0.97 1.]
DWI_wavelet_ngtdm_wavelet-HHH-Complexity	0.945	[0.81 0.99]
DWI_wavelet_ngtdm_wavelet-HHH-Strength	0.944	[0.68 0.99]
DWI_wavelet_ngtdm_wavelet-LLL-Complexity	0.97	[0.89 0.99]
DWI_wavelet_ngtdm_wavelet-LLL-Contrast	0.849	[0.53 0.96]
DWI_wavelet_ngtdm_wavelet-LLL-Strength	0.872	[0.57 0.97]
